# Network meta-analysis of randomized control trials evaluating the effectiveness of various probiotic formulations in patients with type 2 diabetes mellitus

**DOI:** 10.1186/s13098-025-01841-2

**Published:** 2025-07-11

**Authors:** Abdallah R. Allam, Mohamed Basyouni Helal, Mohamed Salah Alhateem, Mohamed Ashraf Shehab, Ahmed Gamal Elshaar, Mohamed Ahmed Saeda, Fatma Magdi Ibrahim, Ahmed Maher Khalil, Abdelrahman Mohamed Mahmoud

**Affiliations:** 1 Faculty of Medicine, Menoufia University, Yassin Abdelghaffar Street From Gamal Abdelnaser Street, Shebin Al-Kom, 32511 Menoufia Egypt; 2 RAK Medical and Health Sciences University, RAK College of Nursing, RAS-Al Khaimah, UAE; 3 Maxillofacial Department, Royal Derby Hospital, Derby, UK

**Keywords:** Type 2 diabetes mellitus, Fasting plasma glucose, Hemoglobin A1c, Probiotics, Network meta-analysis

## Abstract

**Background:**

Type 2 diabetes mellitus (T2DM) is a global health concern with a rising prevalence. Recent studies indicate that the gut microbiota plays a role in the development of T2DM, making probiotics a promising avenue for proper glycemic control. This network meta-analysis aims to comprehensively assess various probiotic therapies in the management of T2DM.

**Methods:**

All published randomized controlled trials (RCTs) were identified through searches in PubMed, Cochrane, and Web of Science. Included RCTs were evaluated using the Cochrane risk of bias tool 2. The retrieved data were pooled, where the mean difference (MD) and a 95% confidence interval (CI) were calculated using the R program version 4.2.1.

**Results:**

A total of 62 RCTs were included, with baseline hemoglobin A1c (HbA1c) ranging from 5.71% to 9.5%. Among all interventions, the combination of Bifidobacterium bifidum W23, Bifidobacterium lactis W52, Lactobacillus acidophilus W37, Lactobacillus brevis W63, Lactobacillus casei W56, Lactobacillus salivarius W24, Lactococcus lactis W19, and Lactococcus lactis W58 achieved the greatest reduction in fasting plasma glucose (FPG) [MD = − 73.50; 95% CI: − 113.13 to − 33.86]. The greatest reduction in HbA1c was observed with yogurt containing Lactobacillus acidophilus La5, Bifidobacterium Bb12, and Cucurbita ficifolia [MD = − 1.59; 95% CI: − 3.07 to − 0.12]. For total cholesterol, the top-performing intervention was yeast containing Saccharomyces cerevisiae [MD = − 43.67; 95% CI: − 57.07 to − 30.27].

**Conclusion:**

Our network meta-analysis suggests that specific probiotic formulations, including combinations of various strains of Lactobacillus, Bifidobacterium, yogurt-based formulations containing Lactobacillus acidophilus La5, Bifidobacterium Bb12 with Cucurbita ficifolia, and Saccharomyces cerevisiae yeast, may be beneficial as adjunctive therapies for improving glycemic control and lipid profiles in patients with T2DM. However, these findings should be interpreted with caution due to variability in study designs, probiotic regimens, and potential risk of bias.

**Supplementary Information:**

The online version contains supplementary material available at 10.1186/s13098-025-01841-2.

## Introduction

Type 2 diabetes mellitus (T2DM) is a common metabolic disorder characterized by elevated blood glucose due to either insulin deficiency, insulin resistance, or both (1). About 463 million (9.3%) of adults worldwide (aged 20 to 79) had diabetes in 2019 according to a recent report by the International Diabetes Federation (IDF). This number is expected to go up to 578 million (10.2%) in 2030 and 700 million (10.9%) in 2045 (2). Approximately 90% to 95% of cases are T2DM (3). Genetic predisposition, obesity, physical inactivity, dyslipidemia, and hypertension are reported to be involved in the etiology of T2DM (1, 4). Uncontrolled T2DM can lead to several health threats such as ischemic heart disease, stroke, nephropathy, retinopathy, and lower-limb amputation (5). Proper glycemic control can help to prevent or reduce these diabetic complications (6).

Drugs used to treat T2DM nowadays have many serious adverse effects (7). Therefore, there is still a need for safer and more efficient hypoglycemic medications (8). Recently, there has been experimental and clinical evidence supporting that blood glucose imbalance is associated with the alteration of gut microbiota (also known as dysbiosis). Dysbiosis is considered a key environmental factor in the pathogenesis of T2DM (9–11). It can disturb the glucose homeostasis of the host by several mechanisms; especially increased gut permeability and translocation of lipopolysaccharide (LPS) to the circulation inducing “metabolic endotoxemia” which leads to inflammatory responses, cytokines release, and insulin resistance (12). Other suggested reasons are gastrointestinal peptide hormone secretion and the production of metabolites by bacteria during fermentation (13).

One of the most effective ways to modulate the gut microbiota and maintain its balance is the use of probiotics (14). According to the World Health Organization (WHO), probiotics are defined as “live microorganisms which when administered in adequate amounts confer a health benefit on the host” (15). Several animal and human studies have revealed that probiotics have a positive impact on glycemic control; improving insulin sensitivity, reducing insulin resistance, regulating blood glucose levels, lowering blood lipids, and delaying or preventing the development of T2DM and its complications (10, 16–21).

Previous systematic reviews and traditional meta-analyses have investigated the impact of various probiotic strains on glycemic control and related parameters of T2DM and related parameters, including glycated hemoglobin, fasting blood glucose, insulin resistance, and body weight (13, 22–33). Network meta-analysis (NMA) extends traditional meta-analytic techniques by combining both direct and indirect evidence across a network of studies, enabling a comprehensive comparison of multiple interventions even when head-to-head trials are lacking. Therefore, this NMA aims to fill this critical gap by synthesizing existing evidence to rank the comparative effectiveness of different probiotic therapies in patients with T2DM, thereby informing future clinical practice and research.

## Materials and methods

We followed the "Preferred Reporting Items for Systematic Reviews and Meta-Analyses (PRISMA)" guidelines while conducting this study (34). Additionally, we adhered to the guidelines in the “Cochrane Handbook for Systematic Reviews of Interventions” (35).

### Literature search and screening

We performed a systematic search of PubMed, Web of Science, and Cochrane from database inception. The search strategy included keywords and MeSH terms related to “probiotics,” “type 2 diabetes mellitus,”. The detailed search strategy is described in *supplementary file 4.* In order to find pertinent RCTS for NMA, we carried out a four-step screening procedure. In the initial phase, we collected all retrieved articles and eliminated duplicates using Endnote 20 software. Next, the articles’ titles and abstracts were screened to rule out irrelevant studies. In the third step, we examined the complete texts of the remaining articles to determine whether they qualified for the review. Finally, we looked over the reference lists of the articles that were included to identify any potentially relevant publications that were missed in the initial search. The screening process was conducted independently by two researchers, and any disagreements were resolved by a third researcher.

### Eligibility criteria

We include RCTs evaluating the effectiveness of probiotics in T2DM. Observational studies, case reports, case series, conference abstracts, animal, in-vitro experiments, and non-English articles were excluded.

### Data extraction

The extracted data included an overview of the studies that were included as well as baseline characteristics such as age, sex, weight, disease duration, FPG, HbA1c, total cholesterol, low- and high-density lipoprotein (LDL and HDL, respectively), and triacylglycerol (TAG).

FPG, HbA1c, insulin resistance, total cholesterol, LDL, HDL, TAG, and weight gain were the main outcomes. The secondary outcomes included adipocytokines [leptin, adiponectin, and resistin], insulin concentration, fasting insulin, postprandial glucose (PPG), cholesterol/HDL ratio, inflammatory markers [tumor necrosis factor-alpha (TNF-α), interleukin-6 (IL-6), and C-reactive protein (CRP], and fat mass.

### Risk of bias assessment

We used the Cochrane risk of bias tool (ROB2), (36) for RCTs which has six bias domains [randomization process, deviations from intended treatments, missing outcome data, measurement of outcomes, selection of reported outcomes, and overall risk of bias]. The bias of the included studies was evaluated by two authors, and any disagreements were settled by a third author.

### Statistical analysis

The R program version 4.2.1 was used to carry out network meta-analysis through meta and netmeta packages. Treatment groups were classified according to the specific probiotic formulations administered in each study. Each formulation, whether a single strain or a defined combination of strains, was treated as a distinct intervention. The placebo, regular yogurt, control diet, and other passive comparators (such as no treatment or standard care without active supplementation) were all combined into one control group. Using the mean difference (MD) and a 95% confidence interval (CI), we combined continuous results. The pooled studies’ heterogeneity was assessed using the Chi-square and I^2^ tests, and relationships between the studies were considered heterogeneous when the Chi-square p < 0.1 and the I^2^ > 50%. For pooling homogeneous data, a fixed-effect model was utilized, while for pooling heterogeneous data, we used random-effect models. Meta-regression analysis was conducted to determine if the age and baseline HbA1c have influenced the study outcomes.

## Results

### Data collection and characteristics of included studies

**Fig. 1:**
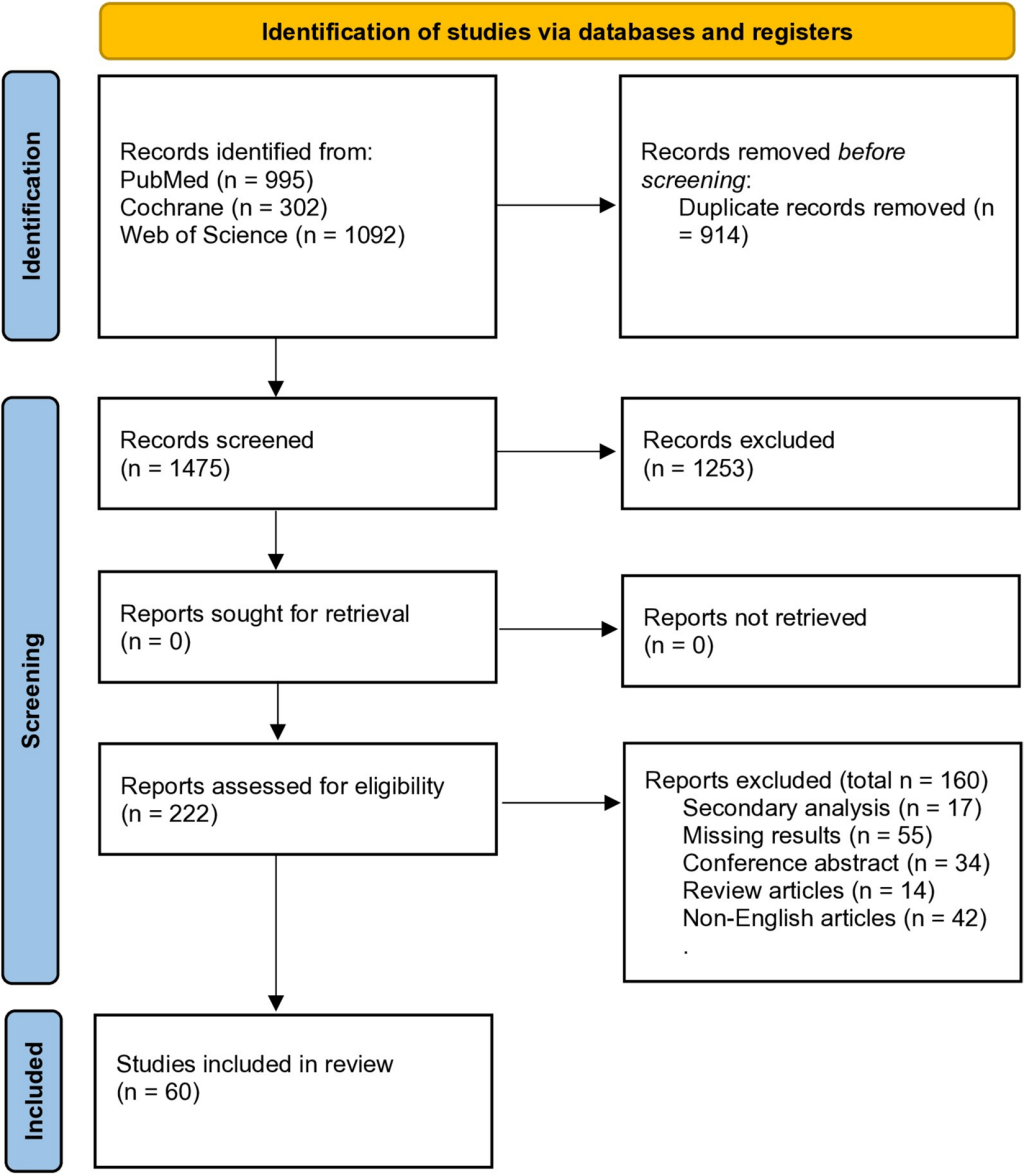
PRISMA

Database searches turned up 2389 publications, including 302 from Cochrane, 1092 from WOS, and 995 from PubMed. We reviewed 1475 items and deleted 914 duplicates. 1253 articles were excluded after the title and abstract screening. Out of the remaining 222 articles, 162 were excluded for reasons such as inadequate outcome reporting, insufficient data for analysis, inappropriate control groups, or non-eligible probiotic formulations. only 60 relevant studies (10, 11, 19, 20, 37–51, 53–93) who met all inclusion criteria were included after the full-text screening. A flowchart of the database search and study selection procedure is shown in Fig. 1.

The sample sizes of the included RCTs ranged from 20 to 409 patients. The age of the included patients ranged from 43.77 to 53.6 years, and the diabetes duration at inclusion ranged between 0.32 and 18.1 years. At baseline, the HbA1c ranged from 5.71% to 9.5%. The dosages varied considerably across studies, ranging from 100 mg/day up to 300 g/day for yogurt-based preparation, or 100 to 600 ml/day for milk based preparations. Several studies used capsule-based probiotics containing multiple strains, while others incorporated probiotics into bread, honey, or skim milk powder. The most commonly used probiotic genera included *Lactobacillus* (e.g., *L. acidophilus*, *L. casei*, *L. rhamnosus*, *L. plantarum*), *Bifidobacterium* (e.g., *B. breve*, *B. bifidum*, *B. lactis*), and *Streptococcus thermophilus*. Some formulations also included yeast species such as *Saccharomyces cerevisiae* or *S. boulardii*, or were combined with other agents like metformin, selenium, vitamin D3, berberine, or smectite gel. Multi-strain preparations were frequently used, with some products containing up to eight distinct probiotic strains. The duration of probiotic administration ranged from 4 to 24 weeks, with follow-up durations often matching the intervention period but extending up to 36 weeks in some studies. The summary of the included studies and participants’ baseline characteristics are summarized in Tables 1 and 2; respectively.

**Table 1:** Summary of included studies

ID	Country	Registration	Sample size, n	Type of Probiotics	Inclusion criteria	Exclusion criteria	Conclusion
Abbasi 2018	Iran	IRCT201601027479N2	40	Soy milk contained lactobacillus plantarum A7	DM type 2 proved for more than a year, age 25 or older, microalbuminuria, and GFR greater than 60 ml/min. FPG greater than 126 mg/dl. 2h PPG greater than 200 mg/dl	Subjects who had recently had antibiotic medication, IBD, infection, liver disease, RA, smoking, alcoholism, or who had taken multivitamins, mineral supplements, or omega-3 fatty acids one month before the start of the intervention	In conclusion, ingestion of soy milk with Lactobacillus plantarum A7 was safe, well-tolerated, and had positive effects on LDL, Total Cholesterol, HDL, and serum TGA as well as enhanced renal function in type 2 diabetes patients with nephropathy. Findings from our study point to the possibility that lactobacillus plantarum-containing soy milk can help diabetic patients with their lipid profiles and slow the development of nephropathy
Alihosseini 2017	Iran	IRCT201307092017N14	60	Fermented milk contained streptococcus thermophiles, lactobacillus casei, lactobacillus acidophilus, and bifidobacterium lactis	diabetics aged 35 to 65 with an FPG of 125 mg/dl, no insulin therapy, and a shorter than 20-year illness duration	Pregnancy, the use of nonsteroidal anti-inflammatory medicines, smoking, breastfeeding, vitamin and mineral use, and hormone replacement treatment, as well as common medical conditions such as thyroid, liver, gastrointestinal, cardiovascular, kidney, and autoimmune systems disease	In conclusion, this study demonstrated those type 2 diabetes patients who consume probiotic fermented milk containing L-acidophilus, L-casei, and B-lactis have lower insulin serum levels, higher Quicki, and only the decrease in HOMA-IR was statistically significant in the probiotic group and between the two groups. Homocysteine levels in the serum fell in both groups. After two months, there was no change in BMI in the probiotic consumption group
Anuradha 2017	India	/	100	Lactobacillus sporogenes, streptococcus faecalis, clostridium butyricum, and bacillus mesentericus + Metformin	Both sexes, between the ages of 40 and 65, newly diagnosed type 2 DM patients, with an 8-h FPG level between 126 and 140 mg/dl, and/or a 2-h PPG during an oral glucose tolerance test using 75g of glucose, between 200 and 240 mg/dl. HbA1c range of 6.5 to 7%	Patients taking anti-diabetic medications other than metformin have type 1 diabetes or type 2 diabetes. Patients with type 2 diabetes who also have another condition. Patients with type 2 DM who have complications from the disease. Gastroenteritis that persists. Patients taking loperamide, furosemide, corticosteroids, antacids, H2-receptor blockers, proton pump inhibitors, and cholestyramine. Such as cardiovascular disease, hepatic failure, and renal illness. Chronic drinking. Breastfeeding and pregnant ladies	Probiotics have not significantly increased the efficacy of combination therapy in any positive way. The probiotics trial group, however, was shown to have fewer reported gastrointestinal adverse effects linked with metformin medication, which was statistically significant. Therefore, a larger sample and a longer time frame can be used to further study the possible impact of probiotics in type 2 DM
Asemi 2013	Iran	–	54	Lactobacillus acidophilus, lactobacillus casei, lactobacillus bulgaricus, bifidobacterium breve, bifidobacterium longum, and streptococcus thermophilus	HbA1C 6.5%, FPG 126 mg/dl, and 2h PPG 200 mg/dl	Pregnant, taking insulin or vitamin supplements, or suffering from allergies, chronic kidney illness, liver disease, acute or chronic inflammatory disease of the lung, heart valve disease, or short bowel syndrome	In conclusion, 8 weeks of multispecies probiotic supplementation in diabetic individuals prevented an increase in FPG and led to a drop in serum CRP and an increase in plasma total GSH compared to placebo
Bahmani 2016	Iran	IRCT201311215623N13	81	Bread contained lactobacillus sporogenes	Patients with type 2 diabetes who have FPGs greater than 126 mg/dl, 2h PPGs greater than 200 mg/dl, and HbA1Cs greater than 6.5%. Men and women with T2DM between the ages of 35 and 70 were included in the current investigation	Gastrointestinal, renal, pulmonary, hepatic, or biliary disease; cancer; chronic use of probiotics, fiber laxatives, or stimulant laxatives; history of eating disorders and smoking; systemic antibodies, corticosteroids, androgens; insulin treatment; current participation in a clinical trial	As a result, type 2 diabetic individuals who consumed the synbiotic bread for eight weeks saw improvements in their plasma levels of NO and MDA; nevertheless, their levels of TAC, GSH, catalase, serum liver enzymes, calcium, iron, magnesium, and blood pressure were unaffected
Bayat 2016	Iran	IRCT2013041311763N7	80	Yogurt contained lactobacillus acidophilus La5, and bifidobacterium Bb12	Age requirements of 25 to 75 years, an FPG of at least 126 mg/dl, and controlled blood lipid levels without changing the drug’s instructions were required for eligibility. All participants were required to abstain from alcohol use, not smoke, and take metformin or glibenclamide to regulate their blood sugar	History of chronic conditions such pancreatitis, endocarditis, short bowel syndrome, renal, hepatic, lung, and heart disorders. None of the participants were breastfeeding mothers or pregnant women with autoimmune diseases. 80 Type 2 diabetic patients in total were chosen and asked to take part in the study	Our findings imply that C. ficifolia and probiotic yogurt, either separately or in combination, have positive effects on blood pressure, inflammation, and lipid profiles in Type 2 diabetes individuals. Our data show that yogurt and C. ficifolia taken together produce more notable results than either one taken separately. TG, HDL, TC, FPG, HbA1c, SBP, DBP, and CRP were among the variables that improved across the groups. Consuming C. ficifolia and probiotic yogurt appears to aid in the treatment of diabetic individuals. To clarify mechanisms and other effects, it appears that additional research in this field is required
Chaiyasut 2023	Thailand	(approval number: PPH No.1/2562)	40	Bifidobacterium breve	–	–	Twelve weeks of B. breve supplementation stopped the clinical parameters under study in Type 2 DM individuals from getting worse. The tiny sample size rendered the results seen in the placebo group inexplicable. The tiny sample size raised doubts about the treatment group’s changes in clinical metrics and microbiota. We firmly believe that additional thorough research is necessary to corroborate the findings of the current study, which could aid in the creation of probiotic-based supplements for the treatment of diabetes problems
Chen 2023	China	ChiCTR2100050108	48	Probiotic-X contained lactobacillus casei, lactobacillus plantarum, lactobacillus rhamnosus, bifidobacterium animalis, and bifidobacterium animalis	The patients were (i) aged 20 to 65 years, (ii) met the diagnostic criteria for type 2 diabetes in the Chinese guidelines for the prevention and treatment of type 2 DM, (iii) took metformin regularly or had taken other antidiabetic drugs but stopped taking them for more than 3 months, (iv) had had diabetes for 3 months, (v) had an HbA1c of 6.5%, and (vi) had not taken any antibiotics in the month prior to the trial	The patients (i) were hypertensive individuals whose blood pressure was still higher than 160/100 mm Hg despite regular treatment with two or more antihypertensive drugs, (ii) were pregnant or nursing mothers, (iii) had a history of long-term glucocorticoid use, (iv) were taking probiotic products one month before the start of the trial, and (v) failed to provide a complete sample of blood or stool	_
Daja 2019	Indonesia	–	60	Yogurt contained lactobacillus acidophilus La5, and bifidobacterium Bb12	Patients with Type 2 DM ranged in age from 30 to 60. If one of the following conditions was met, type 2 diabetes was verified as a diagnosis: FBG 126 mg/dl and history of type 2 diabetes with RBS 200 mg/dl. Metformin-taking participants	The following criteria were used to weed out subjects: BMI 30 kg/m^2^, use of Metformin in addition to antidiabetic medication, recent use of antibiotics, recent use of probiotic supplements, history of cardiovascular disease, chronic liver disease, chronic kidney disease, malignancy, chronic diarrhea, and chronic gastrointestinal diseases, as well as pregnancy or nursing	The impact of natural substances on diabetes has been extensively studied with a range of outcomes. This study may be the first to look at how weight, FBG, GLP-1, and calprotectin levels are affected by Job’s tears in Type 2 DM patients. In 216, it is concluded that adding Job’s tears to yogurt considerably improves the impact of yogurt in lowering FBG and reducing weight, but not in raising GLP-1 or lowering CP
Daja 2022	Indonesia	(23/02/KEP-FKUAJ/2019)	60	Yogurt contained lactobacillus acidophilus La5, and bifidobacterium Bb12	Patients with type 2 diabetes, ages 30 to 60, who are not using antidiabetic drugs or more than three tabs of metformin per day (to reduce bias from the effects of the maximum dose of three tabs of metformin per day) are eligible. Existing Type 2 DM, FPG 126 mg/dl, or RBS > 200 mg/dl detected in plasma were criteria for T2DM	Patients with a BMI of less than 30 kg/m^2^ (to maintain subject uniformity), those taking an antidiabetic medication other than Metformin, those who have recently used antibiotics, those who have recently taken probiotic supplements, those with a history of cardiovascular disease, chronic liver disease, chronic kidney disease, malignancy, chronic diarrhea, and chronic gastrointestinal disease, as well as those who are pregnant or nursing	This study looked at how adding Job’s tears, which contain FOS, to yogurt affected persons with Type 2 DM’s body size and glucose and lipid metabolism. Body size (weight, BMI), lipid metabolism (lipid profile), and CH metabolism (plasma glucose, plasma GA) characteristics were assessed. HDL increased significantly after Job’s tears were added to yogurt. However, a number of restrictions should be taken into consideration while interpreting the results
Ejtahed 2011	Iran	–	60	Yogurt contained lactobacillus acidophilus La5, and bifidobacterium Bb12	Every participant had had a type 2 diabetes diagnosis for at least a year	Smoking, having kidney, liver, or inflammatory bowel disease, thyroid disorders, immunodeficiency diseases, or lactose intolerance; requiring insulin injections; taking dietary supplements within the last 3 weeks or the 6-week study period; taking cholesterol-lowering medication, estrogen, progesterone, or diuretics; being pregnant or nursing; and consuming probiotic yogurt or any other probiotic products within the last 2 months	In conclusion, our study demonstrated that type 2 diabetics’ serum TC and LDL concentrations might be lowered by consuming probiotic yogurt containing L. acidophilus La5 and B. lactis Bb12. These results imply that probiotic yogurt may assist in lowering CVD risk factors in individuals with Type 2 DM
Ejtahed 2012	Iran	IRCT 138903223533N1	60	Yogurt contained lactobacillus acidophilus La5, and bifidobacterium Bb12	Every participant had had a type 2 diabetes diagnosis for at least a year	Smoking, having kidney, liver, or inflammatory bowel disease, thyroid disorders, immunodeficiency diseases, or lactose intolerance; requiring insulin injections; taking dietary supplements within the last 3 weeks or the 6-week study period; taking cholesterol-lowering medication, estrogen, progesterone, or diuretics; being pregnant or nursing; and consuming probiotic yogurt or any other probiotic products within the last 2 months	This study has shown that individuals with Type 2 DM who consumed 300 g/d of probiotic yogurt containing L. acidophilus La5 and B. lactis Bb12 had increased antioxidant status and fasting blood glucose levels. These results imply that probiotic yogurt is a useful food with potential anti-diabetic and antioxidant effects
Eliuz Tipici 2023	turkey	NCT05066152	34	Lactobacillus rhamnosus	38 Type 2 diabetic women, aged 30 to 60, were involved in the study. All of the volunteers were using oral diabetes medicines	People who use anti-epileptic medications, dietary supplements, incretin enhancers (dipeptidyl peptidase 4, DPP-4 inhibitors, or glucagon-like peptide 1 receptor agonists, GLP-1RAs), insulin, systemic antibiotics within 6 weeks, probiotic supplements within 3 months before inclusion, and women who are currently breastfeeding or pregnant. People with IBD, autoimmune diseases, or severe immunodeficiency states	In conclusion, the probiotic group in this study had considerably higher MUC2 and MUC3A expressions. This study’s key contribution is the positive effects of Lactobacillus GG on the expression of mucin genes involved in maintaining intestinal barrier functioning and weight reduction. The findings of our study need to be confirmed by interventions in which several probiotic strains are utilized in larger numbers of patients and followed for longer periods before being generalized, though
Feizollahzadeh 2017	Iran	IRCT201405265062N8	40	Soy milk contained lactobacillus plantarum A7	Type 2 diabetic patients aged 35–68 years	IBD, infection, liver illness, RA, smoking, drinking, recent antibiotic therapy, CRP positivity, and daily multivitamin and mineral consumption subjects	Overall, this study showed that patients with Type 2 DM who consumed probiotic soy milk and soy milk for eight weeks did not have a change in the serum concentration of Apn, CRP, or TNF-a. Probiotic soy milk and soy milk’s effects on the profile of adipokines and cytokines linked to metabolic diseases, particularly Type 2 DM, require further research. Probiotics appear to have a long way to go before they are fully integrated into the treatment of Type 2 DM patients. This emerging field of probiotic-based biological therapy may grow as a result of the isolation of some effective strains and the use of genetic engineering technologies to enhance their qualities
Firouz 2017	Malaysia	NCT01752803	101	Lactobacillus acidophilus, lactobacillus casei, lactobacillus lactis, bifidobacterium bifidum, bifidobacterium longum, and bifidobacterium infantis	Those who had Type 2 DM that had been present for at least 6 months before the start of the trial, were aged 30 to 70, were not taking insulin or antibiotics, had an HbA1c between 6.5% and 12%, and had an FBG of greater than 126 mg/dl	Having other chronic conditions, being pregnant, nursing, having severe diabetic problems (apart from hyperlipidemia and blood pressure), and so forth	Supplementing with probiotics was linked to improvements in HbA1c and fasting insulin. Probiotic supplementation significantly improves HbA1c and TG in normal-weight participants compared to OW/OB participants. The study’s multi-strain probiotics also demonstrated intestinal viability. Type 2 diabetics who were reasonably well controlled in this study should exercise caution if they are uncontrolled or taking insulin, as this group has just been specifically studied in recent studies. Future studies should concentrate on determining the function of probiotics and their intricate interactions with different organisms in the guts of normal-weight and OW/OB people. Additionally, the impact of this multi-strain probiotic supplement has to be studied in large cohorts, and potential mechanisms leading to its advantageous glycemic- or lipid-lowering benefits need to be identified
Hairi 2015	Iran	IRCT201405265062N8	40	Soy milk contained lactobacillus plantarum A7	Our study’s participants were Type 2 DM patients with FPG 126 mg/dl and 2h PPG 200 who were between the ages of 25 and 65 and had had the disease for at least a year	IBD, infection, liver illness, RA, smoking, drinking, recent antibiotic therapy, and daily multivitamin and mineral use in one’s medical history	Results show that diabetic individuals may benefit from consuming probiotic soy milk for lowering blood pressure because the intervention was done for diabetes patients of both sexes and ages. However, more research is required to discover how this probiotic strain affects anthropometric measurements
Hasanpour 2023	Iran	IRCT2015080423495N1	100	Lactobacillus rhamnosus, lactobacillus casei, lactobacillus bulgaricus, lactobacillus acidophilus, bifidobacterium breve, bifidobacterium longum, and streptococcus thermophilus + Soy milk contained lactobacillus plantarum A7; Lactobacillus rhamnosus, lactobacillus casei, lactobacillus bulgaricus, lactobacillus acidophilus, bifidobacterium breve, bifidobacterium longum, and streptococcus thermophilus; Soy milk contained lactobacillus plantarum A7	Patients with Type 2 DM aged 40 to 75 were enrolled in this study. Patients were deemed diabetic if their FPG level was 126 mg/dl or greater, or if they were using insulin injections or glucose-lowering medications	Participants who were lactating, undergoing hormone replacement treatment, consuming alcohol, or smoking cigarettes were prohibited from taking part. Furthermore, we excluded those who had breast cancer, were allergic to soy or cow’s milk, or had taken antibiotics three weeks prior to or during the trial period. Patients who changed the dosage of oral anti-diabetic medications, insulin therapy, or lipid-lowering medications, did not adhere to the advised diet, or consumed food or goods containing probiotic bacteria three weeks before or during the trial period were excluded from the investigation	According to our study, drinking soymilk along with a probiotic supplement for six weeks did not reduce cardiovascular risk factors compared to drinking regular milk, soymilk, and a probiotic supplement. However, compared to regular milk, the results demonstrated the beneficial effects of probiotic supplementation in decreasing SBP. As a result, our findings did not support any potential synergistic effects of soy products and probiotic bacteria in reducing cardiovascular risk factors in Type 2 DM patients. Because of this study’s limitations and the contradictory findings of the available data, research using
Hesih 2018	Taiwan	NCT02274272	68	Lactobacillus reuteri ADR-1; Lactobacillus reuteri ADR-3	Ages 25 to 70, BMI greater than 18.5 kg/m^2^, a diagnosis of type 2 diabetes more than six months ago, and a HbA1c score of 7% to 10%	Pregnancy; the existence of additional illnesses, such as malignancies (apart from benign tumors that are well-controlled), kidney failure or dialysis, heart disease, stroke, or autoimmune diseases; the presence of additional medical conditions that could compromise adherence to the protocol (such as malabsorption syndrome or the inability to take orally administered medications), participation in other clinical trials, the use of medications, including anti-diabetes drugs, antibiotics, or other probiotic products, three times the recommended dose	The outcomes of our double-blinded, randomized, placebo-controlled trial show that Type 2 DM patients benefit from the L. reuteri strains ADR-1 and ADR-3. The effects of consuming various L. reuteri strains may be influenced by changes in the intestinal flora. Thus, the level of L. reuteri in the feces following the consumption of ADR-1 or ADR-3 may be employed as a potential indicator of lower HbA1c levels in Type 2 DM patients
Hosseinzadeh_1 2013	Iran	IRCT138807062513N1	84	Yeast contained Saccharomyces cerevisiae	Age range of 35 to 55 years old, type 2 diabetes diagnosed by a doctor, and diabetes had existed for more than two years	Cardiovascular conditions, hepatitis, renal problems, gout, Parkinson’s, depression, use of monoamine oxidase inhibitors or steroidal anti-inflammatory medications, and insulin therapy	Patients with Type 2 diabetes who supplement with 1800 mg/day of brewer’s yeast in addition to standard therapies may have a moderate reduction in systolic and diastolic blood pressure
Hosseinzadeh_2 2013	Iran	IRCT138807062513N1	84	Yeast contained Saccharomyces cerevisiae	Aged 35–55 years with a history of at least 2 years of clinical type 2 DM diagnosis	History of gout, stroke, Parkinson’s disease, liver, renal, or intestinal issues; recent brewer’s yeast supplement use; ongoing antidepressant use; and insulin therapy	Brewer’s yeast supplementation had a negligible positive impact on glycemic indices in type 2 diabetes patients, according to the study’s findings. To determine the ideal brewer’s yeast dosage, more research is necessary. Additional research would shed light on any additional effects of brewer’s yeast in diabetes, such as oxidative stress markers
Hove 2015	Denmark	NCT00699426	41	Fermented milk contained lactobacillus helveticus	Having diabetes for more than a year, having an HbA1c between 6.0% and 10.0%, being between the ages of 40 and 70, and only taking oral glucose-lowering medications (metformin and sulfonylurea) as treatment	Severe organic or metabolic diseases, such as cancer, liver or severe kidney disease, severe heart failure, alcohol or drug addiction, severe neuropathy, anemia, insulin therapy, neutropenia, treatment with warfarin or other coumarin derivatives, or with medication for gastrointestinal diseases, pregnancy, or nursing. The use of antihypertensive and anti-diabetic drugs was permitted as long as neither the dosages nor the nature of the treatment altered throughout the research period	In conclusion, using AMBP as the primary objective, we were unable to demonstrate any BP-reducing impact of Cardi04 yogurt supplementation for 12 weeks in individuals with Type 2 DM. However, Cardi04 yogurt decreased both the daytime and 24-h HRs as well as fasting plasma glucose levels. Future research should examine the underlying mechanisms behind these effects to confirm and evaluate these findings in terms of their potential health-promoting impacts
Ismail 2021	Egypt	–	150	Yogurt contained Bifidobacterium animalis; Yeast contained Saccharomyces cerevisiae	Patients with Type 2 DM, aged 21 years or older, who consented to take part in this study can be either male or female	This study excluded people with thyroid issues, lactose intolerance, coronary heart disease, cancer in the past or present, malignancy, pregnancy, and lactation	In conclusion, we anticipate that our research will show that probiotics affect the gut microbiota in people with Type 2 diabetes and that, in theory, these changes will become metabolically beneficial with continuous probiotic usage. Our study’s findings indicated that giving patients with Type 2 DM probiotics daily for 16 weeks could enhance their FPG, PPG, HbA1c, and lipid profiles, and reduce their levels of inflammatory markers. As a result, probiotics have the potential to be a helpful adjuvant in this condition
Jiang 2021	China	ChiCTR2000038392	76	Bifidobacterium bifidum, lactobacillus acidophilus, and streptococcus thermophilus	The following were the inclusion requirements: Patients were between the ages of 18 and 75; they had type 2 diabetes and were diagnosed with diabetic nephropathy; they had not used any anti-diabetic medication in the three months before the trial; and their HbA1c levels were between 7 and 10%	The exclusion criteria were as follows: (1) patients with type 1 DM; (2) patients with hypoglycemic coma, diabetic ketoacidosis, hyperosmotic nonketotic coma or diabetes mellitus acute complications; (3) FPG was > 13.3 mmol/L; (4) total bilirubin was > 2.5 times of normal value; (5) serum creatinine was > 133 μmol/L in male patients, and > 124 μmol/L in female patients; (6) patients with history of hypertension, drug abuse, alcohol dependence or drug allergy; (7) patients’ intake of angiotensin-converting enzyme inhibitors or angiotensin receptor blockers within 3 months; and (8) patients’ intake probiotic and/or synbiotic supplements within 3 months before this study	As a result of the probiotic formula B. bifidum, L. acidophilus, and S. thermophilus being consumed, intestinal flora regulation, glycemic control, and mAlb/Cr were all improved in type 2 diabetic patients, according to our findings
Khalili 2019	Iran	IR.TBZMED.REC.1395.402	40	Lactobacillus casei	44 patients with Type 2 DM, 30 to 50 years old, and a BMI under 35 kg/m^2^ made up the volunteer group. For at least a year, Type 2 DM had been officially diagnosed in each patient	Smoking, the presence of kidney, liver, and/or IBD, thyroid disorders, immunodeficiency diseases, the need for insulin injections, the use of nutritional supplements within the three weeks before the test, the use of estrogen or progesterone, pregnancy or breastfeeding, taking any kind of antibiotics, and taking any other probiotic products within the two months before the test	This study reveals that L. casei could enhance SIRT1 and fetuin-A levels, as well as glycemic sensitivity. Management of their levels may be useful in the management of diabetes when considering the metabolic effects of SIRT1 and fetuin-A. The findings of the current study enable us to identify a novel mechanism of probiotic action in the management of metabolic problems associated with diabetes. Additionally, as demonstrated in this study, the good effects of probiotics on body weight may transfer into favorable metabolic effects and have a significant impact on glucose homeostasis
Kobyliak 2018	Ukraine	NCT03434860	53	Lactobacillus, lactococcus, bifidobacterium, propionibacterium, and acetobacter	Adult participants (ages 18 to 75, BMI greater than 25 kg/m^2^) who were diagnosed with Type 2 DM according to WHO (1999) for at least 6 months before the study, were treated with diet and exercise alone or metformin, SUs, and insulin on a stabilized dose for at least 3 months before the study, had HbA1c levels between 6.5% and 11.0%, and provided written informed consent	Type 1 diabetes mellitus (T1DM), use of anti-diabetic medications other than those listed in the inclusion criteria (such as pioglitazone, GLP-1 analogs, DPP IV inhibitors, etc.); regular use of a probiotic or prebiotic supplement within 3 months of enrollment; use of antibiotics within 3 months of enrollment; uncontrolled cardiovascular or respiratory disease; decompensated liver disease, including ascites, encephalopathy, or variceal bleeding	Patients with type 2 diabetes who received probiotic treatments saw a small improvement in insulin resistance. Application of probiotic agents and gut microbiota manipulation could be a new approach to managing hyperglycemia in Type 2 DM. To confirm any potentially advantageous association between the use of probiotics and modifiable cardiometabolic risk variables in type 2 diabetes patients, larger, well-designed, long-term RCTs are required
Kobyliak_1 2020	Ukraine	NCT04201938	54	Lactobacillus, lactococcus, bifidobacterium, propionibacterium, and acetobacter	Participants must be adult patients (aged 18 to 75) with a confirmed diagnosis of Type 2 DM (FPG 7.0 mmol/l; plasma glucose randomly measured 11.1 mmol/l; HbA1c 6.5% or glucose > 11.1 mmol/l 2 h after tolerance test with 75 g of glucose)	The main exclusion criteria were presence of Type 1 DM; intake of antidiabetic drugs except for those specified in the inclusion criteria (pioglitazone, glucagon-like peptide (GLP-1) analogs, dipeptide-peptidase 4 (DPP-4) inhibitors, etc.); regular intake of probiotics, prebiotics or antibiotics for 3 months prior the inclusion; previously diagnosed allergy to probiotics; gastrointestinal disorders including food allergy, gluten-sensitive enteropathy, ulcerative colitis; an uncontrolled cardiovascular or respiratory disease, an active malignant tumor or chronic infections; participation in another clinical trial; pregnancy or lactation	In conclusion, the current RCT showed that, compared to placebo, taking a multi-strain probiotic enriched with omega-3 PUFA supplement once daily for eight weeks in Type 2 DM patients significantly reduced IR, body weight, BMI, and markers of chronic systemic inflammation while also improving glycemic profile. Our data suggest the use of this combination as an intervention for decreasing the major risk factors in the complex course of Type 2 DM because probiotics and omega-3 supplementation both target similar molecular pathways, implicated in IR and chronic inflammation. Due to the potential summation of their individual beneficial effects, these findings point to a potential strategy in the manipulation of the gut microbiota using probiotics and various nutraceuticals, which may represent a more effective branch in the management of Type 2 DM
Kobyliak_2 2020	Ukraine	NCT04293731	55	Lactobacillus, bifidobacterium, lactococcus, propionibacterium, and acetobacter + Smectite gel	Participants must be adult patients (aged 18 to 75) with a confirmed diagnosis of Type 2 DM (FPG 7.0 mmol/l; plasma glucose randomly measured 11.1 mmol/l; HbA1c 6.5% or glucose > 11.1 mmol/l 2 h after tolerance test with 75 g of glucose)	The main exclusion criteria were presence of Type 1 DM; intake of antidiabetic drugs except for those specified in the inclusion criteria (pioglitazone, glucagon-like peptide (GLP-1) analogs, dipeptide-peptidase 4 (DPP-4) inhibitors, etc.); regular intake of probiotics, prebiotics or antibiotics for 3 months prior the inclusion; previously diagnosed allergy to probiotics; gastrointestinal disorders including food allergy, gluten-sensitive enteropathy, ulcerative colitis; an uncontrolled cardiovascular or respiratory disease, an active malignant tumor or chronic infections; participation in another clinical trial; pregnancy or lactation	In this RCT, we concluded that co-administration of probiotics with smectite to Type 2 DM patients can impact on synergistic enhancement of a single effect that manifested with significantly lower IR, waist circumference, markers of chronic systemic inflammation, and improvement in glycemic profile as compared to placebo
Król 2011	Poland	(Approval No. 386/2005)	20	Chromium + Yeast contained Saccharomyces cerevisiae	The study included individuals with type 2 diabetes who did not have any major side effects, such as retinopathy or nephropathy	Pregnancy or nursing, vitamin, and mineral supplements used within the last three months, thyroid hormone, estrogen, progesterone, and diuretic therapy, and alcohol and tobacco addiction	According to the study’s findings, type 2 diabetes individuals do not experience any changes in body mass, blood biochemistry, or mineral levels after receiving supplemental chromium in the form of Cr. enhanced brewer’s yeast (at dosages of 500 g/day for 8 weeks). This study also found that chromium supplementation has a weak antidiabetic potential
Kumar 2022	India	–	150	Capsule probiotics + Metformin	All patients diagnosed with Type 2 DM	Type 1 DM, pregnancy, taking additional anti-diabetic medications, and refusal to give informed permission are all prohibited	Probiotics added to metformin therapy for type 2 diabetes were found to lower HbA1c, FPG, and PPG levels compared to metformin alone. Probiotics have not demonstrated any meaningful results in combination therapy in terms of efficacy. The probiotics trial group, however, saw fewer gastrointestinal side effects attributed to metformin treatment
Lestari 2019	Indonesia	–	32	Yogurt contained lactobacillus acidophilus La5, and bifidobacterium Bb12	Diagnosed with type II diabetes between the ages of 30–65; FPG greater than 126 mg/dl; use of glucose-lowering medications like Glibenclamide or Metformin; Absence from statin or cholesterol-lowering medications; Absence from antibiotics during the study and for one month prior; possession of a refrigerator; ability to adhere to the treatment plan; and cooperation with the researchers	Having received a diagnosis of any difficulties (aside from hypertension and dyslipidemia), using any probiotic products for two weeks before the start of the trial, lactose intolerance, pregnancy, or nursing. Diarrhea, antibiotic use during the study, and any research-related problems were the drop-out criteria. Before the trial ever started	While probiotic yogurt could not considerably lower FPG, 100 ml/day of ordinary yogurt could significantly lower the FPG level. Both varieties of yogurt may raise HDL levels. As a result, they can be suggested as a functional diet for those with type 2 diabetes
Madempudi 2019	India	CTRI/2017/ 07/009164	79	Lactobacillus salivarius, lactobacillus casei, lactobacillus plantarum, lactobacillus acidophilus, bifidobacterium breve, and bifidobacterium coagulans	Type 2 DM (on stable metformin (500 mg) monotherapy for 8 weeks before the screening); BMI between 23 and 32 kg/m^2^; HbA1c between 7 and 9%; non-pregnant females; and those ready to sign an informed consent form before participating	Type 1 diabetes, a history of diabetic ketoacidosis, taking an antihyperglycemic medication other than metformin, fasting blood triglycerides > 400 mg/dl and/or LDL > 190 mg/dl, an HbA1c > 9%, known hypersensitivity to study drugs or constituents, a severe systemic disease, and use of ayurvedic, homeopathic, or herbal medications are all indications of diabetes type 1. Yogurt and other such dietary supplements were not allowed for the participants to consume during the study; the subjects’ normal eating habits and way of life were not altered	In conclusion, 12 weeks of UB0316 use in combination with metformin significantly reduced HbA1c and weight compared to placebo plus metformin. In Type 2 DM patients, there was a trend toward improvement in FBG, HOMA-IR, insulin, and lipid levels, but it was not statistically significant. To sum up, UB0316 is a probiotic multi-strain formulation that is well-tolerated and safe to take in conjunction with metformin, which is the first line of treatment for Type 2 DM
Mafi 2018	Iran	IRCT2017061134458N1	60	Lactobacillus acidophilus, bifidobacterium bifidum, lactobacillus reuteri, and lactobacillus fermentum	Patients with diabetic nephropathy have proteinuria and diabetic renal dysfunction, either with or without an increase in serum creatinine levels	Malignancy and/or liver cirrhosis, a history of active infection within the previous three months, the consumption of probiotic and/or synbiotic supplements during the previous three months, and a history of hospitalization within the previous three months	Overall, our research showed that probiotic supplementation for 12 weeks among DN patients improved markers of cardio-metabolic risk and glycemic control This shows that giving individuals with DN a probiotic supplement may have beneficial therapeutic potential
Mazloom 2013	Iran	_	34	Lactobacillus acidophilus, lactobacillus bulgaricus, lactobacillus bifidum, and lactobacillus casei	The trial was open to diabetic individuals with FPG 126 mg/dl, aged 25 to 65, and diagnosed with diabetes less than 15 years ago	Current smokers, people using multivitamin and NSAID medicine, people getting hormone replacement therapy, and anybody with any renal, liver, or pulmonary illness	For type 2 diabetes patients, a reduction in oxidative stress and cardiovascular risk factors appears to be the best course of action. The findings of this study showed that type 2 diabetic patients who received a probiotic oral medication for 6 weeks had lower levels of TG, MDA, and IL-6; however, the changes were not statistically significant. Future research to ascertain the probiotics’ therapeutic benefits on diabetic patients may be warranted in light of these findings
Mazruei 2018	Iran	IRCT201705035623N115	60	Honey contained bacillus coagulans	Participants aged 45 to 85 years old with DN and proteinuria > 0.3 g/24 h. We characterized DN as proteinuria diabetic renal disease, with or without an increase in serum creatinine levels	Malignancy and/or liver cirrhosis, a history of an active infection during the past three months, and the consumption of probiotic and/or synbiotic supplements within the past three months	Overall, results from this study showed that probiotic honey consumption for 12 weeks among DN patients improved insulin metabolism, total-/HDL cholesterol, serum CRP levels, and plasma MDA levels but did not impact other metabolic profiles
Miraghajani 2017	Iran	NCT02704884	40	Soy milk contained lactobacillus plantarum A7	Patients who were diagnosed with stages 1 or 2 of nephropathy, which is defined as satisfying criteria such as FPG > 126 mg/dl, hypoglycemic medications, or insulin consumption, and proteinuria > 300 mg/day, were eligible for enrolment in this study	Major exclusion criteria included changing medication dosages, soymilk allergies, intolerances, or avoidance, smoking, alcoholism, recent antibiotic therapy, use of vitamin and mineral supplements, and any medical conditions like IBD, infection, liver disease, or RA	In conclusion, our analyses show that consuming fortified soy milk with L. plantarum A7 for eight weeks has a lot of positive health effects for people with DN and is a promising method for enhancing renal function
Mirjalili 2023	Iran	IRCT20220226054125N1	72	Yogurt contained lactobacillus acidophilus La5, and bifidobacterium Bb12	Patients with Type 2 DM, active medical records, a final test with LDL equal to or greater than 100 mg/dl, and a minimum age of 30 years were required for inclusion	Diseases of the liver or kidneys, digestive tract problems, heart conditions, untreated hypothyroidism, pregnancy, lactation, lactose intolerance, insulin injection, use of estrogen, progesterone, corticosteroids, and cholesterol-lowering medications, consumption of vitamin, mineral, and omega-3 supplements for three weeks before the start of the study and throughout the study, and use of antibiotics for one month prior to the start of the study and throughout the study	In conclusion, we discovered that consuming 200 g of probiotic yogurt daily for 12 weeks had some positive impacts on dyslipidemia and glycemic status. To draw more accurate conclusions, additional RCTs examining the duration of Type 2 DM and the condition of malnutrition are required
Mirmiranpour 2019	Iran	IRCT20170305032883N2	115	Lactobacillus acidophilus	Type 2 diabetes, not using insulin, age 40 to 60, FPG values between 125 and 250 mg/dl, and a HbA1c of 7–8% were the inclusion criteria	Pregnancy, breastfeeding, taking certain medications (effective on blood glucose, such as corticosteroids, diuretics, and antibiotics), using vitamin supplements and medicinal plants, changing anti-diabetic medications within the previous four months, a history of lactose intolerance or allergies, chronic and uncontrolled hepatitis, renal, psychological, or cardiac disease, and drug or alcohol addiction were among the exclusion criteria. Additionally, those in the study who followed a strict diet or engaged in vigorous exercise were excluded	The current findings show that type 2 diabetics who take probiotic supplements (individually or in combination with cinnamon) have lower blood glucose levels and higher levels of antioxidant enzymes. The findings of this study show that probiotics, cinnamon, and synbiotics (a probiotic and cinnamon combination) can all lower fasting blood glucose and HbA1c levels in diabetic individuals without significantly outperforming the other treatments. All cinnamon, synbiotic, and probiotic groups experienced a modest rise in the level of antioxidant enzymes, with the probiotic group experiencing the largest change. Probiotics improved type 2 diabetic patients’ blood glucose profiles and antioxidant enzymes more effectively, according to all studies
Miryousefiata 2021	Iran	–	60	Lactobacillus rhamnosus, lactobacillus casei, lactobacillus bulgaricus, lactobacillus acidophilus, bifidobacterium breve, bifidobacterium longum, and streptococcus thermophilus	Patients with non-insulin-dependent diabetes (NIDDM), which is commonly caused by obesity and inactivity, whose blood sugar is assessed by the WHO or ADA index, and less They will be between the ages of 25 and 65 when they develop diabetes, which takes 15 years. Selected participants can continue taking their regular dosages of these medications throughout the trial as long as they are at a controlled blood sugar and cholesterol level and taking their prescribed medications as usual	Vitamin supplements and hormone replacement therapy shouldn’t be used by certain people. Additionally, persons who smoke, drink alcohol, have chronic kidney, liver, or lung problems, as well as those who have allergies, heart valve disease, short bowel syndrome, or chronic or acute inflammatory diseases (particularly acute pancreatitis and endocarditis). Women who were pregnant or nursing, as well as those with autoimmune conditions, were taken off the list of those who could participate	Overall, the results of this study showed that giving type 2 diabetics a dietary supplement containing a pure blend of probiotics for six weeks can lower their fasting blood sugar levels and considerably raise their HDL cholesterol levels. Increase to prevent changes in other lipid markers, particularly total cholesterol in individuals. However, it can be argued that both of these effects may stop the development of diabetes and its side effects, such as insulin resistance and hyperglycemia, or stop the development of other comorbidities, such as cardiovascular disease, hyperlipidemia, heart attack, and stroke
Mobini 2017	Sweden and Denmark	NCT01836796	44	Lactobacillus reuteri low dose; Lactobacillus reuteri high dose	Type 2 diabetes diagnosis > 6 months prior, 50–75 years of age, abdominal obesity (women: waist > 80 cm; men: waist > 94 cm), HbA1c 6.7–10.4% (50–90 mmol/mol), requirement that insulin be used in anti-hyperglycemic therapy, and declared availability throughout the trial are all requirements. 7) stable weight (5 kg) and HbA1c (5 mmol/mol) for 6 months; 8) BMI 25–45 kg/m^2^; 9) fasting C-peptide > 0.27 nmol/L; 10) signed informed consent	Smokers who smoke a lot, type 1 diabetes, and ischemic heart disease with an incident	L. reuteri DSM 17938 administration for 12 weeks had no impact on HbA1c in type 2 diabetics receiving insulin therapy. However, L. reuteri increased insulin sensitivity in a small fraction of individuals, and we suggest that this may be related to the high diversity of the gut microbiota at baseline
Mohamadshahi_1 2014	Iran	–	42	Yogurt contained lactobacillus acidophilus La5, and bifidobacterium Bb12	Type 2 DM patients. All subjects were overweight or obese (BMI ≥ 25)	Injections of insulin, medication changes, corticosteroid use, immunosuppressive and NSAID use, smoking, lactose intolerance, thyroid dysfunction, chronic inflammatory diseases, cardiovascular disease, renal dysfunction, pregnancy, breast-feeding, and following any diet or exercise plans that involve weight loss or gain are all examples of risk factors. Additionally, subjects who had consumed probiotic yogurt or nutritional supplements within two months of the test were disqualified	The results showed that probiotic yogurt consumption can lower HbA1c and a few inflammatory markers in people with type 2 diabetes mellitus. Thus, it is suggested that consuming probiotic yogurt may be utilized as a substitute for traditional preventive and therapeutic measures to manage diabetic problems. To validate these effects, additional research using a bigger sample size, a longer intervention time, and different probiotic strains is required
Mohamadshahi_2 2014	Iran	IRCT2014021516588N1	42	Yogurt contained lactobacillus acidophilus La5, and bifidobacterium Bb12	BMI > 25, 100 mg/dl serum LDL (normal range for men and women) in both sexes	Injections of insulin, any modifications to medication use, smoking, lactose intolerance, thyroid dysfunction, chronic inflammatory disorders, cardiovascular disease, renal dysfunction, pregnancy, breastfeeding, and engaging in any weight-loss or weight-gain regimens are all examples of risk factors	According to this study, probiotic yogurt can help Type 2 DM patients with their lipid problems. Thus, it is suggested that consuming probiotic yogurt may be utilized as a substitute for traditional preventative and therapeutic measures to lessen diabetic problems. We also recommend conducting additional research with a larger sample size, a longer intervention period, and other probiotic types
Ostadrahimi 2015	Iran	IRCT201307092017N14	60	Fermented milk contained lactobacillus acidophilus and bifidobacterium	Diabetic people with an FPG of less than 125 mg/dl, age range of 35 to 65, and no insulin medication	Pregnancy, breastfeeding, patients with common medical conditions like thyroid, liver, gastrointestinal, cardiovascular, kidney, and immune system defects, vitamin and mineral use, and NSAID users are some examples of common health risks	Diabetes patients who drank probiotic-fermented milk (kefir) instead of regular fermented milk experienced lower fasting blood glucose and HbA1C values. The serum level of total cholesterol decreased when compared to the other two groups, however this decrease was not statistically significant. These results imply that medical nutrition management of diabetes patients may benefit from probiotic fermented milk
Palacious 2020	Australia	ACTRN12613001378718	60	Lactobacillus plantarum, lactobacillus bulgaricus, lactobacillus gasseri, bifidobacterium breve, bifidobacterium animalis, bifidobacterium bifidum, streptococcus thermophilus, saccharomyces boulardii	Aged 18 years; BMI 25 kg/m^2^; diagnosed with prediabetes or Type 2 DM within the past 12 months in accordance with American Diabetes Association standards. Treated with diet alone or diet plus metformin; undiagnosed gastrointestinal disorders; not currently taking any anti-obesity medications or blood glucose-lowering medications (other than metformin); abstaining from taking any antibiotics or dietary supplements, including probiotics, for four weeks before the start of the study; and willing to follow the study protocol for the duration of the study	Not meeting inclusion criteria	In this pilot trial, it was established that administering the multi-strain probiotic with or without metformin was safe and well tolerated. The participants taking metformin and the probiotic together, with advantageous changes in SCFA-producing bacteria, were found to have significant improvements in fasting plasma glucose, insulin resistance, and the permeability marker zonula. However, no differences in metabolic, inflammatory, or permeability markers were found between the probiotic and placebo groups. Probiotics may improve the effectiveness of metformin and control butyrate production in people with prediabetes and recently diagnosed Type 2 DM, according to preliminary research from this study. The research findings help to partially explain the potential processes by which probiotics might complement metformin in the treatment of people with high blood sugar levels
Perraudeau 2020	Multicenter	NCT03893422	58	Probiotic formulation contained three (WBF-010); Probiotic formulation contained five (WBF-011)	Adults with Type 2 DM, defined as FPG 126 mg/dl or HbA1c of 6.8%, treated with diet and exercise alone, or combined with metformin with or without a sulfonylurea were eligible	If a subject had taken an antibiotic, antifungal, antiparasitic, or antiviral medication within 30 days of enrolling in the trial, they were disqualified from participation	Summary: In subjects with Type 2 DM who are primarily receiving metformin monotherapy, this double-blind, randomized, placebo-controlled study provides an initial demonstration of a novel 5-strain probiotic formulation, WBF-011, that significantly improved total glucose AUC0-180 min relative to placebo. Important secondary endpoints for glucose control, like HbA1c, also showed signs of improvement. No changes in body weight, HOMA-IR, or fasting glucose levels were found, indicating that the majority of the benefit is likely due to a drop in plasma glucose levels during the postprandial period. As far as we are aware, this is the first randomized controlled experiment to give four of the five species to Type 2 DM-affected human patients. Future research aimed at confirming and extending these findings is justified by these findings
Raygan 2019	Iran	IRCT20170513033941N28	54	Selenium yeast + Lactobacillus acidophilus, lactobacillus reuteri, lactobacillus fermentum, and bifidobacterium bifidum	Participants in the study, who ranged in age from 45 to 85, had CHD and Type 2 DM diagnoses. Type 2 DM and 2- and 3-vessel CHD, respectively, were diagnosed using the American Heart Association and American Diabetes Association criteria	Patients with thyroid conditions, severe renal insufficiency, hepatic failure, acute myocardial infarction, and cardiac surgery within the previous three months, as well as those who had consumed probiotics and/or synbiotics during that time frame, were excluded from the study	Overall, co-supplementing probiotics and selenium with diabetic patients with CHD had positive impacts on metabolic profiles and indices of mental health
Raygan_1 2018	Iran	IRCT2017082733941N5	60	Lactobacillus acidophilus, lactobacillus casei, and bifidobacterium bifidum	Patients with Type 2 DM who have 2- and 3-vessel CHD and are 40–85 years old. Based on the American Diabetes Association’s criteria, type 2 DM was identified. Furthermore, the American Heart Association guidelines were followed while making the CHD diagnosis	Probiotic and/or synbiotic use within the previous three months of the intervention, prebiotic use, antioxidant and/or anti-inflammatory supplements like vitamin E, vitamin C, and omega-3 fatty acids, antibiotic use, acute myocardial infarction, recent cardiac surgery, renal or hepatic failure, and antibiotic use were all considered exclusion criteria	Overall, probiotic supplementation for 12 weeks demonstrated positive effects on diabetic patients with CHD’s glycemic control, HDL cholesterol, total-/HDL cholesterol ratio, biomarkers of infection, and oxidative stress. Our findings show that taking probiotic supplements may have a positive therapeutic effect
Raygan_2 2018	Iran	IRCT2017073033941N4	60	Vitamin D3 + Lactobacillus acidophilus, bifidobacterium bifidum, lactobacillus reuteri, and lactobacillus fermentum	People diagnosed with Type 2 DM using the American Diabetes Association’s (2014) criteria and CHD diagnosed using the American Heart Association’s guidelines with 2- and 3-vessel CHD were included	Patients with thyroid conditions and those taking vitamin D, probiotics, and/or synbiotics during the previous three months were excluded from this study	Overall, diabetic patients with CHD who received vitamin D and probiotic co-supplementation after 12 weeks reported improved mental health metrics, glycemic control, HDL-cholesterol levels, CRP, NO, and TAC, but not other metabolic profiles and BP
Razmpoosh 2019	Iran	RCT2013100714925N1	60	Lactobacillus rhamnosus, lactobacillus casei, lactobacillus bulgaricus, lactobacillus acidophilus, bifidobacterium breve, bifidobacterium longum, and streptococcus thermophilus	People with type 2 diabetes who met the American Diabetes Association’s criteria for at least 10 months before the study’s start, were between the ages of 30 and 75, were free of antibiotics and hormone replacement therapy such as insulin, and had controlled glucose and lipid profile levels. Additionally, taking common prescription medications as long as the dosage remained the same throughout the study was permitted	The presence of liver, kidney, inflammatory, or immune deficiency diseases; thyroid disorders or lactose intolerance; the need for insulin therapy; the use of any types of estrogen, progesterone, cholesterol-lowering drugs or diuretics; pregnancy or breastfeeding; and the consumption of any types of probiotics are also considered to be exclusion criteria. Current smokers, and people taking non-steroidal anti-inflammatory drugs, multivitamins, or nutritional supplements within the past three weeks before the study’s	According to our research, taking a probiotic supplement containing seven different bacterial strains for six weeks significantly affected the levels of FPG and HDL-C in patients with type 2 diabetes compared to baseline levels. The effects of probiotics on insulin levels and insulin resistance in the intervention group were comparable to those of the placebo group, although the probiotic group’s fasting plasma insulin was higher in the within-group comparison. To the best of our knowledge, this is the first study to document the decreasing effects of probiotic supplements on FPG levels. As a result, given that the prevalence of diabetes mellitus and its co-morbidities are rising alarmingly, concentrating on these positive natural variables in addition to pharmacological therapy may hold hope for reducing the onset of non-communicable diseases. However, additional research is required to validate our findings and identify potential underlying mechanisms
Rezaei 2017	Iran	IRCT201404185866N18	90	Yogurt contained lactobacillus acidophilus La5, and bifidobacterium Bb12	having had a type 2 diabetes diagnosis for more than a year; 2) ability to adhere to the treatment plan and work with the researchers; 3) obtaining insulin; 4) living in Gorgan; 5) having LDL of greater than 100 Mg/dl, and 6) being between the ages of 30 and 70	Death, heart, kidney, hepatic, pulmonary, and inflammatory diseases, chronic gastrointestinal illnesses, low thyroid function, lactose intolerance, insulin injection, use of estrogen, progesterone, corticosteroids, cholesterol-lowering, and diuretic medications, a body mass index (BMI) of greater than 35, smoking, breastfeeding, pregnancy, use of vitamin, mineral, and omega-3 supplements three weeks before the start of the study, and co-morbid conditions are excluded. Beginning with the study	In this study, daily consumption of probiotic yogurt for four weeks had positive effects on blood glucose, glycated hemoglobin, diastolic blood pressure, and serum lipid levels in the intervention group, but had no discernible impact on cholesterol, HDL, and CRP when compared to the control group. The authors advise additional research with longer intervention times to reach a conclusive result in this area. In general, type II diabetes individuals can benefit from consuming probiotic yogurt as an additional therapy
Rustani 2022	Indonesia	–	36	Lactobacillus plantarum	The following were the requirements for Type 2 DM patients to be included in the study: age between 20 and 50, BMI 30, HbA1c 6.5%, not being pregnant or nursing, not being in menopause, not smoking, not drinking alcohol, not taking antidiabetic medications, and not taking any other medications	Being pregnant, receiving probiotic and/or antibiotic therapy during treatment, or withdrawing permission during the study were exclusion factors	In conclusion, the HbA1c level in the probiotic group was improved after an 11-week intervention with L. plantarum Dad-13 powder. Some beneficial bacteria that increase metabolites, such as SCFA alignment with the growth in total SCFA, acetic, propionic, and butyric acids, may be modulated by probiotics
Sabico 2017	Kingdom of Saudi Arabia	NCT01765517	78	Bifidobacterium bifidum W23, bifidobacterium lactis W52, lactobacillus acidophilus W37, lactobacillus brevis W63, lactobacillus casei W56, lactobacillus salivarius W24, lactococcus lactis W19, and lactococcus lactis W58	At first, Saudi adults (30–60 years of age) with newly diagnosed Type 2 DM were invited to take part	Participants with unstable glycemic control and Type 2 DM comorbidities were removed. Participants who would anticipate changes in antidiabetic medications (if any) in the next 6 months, women lactating or pregnant, those taking insulin or its analogues, and those with gastrointestinal diseases, such as irritable bowel syndrome, were excluded from the study	In conclusion, a 12-week multi-strain probiotic supplementation in Type 2 DM patients who had not taken any medication led to no appreciable changes in the levels of circulating endotoxins but was beneficial in terms of better HOMA-IR and a slight decrease in abdominal adiposity. If probiotic supplementation can prevent diabetes complications, a bigger cohort and a longer course of treatment may be required
Sabico 2019	Kingdom of Saudi Arabia	NCT01765517	78	Bifidobacterium bifidum W23, bifidobacterium lactis W52, lactobacillus acidophilus W37, lactobacillus brevis W63, lactobacillus casei W56, lactobacillus salivarius W24, lactococcus lactis W19, and lactococcus lactis W58	Adult Saudi participants, aged 30 to 60, with a recent Type 2 DM diagnosis (6 months)	Participants were clients of the King Salman Hospital’s outpatient division in Riyadh, Saudi Arabia. Patients with poor glycemic control (HbA1c > 7%) and diabetes sequelae (retinopathy, neuropathy, nephropathy, etc.) as documented in their medical records were excluded. Pregnant or lactating women, those on insulin or its analogs, people taking probiotics or antibiotics six weeks before enrollment, people taking these medications, and people with gastrointestinal problems were all excluded from the study	In conclusion, regular supplementation with a variety of probiotic strains for six months can dramatically lower endotoxin and inflammatory adipokine levels, as well as HOMA-IR, in Arab Type 2 DM patients. Despite the small sample size and meticulous analysis, there was a significant reduction in insulin resistance in favor of the probiotics group. This improvement deserves clinical attention. The study’s results provide significant information that will deepen our understanding of how multi-strain probiotic supplements benefit diabetics who come from relatively homogeneous and understudied ethnic groups. The results also highlight the difficulties in conducting randomized clinical trials in this region of the world, where high-quality evidence-based research is still being developed and would benefit from more public participation. However, this study advises using multiple-strain probiotics as an additional treatment for those with Type 2 DM
Sahin 2022	Turkey	–	312	Bifidobacterium animalis subsp. lactis BB-12 + Metformin	The diagnosis of type 2 diabetes was based on the presence of at least one patient who fulfilled the requirements for the condition: FPG 126 mg/dl, 2h PPG 200 mg/dl in the OGTT, HbA1c levels 6.5%, and blood glucose levels 200 mg/dl at any time when there were characteristic hyperglycemia-related signs and symptoms. When at least one of the following criteria was satisfied, prediabetes was diagnosed: impaired glucose tolerance, impaired fasting blood glucose, and HbA1c values between 5.7% and 6.4%	The study excluded IBD patients, those who had previously used metformin, people with persistent diarrhea or dyspepsia, and people with diabetic autonomous neuropathy	In conclusion, metformin therapy is the most important part of treating Type 2 DM, and non-compliance due to gastrointestinal side effects is our largest difficulty. In our experiment, supplementing metformin therapy with probiotic support decreased complaints of diarrhea and bloating and increased patient adherence to the regimen. Patients receiving probiotic supplementation demonstrated improved glycemic control and a decline in HbA1c, possibly due to higher treatment adherence and the potential effects of probiotics on the intestinal-pancreatic axis. In conclusion, there were fewer negative side effects and improved treatment adherence and HbA1c reduction in patients who added probiotic support to their metformin medication. It is vital to challenge our findings in related research and support them with meta-analyses when discussing the inclusion of probiotic aid to metformin treatment for clinical objectives
Sato 2017	Japan	UMIN000018246	68	Fermented milk contained lactobacillus casei	Patients with stable glycemic control who have type 2 DM	Because their prescriptions can change during the study period, patients with HbA1c8.0% were disqualified from participation. If any of the following conditions were identified upon registration, the study’s chosen subjects were disqualified from participation: 1) significant liver illness excluding fatty liver, 2) IBD, and 3) significant kidney disease (serum creatinine level 2.0 mg/dl and/or hemodialysis)	Our findings concluded that probiotic treatment decreased bacterial translocation and partially altered the gut microbiota in Japanese patients with type 2 diabetes. However, additional effective treatments, such as the application of specific synbiotics and/or more probiotic cells with a longer administration duration, may be required to further improve gut dysbiosis and minimize chronic inflammation
Shakeri 2023	Iran	IRCT201311215623N13	78	Bread contained lactobacillus sporogenes	Patients with type 2 diabetes have an FPG of more than 126 mg/dl, a 2h PPG of more than 200 mg/dl, and a HbA1C of more than 6.5%	Subjects with chronic kidney, liver, lung, and chronic or acute inflammatory diseases, heart valve disease, short bowel syndrome, allergies, or those who used insulin or vitamin supplements were excluded from the study	In summary, compared to probiotic and control bread, consumption of the synbiotic bread for 8 weeks among patients with Type 2 DM significantly decreased serum TAG, VLDL-C, TC/HDL-C and significantly increased serum HDL-C levels, but had no effect on FPG, TC, LDL-C, and non-HDL-C levels. Determining the mechanisms causing these impacts will require more research
Soleimani 2017	Iran	IRCT201601025623N69	60	Lactobacillus acidophilus, lactobacillus casei, and bifidobacterium bifidum	Patients who were diabetic and receiving regular HD for at least a year ranged in age from 18 to 80	Exclusion criteria for the study included those who were pregnant, had intestinal conditions, took probiotic supplements or other forms of probiotics (such as probiotic yogurt, kefir, and other fermented foods), took prebiotic, antioxidant, and/or anti-inflammatory supplements (such as vitamin E, vitamin C, and omega-3 fatty acids), and had taken antibiotics or immunosuppressive drugs within three months of enrolling in the study	Overall, our research showed that probiotic supplementation for 12 weeks among diabetic HD patients had positive impacts on glucose homeostasis metrics and a few biomarkers of oxidative stress and inflammation. The variations in HOMA-B and TAC levels were not substantially different across the groups when we corrected the analyses for age, baseline BMI, and baseline values of biochemical variables
Tajadadi-Ebrahimi 2014	Iran	IRCT201311215623N13	81	Bread contained lactobacillus sporogenes	We included those with Type 2 diabetes who were 35 to 70 years old	We disqualified anyone who was expecting, taking insulin or vitamin supplements, had chronic kidney, liver, or lung disease, as well as those who had acute or chronic inflammatory conditions, heart valve disease, short bowel syndrome, and allergies	The synbiotic bread exhibited positive effects on insulin metabolism in Type 2 DM patients for 8 weeks but did not affect FPG, QUICKI, or serum CRP levels
Toejing 2021	Thailand	–	36	Lactobacillus paracasei HII01	According to WHO guidelines, having type 2 diabetes, being between the ages of 20 and 70, and starting the study without receiving any antibiotic therapy for 14 days to avoid the bactericidal effect	The exclusion criteria included having an abnormal liver or renal function test, having a history of malignant, micro-, and macrovascular complications, being chronically intoxicated or using alcohol heavily (defined as binge drinking five or more days in the previous month), being pregnant or nursing, taking nonsteroidal anti-inflammatory drugs, heavy cigarette smoking or heavy smoker (defined as more than 20 cigarettes per day), and receiving hormone replacement therapy. Additionally, those who changed their medication, or lifestyle, or began taking antibiotics at any point during the examination were disqualified from the study	In conclusion, the results of this trial show that L. paracasei HII01 supplementation has positive effects on glycemic control and other metabolic variables by improving gut flora and subsequently alleviating endotoxemia, which raises the possibility that this probiotic could be used as an adjunctive therapy in people with Type 2 diabetes
Tonucci 2017	Brazil	RBR19644	45	Fermented milk contained lactobacillus acidophilus and bifidobacterium animalis subsp. lactic BB-12	Recruited from two endocrinology clinics, age 35 to 60 years, BMI less than 35 kg/m^2^, and type 2 diabetes diagnosed for at least one year	Participants who had a history of cancer or cardiovascular disease and were currently taking insulin, a DPP-4 inhibitor, or a GLP69 1 analogue, or who had clinical indications of chronic sickness or gastrointestinal issues were excluded from the study. Smoking, using probiotics, vitamins, anti-obesity medications, anti-inflammatory meds, and antibiotics within three months after enlistment, as well as being pregnant or nursing are prohibited	The results of this study reveal that consuming probiotics helps Type 2 DM patients maintain better glycemic control, however, consumption of fermented goat milk appears to be associated with changes in inflammatory cytokines (TNF- and resistin) and acetic acid concentrations
Zhang 2020	China	NCT02861261	409	Probiotics + BBR; Probiotics	Based on criteria established by the World Health Organization in 1999, newly identified Type 2 DM. Both sexes are qualified. 2. Between 20 and 70 years old. 3. BMI values of 19.0 and 35.0 kg/m^2^. 4. Fully comprehend the research. 5. Provide written, conscious consent. 6. Are drug-naive (have only received treatment for hyperglycemia by healthy lifestyle adjustments, such as oral diabetes medications, GLP-1 agonists, or insulin). 7. Before screening, engage in lifestyle modifications (diet and exercise) for glycemic management for at least two months. 8. At screening, HbA1c was between 6.5% and 10.0%, and FPG was between 7.0 and 13.3 mmol L1	Serum alanine aminotransferase concentrations that are more than 2.5 times over the upper limit of the normal range are considered to have severe liver impairment. Impaired renal function (defined as serum creatinine > 132 mol L-1 or estimated glomerular filtration rate (eGFR) 60 ml (min 1.73 m^2^)) as well as psychiatric illness, severe infection, severe anemia, and neutropenia. 2. Severe organic heart illnesses, such as hypertrophic or dilated cardiomyopathy, congenital heart disease, and rheumatic heart disease. III is the heart function New York Heart Association class grade. 3. Sensitivity to gentamycin or other antibiotics with aminoglycosides	Since BBR has an anti-diabetic effect on Type 2 DM, our study reports a human microbial-linked mechanism behind this effect

FPG, Fasting plasma glucose; HbA1c, Hemoglobin A1c; TGA, Triglycerides; LDL, Low-density lipoprotein; HDL, High-density lipoprotein; DM, Diabetes mellitus; L, Lactobacillus; B, Bifidobacterium; BBR, Berberine; TC, Total cholesterol; VLDL, Very low-density lipoprotein; IBD, Inflammatory bowel disease; RA, Rheumatoid arthritis; CHD, Congenital heart disease; BMI, Body mass index

**Table 2:** Baseline characteristics

ID	Age (years), Mean	Sex (M: F, n	Weight (Kg), Mean	Duration of the disease (Years), Mean	FPG (mg/dl), Mean	HbA1c (%), Mean	Total cholesterol (mg/dl), Mean	LDL (mg/dl), Mean	HDL (mg/dl), Mean	TGA (mg/dl), Mean
Abbasi 2018	55.25	–	71.2	–	–	–	190.45	114.95	47.45	175.45
Alihosseini 2017	–	34:26	–	6.915	–	–	–	–	–	–
Anuradha 2017	52.09	55:45	–	–	133.65	6.72	163.5	105.95	45.38	168.28
Asemi 2013	51.55	–	73.26	–	139.15	7.03	167.95	83.8	54.8	146.75
Bahmani 2016	52.23	–	77.5	–	–	–	–	–	–	–
Bayat 2016	51.36	28:52	75.06	–	157.76	7.445	186.4	112.12	41.875	190.9
Chaiyasut 2023	62.48	6:34	57	–	145.33	7.145	194.63	108.28	60.8	157.53
Chen 2023	47.8	29:19	91.9	9.55	–	–	–	–	–	–
Daja 2019	44.15	27:33	64.65	–	129.65	–	–	–	–	–
Daja 2022	44.55	27:33	65.3	–	–	–	181	134.95	45	126
Ejtahed 2011	50.93	23:37	75.8	4.95	138.69	7.08	195	116.78	49.5	143
Ejtahed 2012	50.93	23:37	75.8	4.95	138.69	7.08	–	–	–	–
Eliuz Tipici 2023	45.25	–	87.65	5	145	7.56	–	145.26	47.85	147.58
Feizollahzadeh 2017	55.25	19:21	71.23	7.8	134.5	–	–	168.5	45	279
Firouz 2017	53.55	71:65	75.6	–	144.9	7.615	184.65	1.08	50.27	129.65
Hairi 2015	55.25	19:21	71.23	7.8	–	–	–	–	–	–
Hasanpour 2023	53	73:27	74.18	–	161	–	171.42	82.28	44.8	145.5
Hesih 2018	–	–	–	–	–	–	–	–	–	–
Hosseinzadeh_1 2013	46.25	21:63	75.33	–	–	–	192.75	107.5	30.95	179.15
Hosseinzadeh_2 2013	46.2	21:63	–	–	164.55	9.05	–	–	–	–
Hove 2015	59.55	–	89.2	–	153.9	7.05	143.08	67.6725	42.54	123.9
Ismail 2021	47.76	–	85.83	–	137.7	8.13	271.43	118.16	44.03	174.16
Jiang 2021	56.04	27:49	–	–	184.59	8.22	–	–	–	–
Khalili 2019	44.47	14:26	80.3	0.32	156.8	7.066	–	–	–	–
Kobyliak 2018	54.7	–	98.14	6.035	160.92	8.36	–	–	–	–
Kobyliak_1 2020	56.01	–	101.27	10.62	178.83	8.43	–	–	–	–
Kobyliak_2 2020	55.37	–	93	9.73	174.51	8.3	–	–	–	–
Król 2011	54.7	11:9	–	11.5	181.8	7.7	228.54	132.25	55.3	212.84
Kumar 2022	50.98	84:66	–	–	133.65	6.74	163.51	106	45.4	168.26
Lestari 2019	54.5	10:22	66	3.25	146.25	–	160.32	129	37.18	138.75
Madempudi 2019	52.4	62:17	68.6	–	149.2	8.1	174.5	92.8	49.4	160.6
Mafi 2018	59.9	–	69.9	–	139.05	6.9	153.5	82.3	31.8	196.8
Mazloom 2013	53.6	8:26	71.56	–	154.195	–	179.14	97	41.14	180.42
Mazruei 2018	61.5	–	78.8	–	127.75	–	162.2	91.2	44.75	131.1
Miraghajani 2017	55.25	–	71.23	7.8	–	–	–	–	–	–
Mirjalili 2023	56.3	34:38	76.84	–	165.01	8.35	171.19	98.28	38.855	164.205
Mirmiranpour 2019	58.77	49:66	80.975	–	–	–	–	–	–	–
Miryousefiata 2021	59.3	33:27	–	6.1	146.7	–	153.2	80.7	44.3	147.8
Mobini 2017	65	34:10	96	15.4	217.2	7.86	164.992	100.542	47.693	171.1
Mohamadshahi_1 2014	51	10:32	77	–	181.33	8.3	–	–	–	–
Mohamadshahi_2 2014	51	11:31	77	–	–		220	134.6	44	206.62
Ostadrahimi 2015	50	34:26	76.19	7	172.53	7.3	201.21	102.72	44.37	178
Palacious 2020	58.75	28:32	100.9	–	104.4	6	–	–	–	–
Perraudeau 2020	52.16	30:46	91.32	–	189.13	8.73	–	–	–	–
Raygan 2019	63.6	21:33	77.9	–	138.35	–	170.6	88.75	46.2	178.35
Raygan_1 2018	61.25	–	79.2	6.7	131.3	–	146.6	73.05	44.9	142.6
Raygan_2 2018	69.4	30:30	71.25	–	124.05	–	142.75	70.35	42.15	151.35
Razmpoosh 2019	59.95	33:27	74.65	6.05	146	–	152.25	79.3	44.4	137.7
Rezaei 2017	50.31	44:46	–	–	170.15	7.55	212	124.25	46.9	186.35
Rustani 2022	43.77	–	85.62	3.3	180.6	9.525	203.06	–	–	–
Sabico 2017	47.3	40:38	77.55	–	–	–	212.7	131.478	40.6	208
Sabico 2019	47.3	40:38	–	–	181.2	–	212.69	131.478	40.6	208
Sahin 2022	50.83	80:232	–	–	–	–	–	–	–	–
Sato 2017	64.5	49:19	–	13.05	129	6.85	185.55		54.85	107.05
Shakeri 2023	52.56	15:63	77.37	–	146.83		168.5	95.07	42.83	153
Soleimani 2017	56.7	40:20	68.3	18.1	123.35	5.95	137.3	78.1	34.75	122.4
Tajadadi-Ebrahimi 2014	52.23	66:15	77.5	–	147.7	–	–	–	–	–
Toejing 2021	62.64	8:28	–	–	134.24	6.845	189.71	105.68	62.47	150.53
Tonucci 2017	51.39	26:19	74.43	7.75	138.15	5.71	183.87	88	59.36	151.78
Zhang 2020	53	245:164	71.5	––	147.38	7.73	199.73	129.16	47	–

M, Male; F, Female; FPG, Fasting plasma glucose; HbA1c, Hemoglobin A1c; TGA, Triglycerides; LDL, Low-density lipoprotein; HDL, High-density lipoprotein

### Risk of bias assessment

**Fig. 2:**
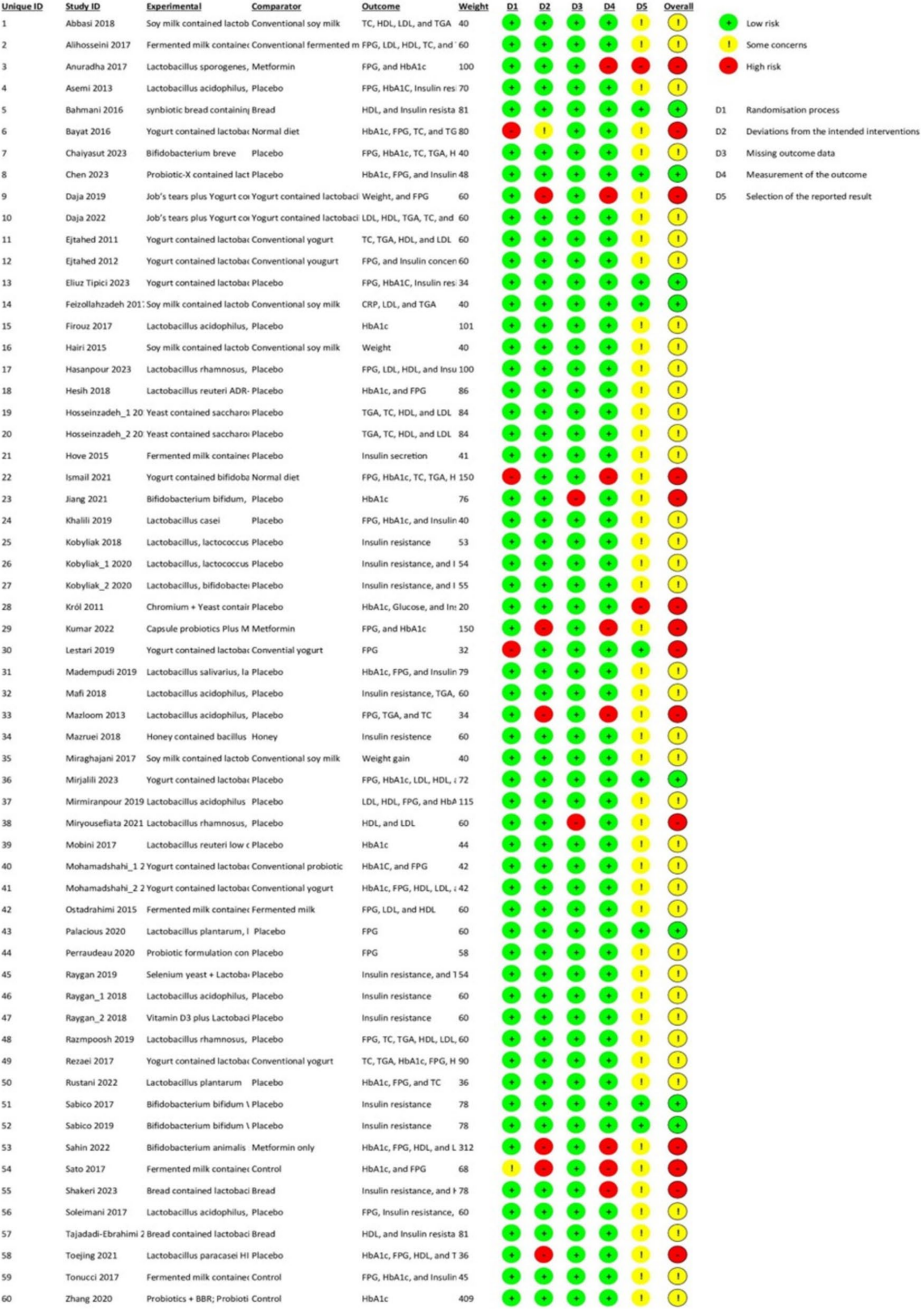
Risk of bias graph

The included studies’ risk of bias revealed 14 studies (39, 41, 44, 56, 57, 62–64, 67, 85–87, 89, 92) with a high risk of bias, 7 studies (40, 43, 47, 48, 76, 83, 84) with a low risk of bias, and the rest studies with some concerns (Fig. 2)

### Primary outcomes

#### Change in FPG (mg\dl) (See supplementary file 2)

**Fig. 3:**
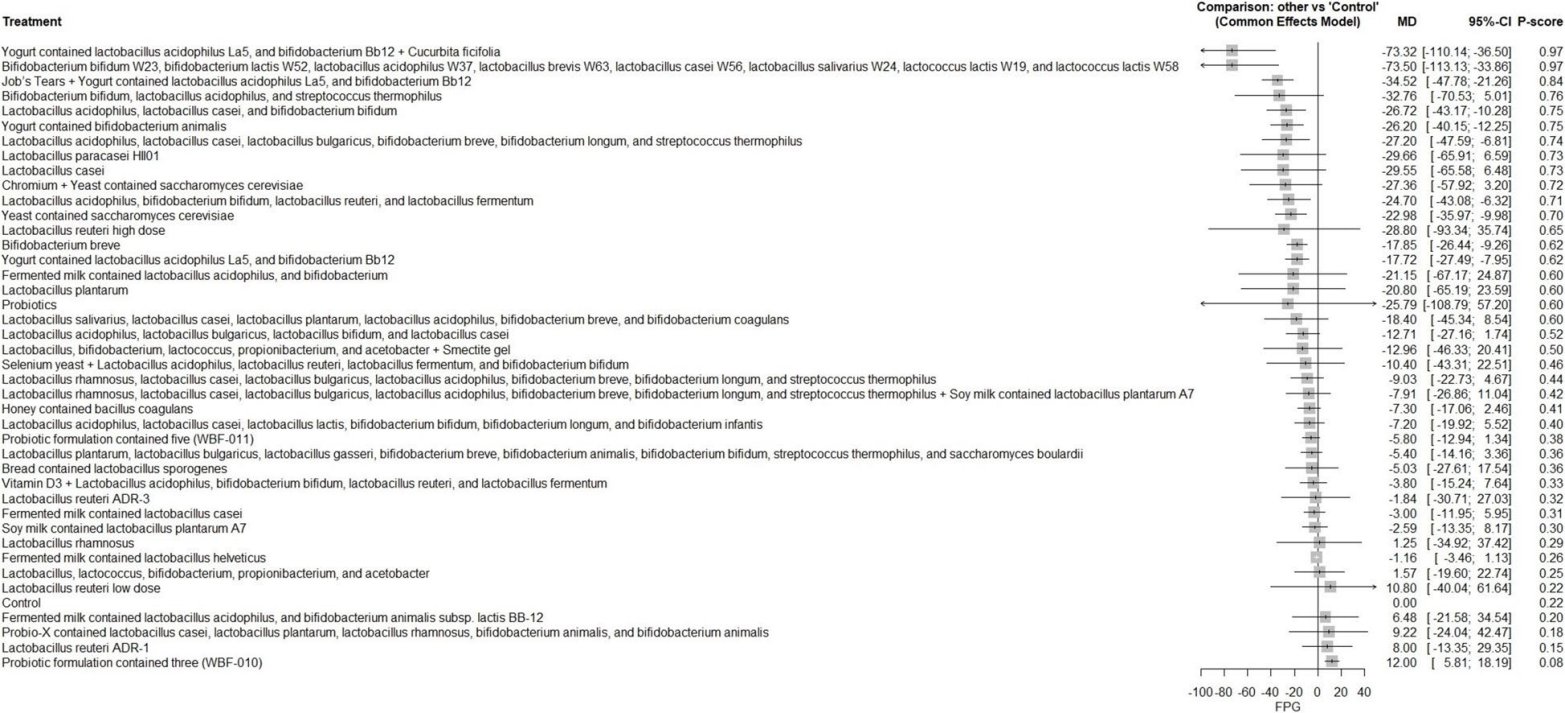
FPG

Pooled data showed that the combination of Bifidobacterium bifidum W23, Bifidobacterium lactis W52, Lactobacillus acidophilus W37, Lactobacillus brevis W63, Lactobacillus casei W56, Lactobacillus salivarius W24, Lactococcus lactis W19, and Lactococcus lactis W58 had the highest reduction in FPG [MD = − 73.50, 95% CI (− 113.13, − 33.86)]. This was followed by yogurt containing Lactobacillus acidophilus La5, and Bifidobacterium Bb12 plus Cucurbita ficifolia [MD = − 73.32, 95% CI (− 110.14, − 36.5)]. Additionally, job’s tears plus yogurt containing Lactobacillus acidophilus La5, and Bifidobacterium Bb12 significantly reduced FPG [MD = − 34.52, 95% CI (− 47.78, − 21.26)] (Fig. 3)

#### Change in HbA1c (%) *(See supplementary file 2)*

**Fig. 4:**
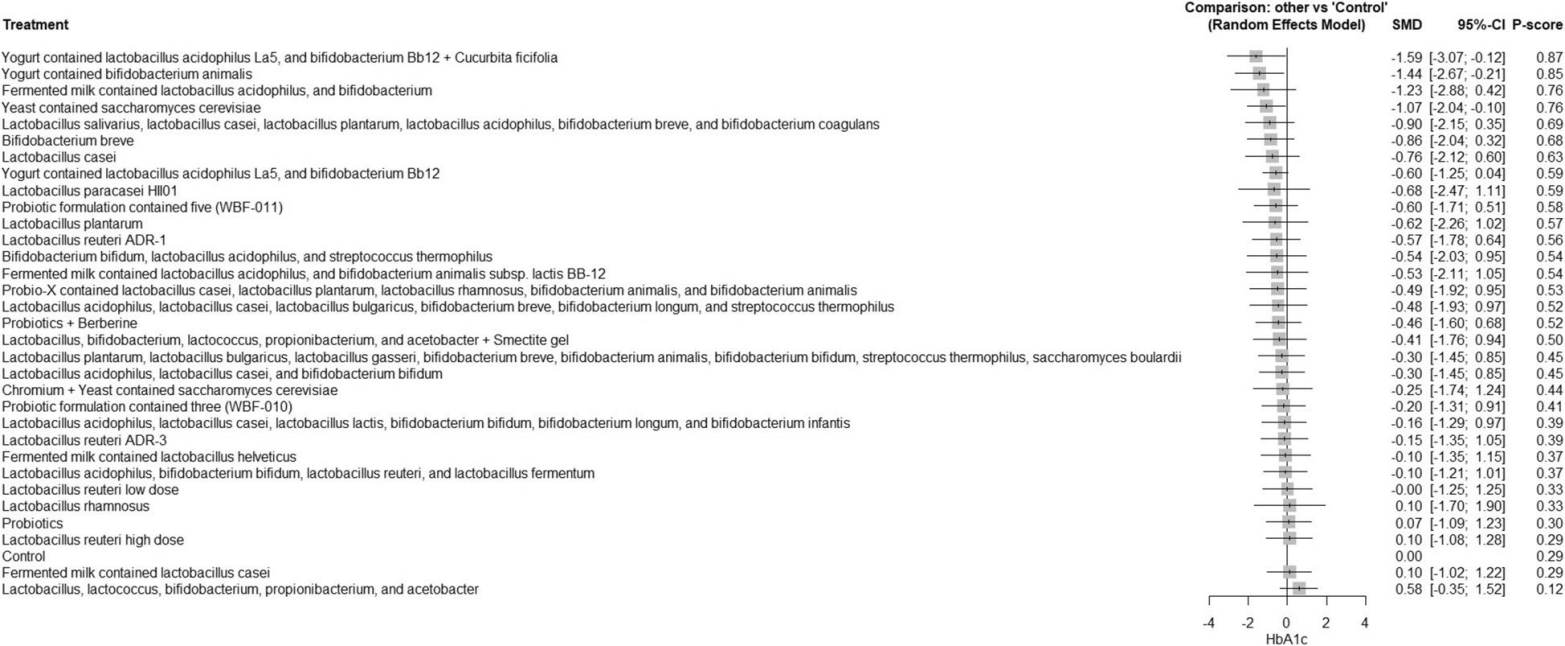
HbA1c

Pooled data showed that yogurt containing Lactobacillus acidophilus La5, and Bifidobacterium Bb12 plus Cucurbita ficifolia had the highest reduction in HbA1c among other probiotics [MD = − 1.59, 95% CI (− 3.07, − 0.12)]. This was followed by yogurt containing Bifidobacterium animalis [MD = − 1.44, 95% CI (− 2.67, − 0.21)] (Fig. 4)

#### Change in insulin resistance (HOMA-IR) *(See supplementary file 2)*

**Fig. 5:**
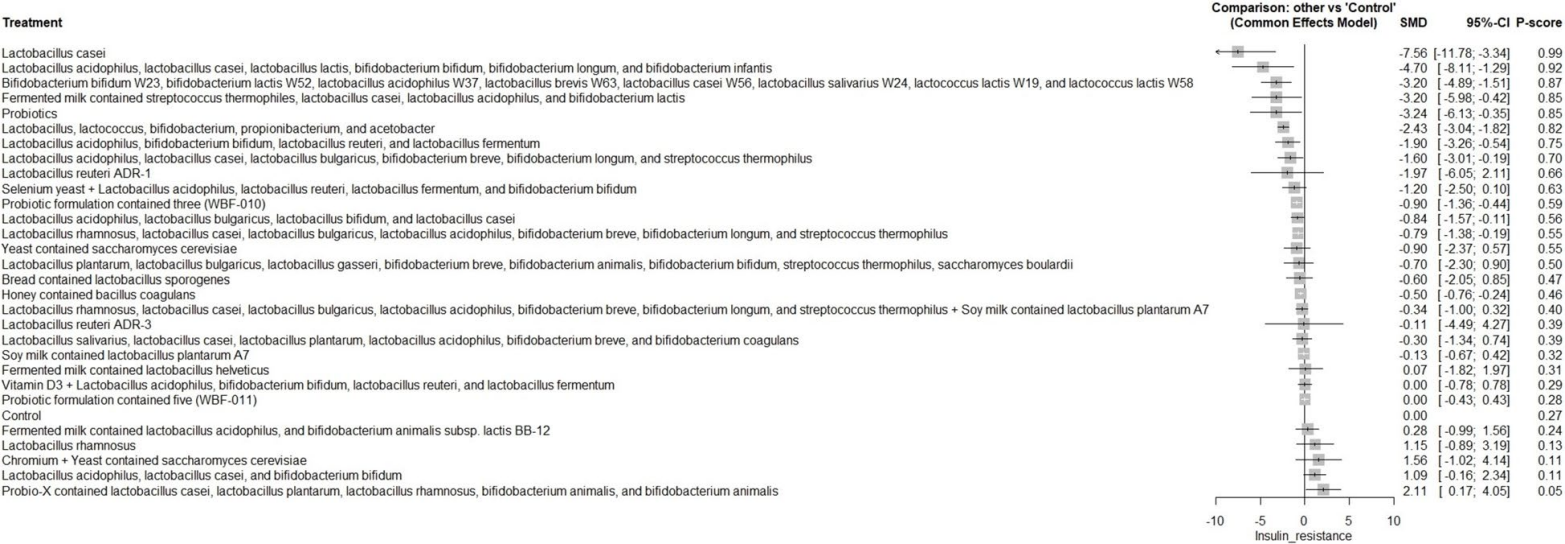
Insulin Resistance

Pooled results showed that yogurt containing Lactobacillus casei had the highest reduction in insulin resistance among other probiotics [MD = − 7.56, 95% CI (− 11.78, − 3.34)]. This was followed by combination of Lactobacillus acidophilus, Lactobacillus casei, Lactobacillus lactis, Bifidobacterium bifidum, Bifidobacterium longum, and Bifidobacterium infantis [MD = − 4.70, 95% CI (− 8.11, − 1.29)], and combination of Bifidobacterium bifidum W23, Bifidobacterium lactis W52, Lactobacillus acidophilus W37, Lactobacillus brevis W63, Lactobacillus casei W56, Lactobacillus salivarius W24, Lactococcus lactis W19, and Lactococcus lactis W58 [MD = − 3.20, 95% CI (− 4.89, − 1.51)] (Fig. 5)

#### Change in total cholesterol (mg\dl) *(See supplementary file 2)*

**Fig. 6:**
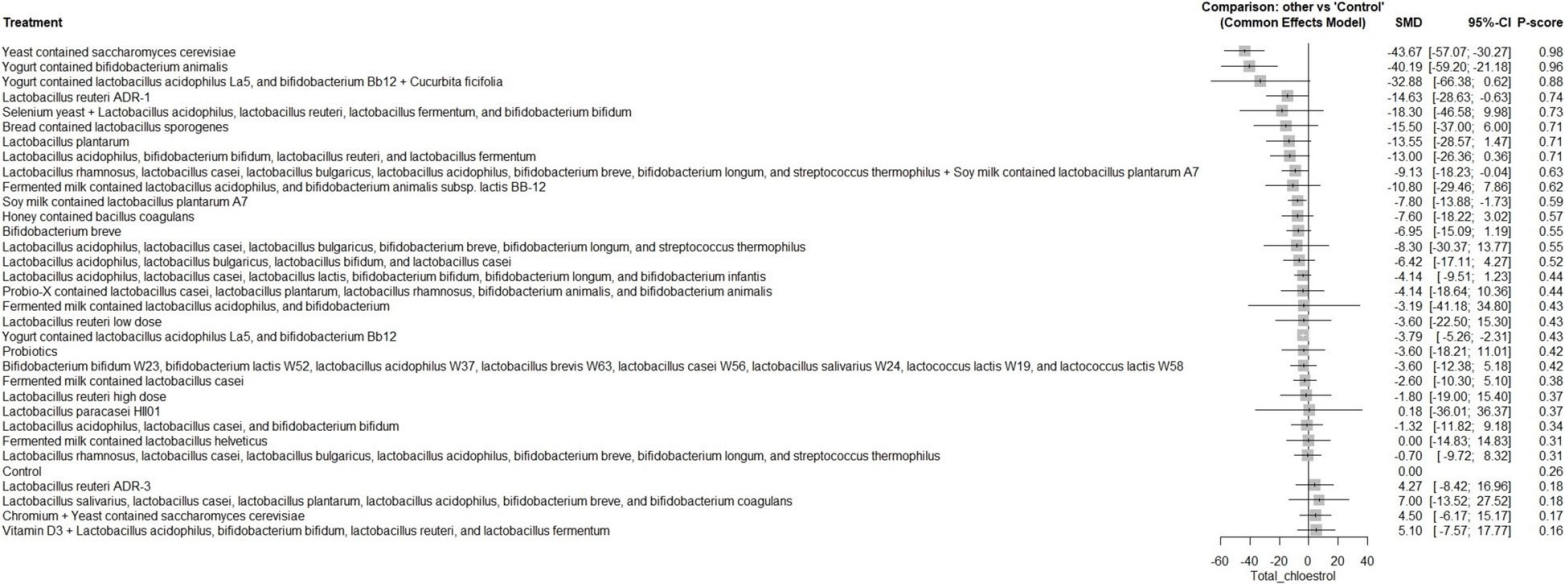
Total Cholesterol

Pooled results showed that yeast containing Saccharomyces cerevisiae had the highest reduction in total cholesterol among other probiotics [MD = − 43.67, 95% CI (− 57.07, − 30.27)]. This was followed by yogurt containing Bifidobacterium animalis [MD = − 40.19, 95% CI (− 59.20, − 21.18)] (Fig. 6)

#### Change in TAG (mg\dl) *(See supplementary file 2)*

**Fig. 7:**
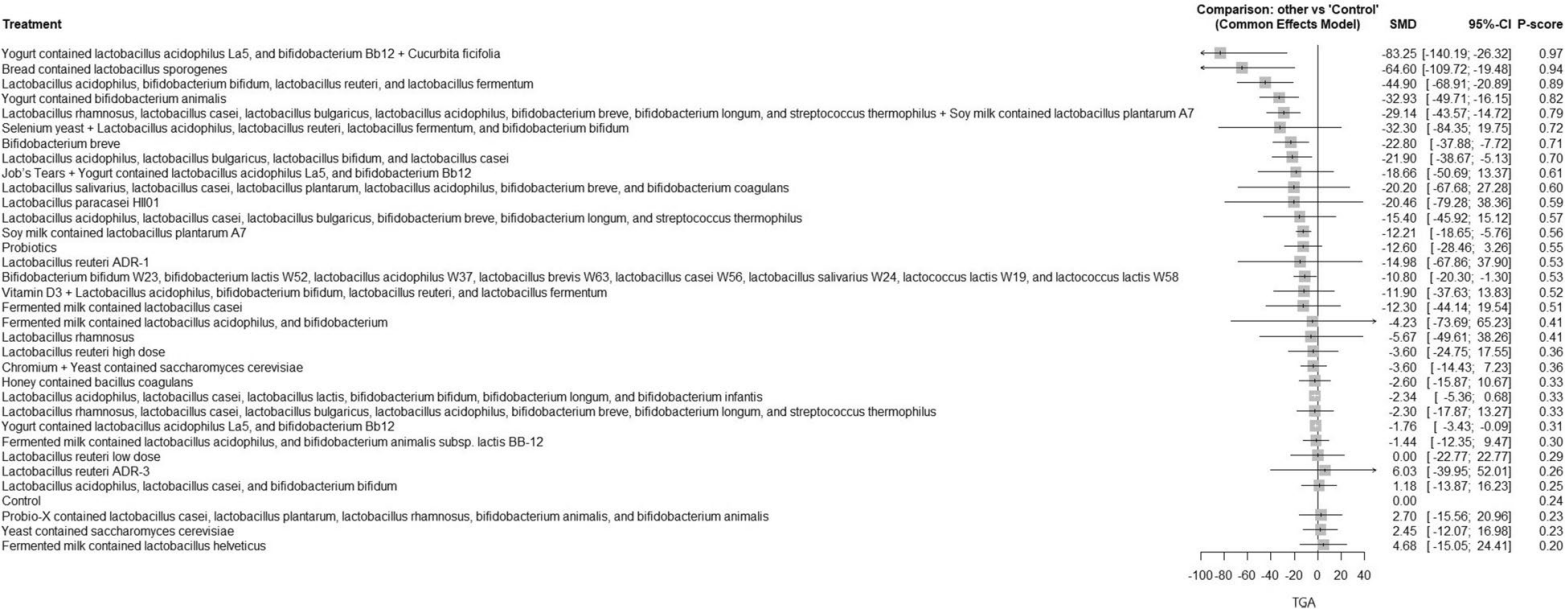
TAG

Pooled results showed that yogurt containing Lactobacillus acidophilus La5, and Bifidobacterium Bb12 plus Cucurbita ficifolia had the highest reduction in TAG among other probiotics [MD = − 83.25, 95% CI (− 140.19, − 26.32)]. This was followed by bread containing Lactobacillus sporogenes [MD = − 64.60, 95% CI (− 109.72, − 19.48)], and combination of Lactobacillus acidophilus, Bifidobacterium bifidum, Lactobacillus reuteri, and Lactobacillus fermentum [MD = − 44.90, 95% CI (− 68.91, − 20.89)] (Fig. 7)

#### Change in HDL (md\dl) *(See supplementary file 2)*

**Fig. 8:**
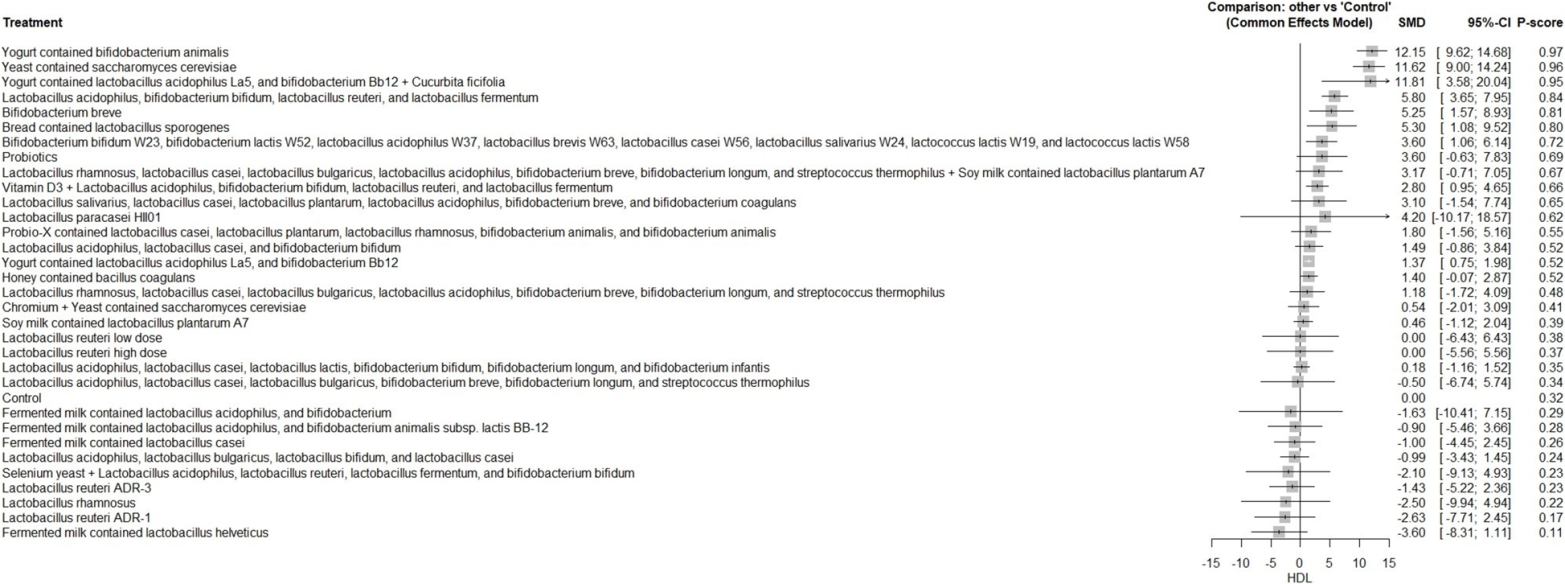
HDL

Pooled results showed that yogurt containing Bifidobacterium animalis had the highest elevation in HDL among other probiotics [MD = 12.15, 95% CI (9.62, 14.68)]. This was followed by yeast containing Saccharomyces cerevisiae [MD = 11.62, 95% CI (9.00, 14.24)], and yogurt containing Lactobacillus acidophilus La5, and Bifidobacterium Bb12 plus Cucurbita ficifolia [MD = 11.81, 95% CI (3.58, 20.04)] (Fig. 8)

#### Change in LDL (md\dl) *(See supplementary file 2)*

**Fig. 9:**
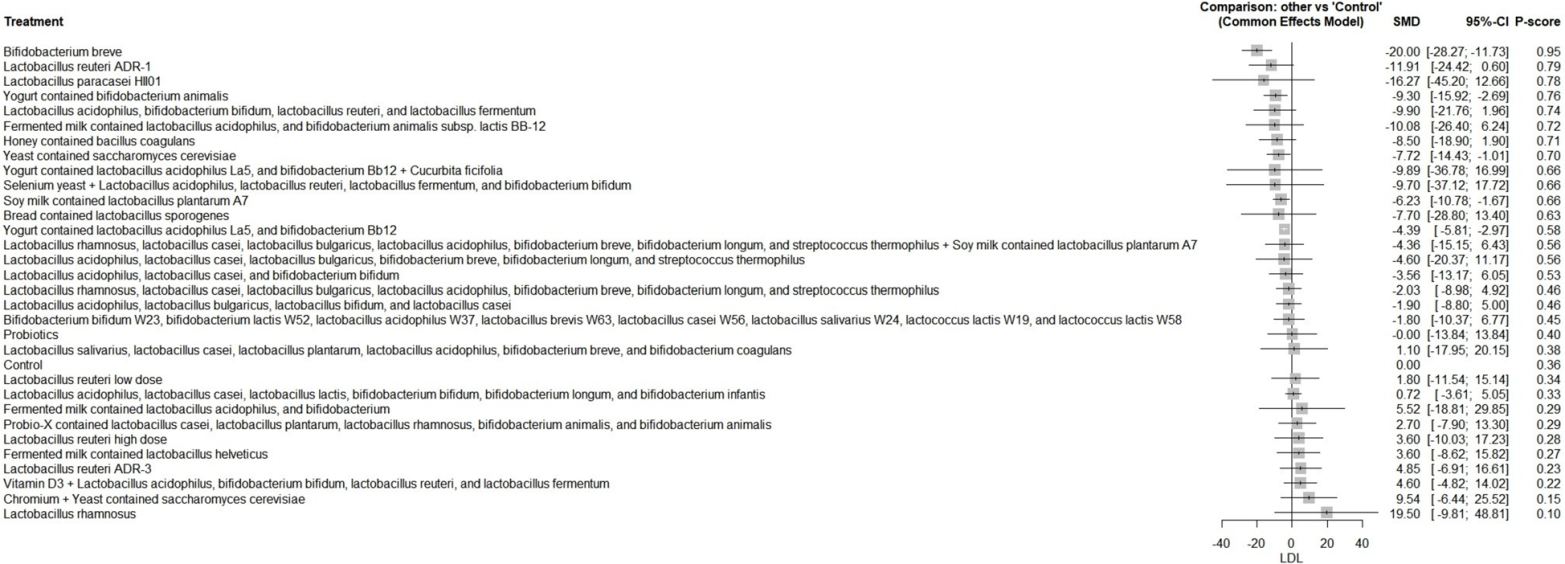
LDL

Pooled results showed that Bifidobacterium breve had the highest reduction in LDL among other probiotics [MD = − 20, 95% CI (− 28.27, − 11.73)] (Fig. 9)

#### Change in weight gain (kg) *(See supplementary file 2)*

**Fig. 10:**
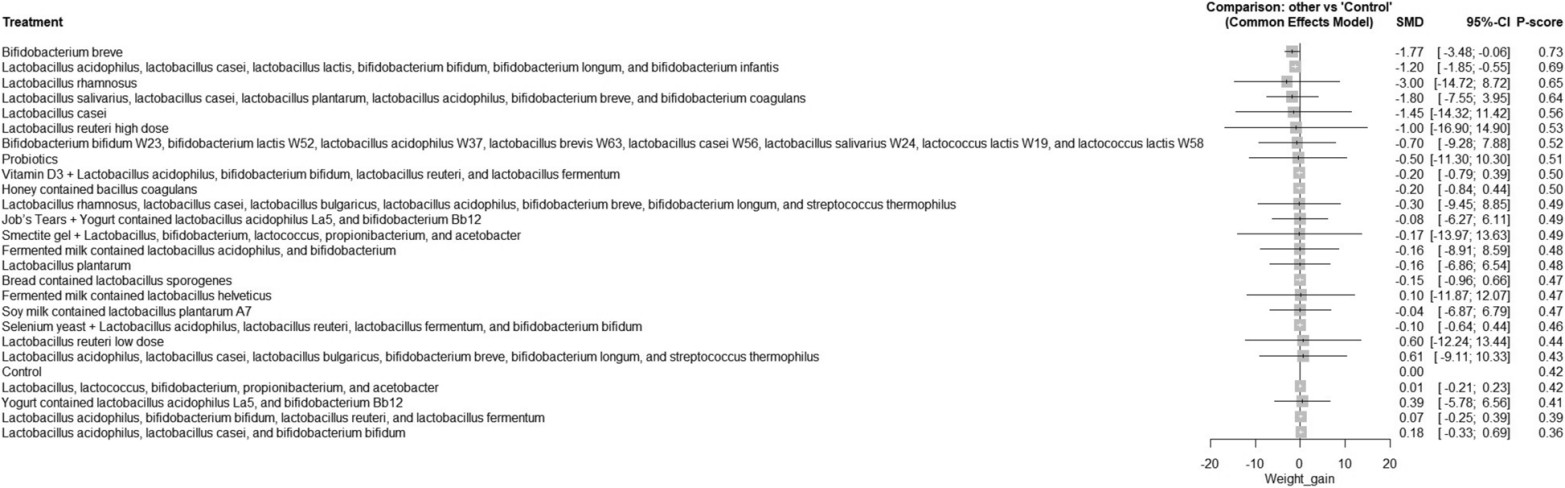
Weight gain

Pooled results showed that Bifidobacterium breve had the highest reduction in weight gain among other probiotics [MD = − 1.77, 95% CI (− 3.48, − 0.06)]. This was followed by a combination of Lactobacillus acidophilus, Lactobacillus casei, Lactobacillus lactis, Bifidobacterium bifidum, Bifidobacterium longum, and Bifidobacterium infantis [MD = − 1.20, 95% CI (− 1.85, − 0.55)] (Fig. 10)

### Secondary outcomes

#### Insulin concentration (mg\dl) (See supplementary file 3)

Pooled results showed that combination of Lactobacillus rhamnosus, Lactobacillus casei, Lactobacillus bulgaricus, Lactobacillus acidophilus, Bifidobacterium breve, Bifidobacterium longum, and Streptococcus thermophilus plus soy milk containing Lactobacillus plantarum A7 had the highest reduction in insulin concentration among other probiotics [MD = − 8.49, 95% CI (− 15.55, − 1.42)].

#### Fasting insulin (mg/dl) (See supplementary file 3)

None of the included probiotics significantly reduced the fasting insulin concentration.

#### PPG (mg\dl) (See supplementary file 3)

Pooled results showed that yeast containing Saccharomyces cerevisiae had the highest reduction in PPG among other probiotics [MD = − 38.70, 95% CI (− 52.23, − 25.17)]. This was followed by yogurt containing Bifidobacterium animalis [MD = − 29.30, 95% CI (− 44.52, − 14.08)].

#### Cholesterol/HDL ratio (See supplementary file 3)

Pooled results showed that a combination of Lactobacillus acidophilus, Bifidobacterium bifidum, Lactobacillus reuteri, and Lactobacillus fermentum had the highest reduction in cholesterol\HDL ratio among other probiotics [MD = − 1.30, 95% CI (− 1.83, − 0.77)]. This was followed by bread containing Lactobacillus sporogenes [MD = − 0.90, 95% CI (− 1.45, − 0.35)].

#### CRP (mg\dl) (See supplementary file 3)

Pooled results showed that fermented milk containing Lactobacillus casei had the highest reduction in CRP among other probiotics [MD = − 4.50, 95% CI (− 12.08, 3.08)]; However, it was not significant. This was followed by honey containing Bacillus coagulans [MD = − 1.70, 95% CI (− 2.99, − 0.41)], and combination of Lactobacillus acidophilus, Lactobacillus casei, Lactobacillus bulgaricus, Bifidobacterium breve, Bifidobacterium longum, and Streptococcus thermophilus [MD = − 1.65, 95% CI (− 3.09, − 0.22)].

#### IL-6 (pg/ml) (See supplementary file 3)

Pooled results showed that yogurt containing Bifidobacterium animalis had the highest reduction in IL-6 among other probiotics [MD = − 14.70, 95% CI (− 23.82, − 5.58)].

#### TNF-α (pg/ml) (See supplementary file 3)

Pooled results showed that yogurt containing Bifidobacterium animalis had the highest reduction in TNF-α among other probiotics [MD = − 12.70, 95% CI (− 15.61, − 9.79)]. This was followed by Lactobacillus reuteri ADR-3 [MD = − 12.60, 95% CI (− 15.81, − 9.39)], and yeast containing Saccharomyces cerevisiae [MD = − 11.10, 95% CI (− 13.85, − 8.35)].

#### Adiponectin (µg/ml) (See supplementary file 3)

None of the included probiotics significantly increased the adiponectin concentration.

#### Leptin (µg/ml) (See supplementary file 3)

None of the included probiotics significantly increased the leptin concentration.

#### Resistin (ng\ml) (See supplementary file 3)

Pooled results showed that a combination of Bifidobacterium bifidum W23, Bifidobacterium lactis W52, Lactobacillus acidophilus W37, Lactobacillus brevis W63, Lactobacillus casei W56, Lactobacillus salivarius W24, Lactococcus lactis W19, and Lactococcus lactis W58 had the highest reduction in resistin among other probiotics [MD = − 9.23, 95% CI (− 15.32, − 3.14)].

#### Fat mass (kg) (See supplementary file 3)

Pooled results showed that Bifidobacterium breve had the highest reduction in fat mass among other probiotics [MD = − 3.87, 95% CI (− 6.23, − 1.51)].

Table 3 summarizes the results of the main outcomes.

**Table 3:** Summary of the main outcomes of our NMA

	The main outcomes	The best therapeutic option	MD and 95% CI
Glycemic indices	FPG (mg/dl)	The combination of Bifidobacterium bifidum W23, Bifidobacterium lactis W52, Lactobacillus acidophilus W37, Lactobacillus brevis W63, Lactobacillus casei W56, Lactobacillus salivarius W24, Lactococcus lactis W19, and Lactococcus lactis W58	MD = − 73.50, 95% CI (− 113.13, − 33.86)
	HbA1c (%)	Yogurt containing Lactobacillus acidophilus La5, and Bifidobacterium Bb12 plus Cucurbita ficifolia	MD = − 1.59, 95% CI (− 3.07, − 0.12)
	Insulin resistance (HOMA-IR)	Yogurt containing Lactobacillus casei	MD = − 7.56, 95% CI (− 11.78, − 3.34)
Lipid profile and weight metrics	Total cholesterol (mg\dl)	Yeast containing Saccharomyces cerevisiae	MD = − 43.67, 95% CI (− 57.07, − 30.27)
	TAG (mg\dl)	Yogurt containing Lactobacillus acidophilus La5, and Bifidobacterium Bb12 plus Cucurbita ficifolia	MD = − 83.25, 95% CI (− 140.19, − 26.32)
	HDL (mg\dl)	Yogurt containing Bifidobacterium animalis	MD = 12.15, 95% CI (9.62, 14.68)
	LDL (mg\dl)	Bifidobacterium breve	MD = − 20, 95% CI (− 28.27, − 11.73)
	Weight gain (kg)	Bifidobacterium breve	MD = − 1.77, 95% CI (− 3.48, − 0.06)

*MD* mean difference, *FPG* fasting plasma glucose, *HbA1c* Hemoglobin A1c, *TGA* triglycerides, *LDL* low density lipoprotein, *HDL* high density lipoprotein

### Meta-regression analysis

The analyses showed no significant moderating effects of age or baseline HbA1c on the primary outcomes, including fasting blood glucose (FBG), HbA1c, insulin resistance, insulin levels, postprandial glucose (PPG), and various metabolic and inflammatory markers. Regarding the age, the OR for fasting blood glucose (FBG) was 1.01 (95% CI: 0.94–1.08, *p* = 0.8296), and for insulin resistance, the OR was 1.03 (95% CI: 0.97–1.10, *p* = 0.3397). Similarly, inflammatory markers such as C-reactive protein (CRP), interleukin-6 (IL-6), and tumor necrosis factor-alpha (TNF-α) also did not demonstrate significant associations with age (*p* > 0.05 for all). Regarding HbA1c, none of the associations reached statistical significance either. For instance, the OR for insulin resistance was 1.03 (95% CI: 0.96–1.10, *p* = 0.4367), while for postprandial glucose, the OR was 0.45 (95% CI: 0.06–2.75, *p* = 0.4488). Lipid profile parameters, including total cholesterol, triglycerides, HDL, and LDL, were also not significantly affected by HbA1c levels (*p* > 0.05 for all) (Table 4).

**Table 4:** Meta-regression analysis of the effect of age and base line HbA1c on the study outcomes

Outcome	Age		Baseline HbA1c	
	OR (95% CI),	p-value	OR (95% CI),	p-value
FBG	1.01 (0.94, 1.08)	0.8296	8.72 (0.08, 939.91)	0.3643
HbA1c	1.00 (0.94, 1.08)	0.9208	0.23 (0.61, 12.3)	0.2134
Insulin Resistance	1.03 (0.97, 1.10)	0.3397	1.03 (0.96, 1.10)	0.4367
Insulin Concentration	1.01 (0.05, 19.87)	0.9901	0.89 (0.02, 52.34)	0.9554
Fasting Insulin	1.24 (0.17, 8.86)	0.8202	1.08 (0.13, 8.72)	0.9495
Postprandial Glucose (PPG)	0.57 (0.13, 2.46)	0.4477	0.45 (0.06, 2.75)	0.4488
Total Cholesterol	0.62 (0.14, 2.82)	0.5377	0.73 (0.01, 2.62)	0.3379
Triglycerides (TGA)	0.93 (0.23, 3.75)	0.9217	1.06 (0.00, 9.45)	0.9391
HDL	0.67 (0.10, 4.41)	0.6792	2.32 (0.22, 24.21)	0.4827
LDL	0.81 (0.19, 3.35)	0.7642	0.91 (0.01, 4.40)	0.4682
Cholesterol/HDL Ratio	0.97 (0.91, 1.03)	0.3464	0.97 (0.90, 1.04)	0.4102
CRP	1.02 (0.97, 1.07)	0.4496	1.02 (0.96, 1.08)	0.5204
IL-6	0.95 (0.82, 1.09)	0.4712	0.95 (0.83, 1.10)	0.5091
TNF-α	1.07 (0.83, 1.39)	0.6098	1.05 (0.81, 1.35)	0.7148
Adiponectin	1.00 (0.97, 1.03)	0.9755	1.00 (0.97, 1.03)	0.9405
Leptin	0.96 (0.41, 2.23)	0.9287	0.88 (0.37, 2.10)	0.7734
Resistin	1.21 (0.44, 3.34)	0.7136	1.17 (0.41, 3.32)	0.7738
Fat Mass	1.02 (0.46, 2.28)	0.9538	1.09 (0.48, 2.48)	0.8310
Weight Gain	0.97 (0.94, 1.01)	0.1098	0.97 (0.93, 1.01)	0.1422

OR: Odds Ratio, CI: Confidence interval

## Discussion

Our NMA gave a thorough analysis of the effectiveness of several probiotics in the treatment of T2DM. Herein, we examined the direct and indirect evidence from various research that calculated the probiotics’ effectiveness in treating T2DM. Our study produced important results regarding the effectiveness of several probiotics in key clinical parameters associated with T2DM.

Our results revealed significant findings related to the efficacy of various probiotics in lowering FPG. This was similar to the result reported by Bakhtiary et al., (94) who reported that probiotic, prebiotic, and synbiotic supplementation significantly reduced FPG. Additionally, Samah et al., (30) reported that probiotics significantly lowered FPG. In our study probiotics containing a combination of Bifidobacterium and Lactobacillus strains had the highest reduction of FPG. This was similar to Raygan et al., (79) which was conducted in patients with T2DM and coronary heart disease. They found that the intervention, during which the strains of Bifidobacterium bifidum, Lactobacillus casei, and Lactobacillus acidophilus were ingested for 12 weeks, significantly decreased the FPG. In contrast to our result, Dong et al., (95) reported in a review and meta-analysis, which described and evaluated 18 RCT studies, that using Lactobacillus or Bifidobacterium probiotic foods or supplements as interventions showed no significant difference in the FPG level when compared to the control group [p = 0.44], but their result was based on 7 reviewed studies [642 participants].

Regarding HbA1c, our results showed that yogurt containing Lactobacillus acidophilus La5, and Bifidobacterium Bb12 plus Cucurbita ficifolia had the highest reduction in HbA1c among other probiotics. In contrast to our result, Dong et al., (95) reported that the using of Lactobacillus or Bifidobacterium probiotic foods or supplements as interventions showed no significant difference in HbA1c between the probiotic and control groups [p = 0.20], but the meta-analysis was performed based on only 2 studies (199 participants).

Regarding IR, our results, in addition, revealed that yogurt containing Lactobacillus casei had the highest reduction in insulin resistance among other probiotics. Similar findings were made by Raygan et al., (79) who discovered that a 12-week intervention involving the ingestion of, Lactobacillus casei, Lactobacillus acidophilus, and Bifidobacterium bifidum, strains dramatically reduced insulin resistance. Krüger et al., (96) reported that from 3 RCTs involving 161 individuals with Alzheimer’s disease receiving Lactobacillus and Bifidobacterium strains, probiotics significantly improved insulin resistance. In addition, Pan et al., (97) reported that the use of Bifidobacterium lactis was statistically significant in reducing Insulin resistance.

In terms of lipid profile, the yogurt containing Bifidobacterium animalis had the highest elevation in HDL among other probiotics. Our results contrast with a meta-analysis by Amir et al., (98) who reported that patients with diabetic nephropathy receiving probiotics compared to a control group did not experience any significant improvement in HDL levels. Moreover, a clinical trial by Yujin et al., (99) indicated that there were no significant changes in HDL levels. These changes were not affected by different periods with varying amounts of Bifidobacterium animalis ingestion compared to periods without Bifidobacterium animalis in healthy young adults.

Regarding LDL, the Bifidobacterium breve had the highest reduction in LDL among other probiotics. Our results aligned with the meta-analysis reported by Xiao et al. (100), who found that the probiotic treatments had a greater impact on decreasing LDL concentration than the control interventions. Moreover, Kim et al., (101) clinical trial reported that Both the low- and high-dose Bifidobacterium breve groups significantly lowered LDL levels in contrast to the placebo group in adults with Moderate Hypercholesterolemia. Our analysis also demonstrated the efficacy of Lactobacillus reuteri ADR-1 and Lactobacillus paracasei in lowering the Plasma LDL levels. This also was reported by Heish et al., (20) who observed that, the Lactobacillus reuteri ADR-1 ingestion group experienced decreases in LDL, cholesterol, and free fatty acids, but only cholesterol was significantly different from the control group. Bifidobacterium breve was more effective at lowering the LDL and preventing weight gain.

In terms of TAG, pooled results showed that yogurt containing Lactobacillus acidophilus La5, and Bifidobacterium Bb12 plus Cucurbita ficifolia had the highest reduction in TAG among other probiotics In contrast, Ejtahed et al., (46) reported that TAG was not significantly different from baseline in the probiotic group taking yogurt containing Lactobacillus acidophilus, and Bifidobacterium. Bread containing Lactobacillus sporogenes, and a combination of Lactobacillus acidophilus, Bifidobacterium bifidum, Lactobacillus reuteri, and Lactobacillus fermentum demonstrated significant efficacy in lowering plasma TAG levels as well. This was aligned with the findings of the meta-analysis by Wu et al., (102) which reported taking synbiotic foods with Lactobacillus sporogenes and inulin had a significant positive impact on TAG.

Regarding total cholesterol level, our results showed that yeast containing Saccharomyces cerevisiae had the highest reduction in total cholesterol among others. Boroujeni et al., (103) reported that the tendency of total cholesterol to decrease after consuming yeast containing Saccharomyces cerevisiae. However, this reduction did not reach significant levels. Yogurt containing Bifidobacterium animalis, yogurt containing Lactobacillus acidophilus La5, and Bifidobacterium Bb12 plus Cucurbita ficifolia showed a significant reduction in total cholesterol level. In contrast to our findings, Yujin et al., (99) reported that there were no detectable changes in total cholesterol after Bifidobacterium animalis ingestion compared to periods with no Bifidobacterium animalis. Our study’s unique combination of Lactobacillus acidophilus La5, and Bifidobacterium Bb12 plus Cucurbita ficifolia in yogurt and the inclusion of yeast containing Saccharomyces cerevisiae could have synergistic effects on TAG and total cholesterol reduction that differ from the results reported in previous studies. This may explain the discrepancy between our results and the literature.

Numerous probiotics showed effective interplay between various T2DM-related parameters. In terms of lowering FPG and HbA1c results, the combination of probiotics containing Bifidobacterium and Lactobacillus strains with yogurt containing yogurt formulations containing Lactobacillus acidophilus La5, Bifidobacterium Bb12 with Cucurbita ficifolia were optimum. Additionally, Lactobacillus casei proved to be more effective at lowering insulin resistance. Therefore, combining the aforementioned probiotics may have a synergistic impact and improve the results of FPG, HbA1c, and insulin resistance. In addition, other probiotic combinations showed promising efficacy in different parameters associated with cardiovascular events. The combination of Bifidobacterium breve, and yogurt containing Bifidobacterium animalis was superior in LDL and HDL outcomes. Whereas the combination of yogurt containing Lactobacillus acidophilus La5, and Bifidobacterium Bb12 plus Cucurbita ficifolia and yeast containing Saccharomyces cerevisiae was superior in TAG and total cholesterol outcomes, respectively.

This study has several limitations. There was notable variability in probiotic dosages, formulations, and study durations, with many being short-term, which may be the source of potential heterogeneity and affect the generalizability of the findings. Some included studies lacked complete outcome data, and we excluded a few studies (39, 63, 85) combining probiotics with metformin due to network inconsistencies. Additionally, potential publication bias cannot be ruled out, and combining different types of control groups introduced heterogeneity that may complicate interpretation. Overall, the clinical and methodological heterogeneity among studies suggests caution in applying these results broadly.

## Conclusion

Our network meta-analysis elucidates the potential of probiotic interventions in improving glycemic control and lipid profiles among individuals with type 2 diabetes mellitus. Particularly, combinations of various strains of Lactobacillus, Bifidobacterium, and yogurt formulations containing Lactobacillus acidophilus La5, Bifidobacterium Bb12 with Cucurbita ficifolia, and Saccharomyces cerevisiae yeast show promise as adjunctive therapies for managing T2DM.

## Supplementary Information

Additional file 1.

Additional file 2.

Additional file 3.

Additional file 4.

## Data Availability

No datasets were generated or analysed during the current study.

## Abbreviations

CI: Confidence interval
CRP: C-reactive protein
FPG: Casting plasma glucose
HbA1c: Hemoglobin A1c
HDL: High-density lipoprotein
IL-6: Interleukin-6
LDL: Low-density lipoprotein
NWA: Network meta-analysis
PPG: Postprandial glucose
RCTs: Randomized controlled trials
MD: Mean difference
T2DM: Type 2 diabetes mellitus
TAG: Triacylglycerol
TNF-α: Tumor necrosis factor-alpha
WOS: Web of science
