# Supplementary figures and images for: Network meta-analysis of randomized control trials evaluating the effectiveness of various probiotic formulations in patients with type 2 diabetes mellitus
Source: Diabetol Metab Syndr. 2025 Jul 11;17:265. doi: 10.1186/s13098-025-01841-2 (PMC12254980; doi:10.1186/s13098-025-01841-2)

**Risk of bias summary**

**
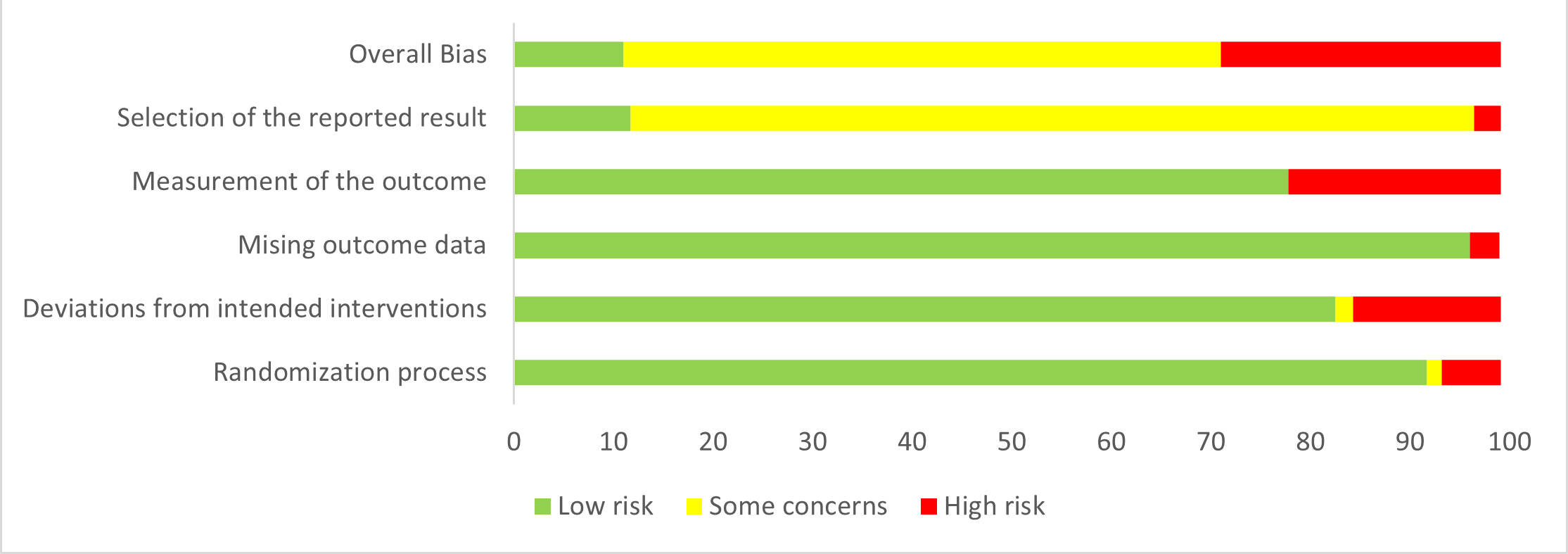
**

Supplement: Supplementary file 1 — Additional file 1. [file 13098_2025_1841_MOESM1_ESM.docx]
