# Supplementary material for: Network meta-analysis of randomized control trials evaluating the effectiveness of various probiotic formulations in patients with type 2 diabetes mellitus
Source: Diabetol Metab Syndr. 2025 Jul 11;17:265. doi: 10.1186/s13098-025-01841-2 (PMC12254980; doi:10.1186/s13098-025-01841-2)

**1. FPG**

**Quantifying heterogeneity / inconsistency:**

tau^2 = 62.0371; tau = 7.8764; I^2 = 24.3% [0.0%; 59.9%]

**Tests of heterogeneity (within designs) and inconsistency (between designs):**

Q d.f. p-value

Total 17.18 13 0.1911


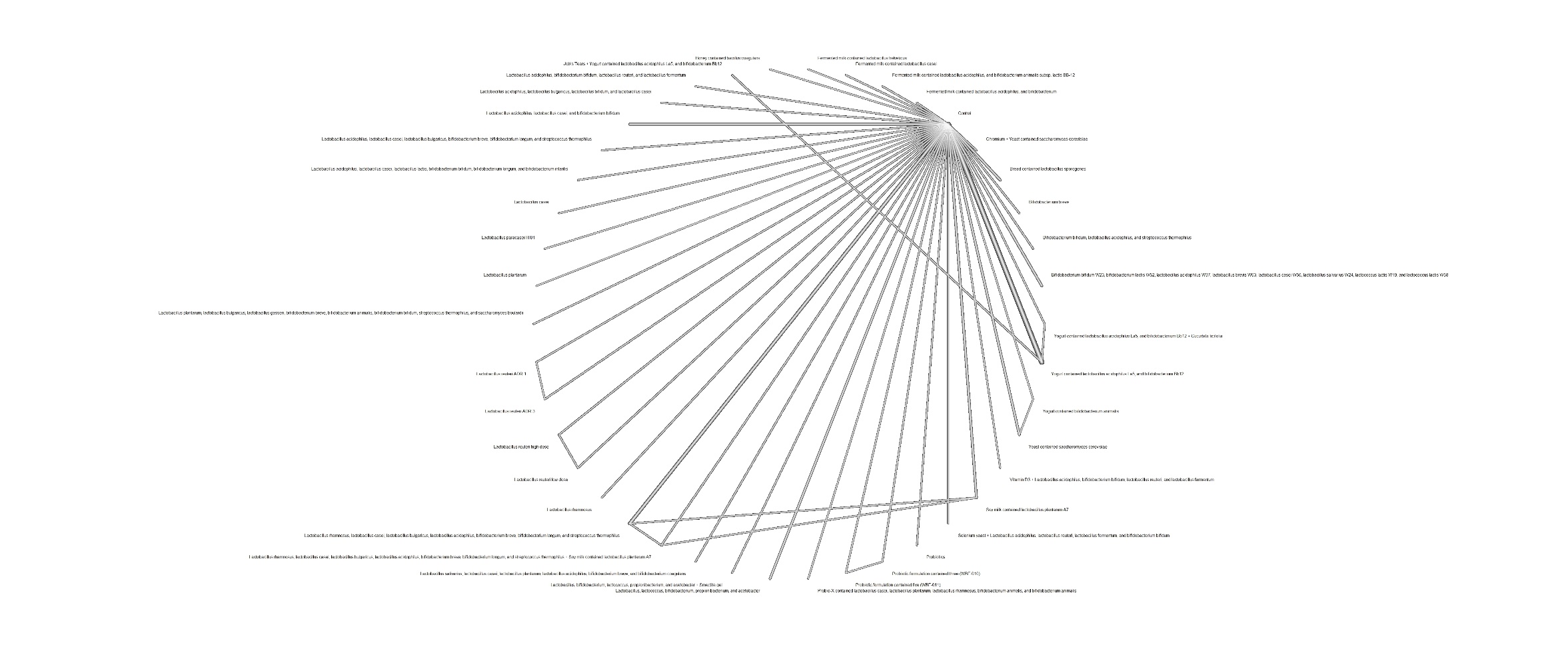

**2. HbA1c**

**Quantifying heterogeneity / inconsistency:**

tau^2 = 0.3184; tau = 0.5643; I^2 = 64.3% [19.4%; 84.2%]

**Tests of heterogeneity (within designs) and inconsistency (between designs):**

Q d.f. p-value

Total 16.79 6 0.0101


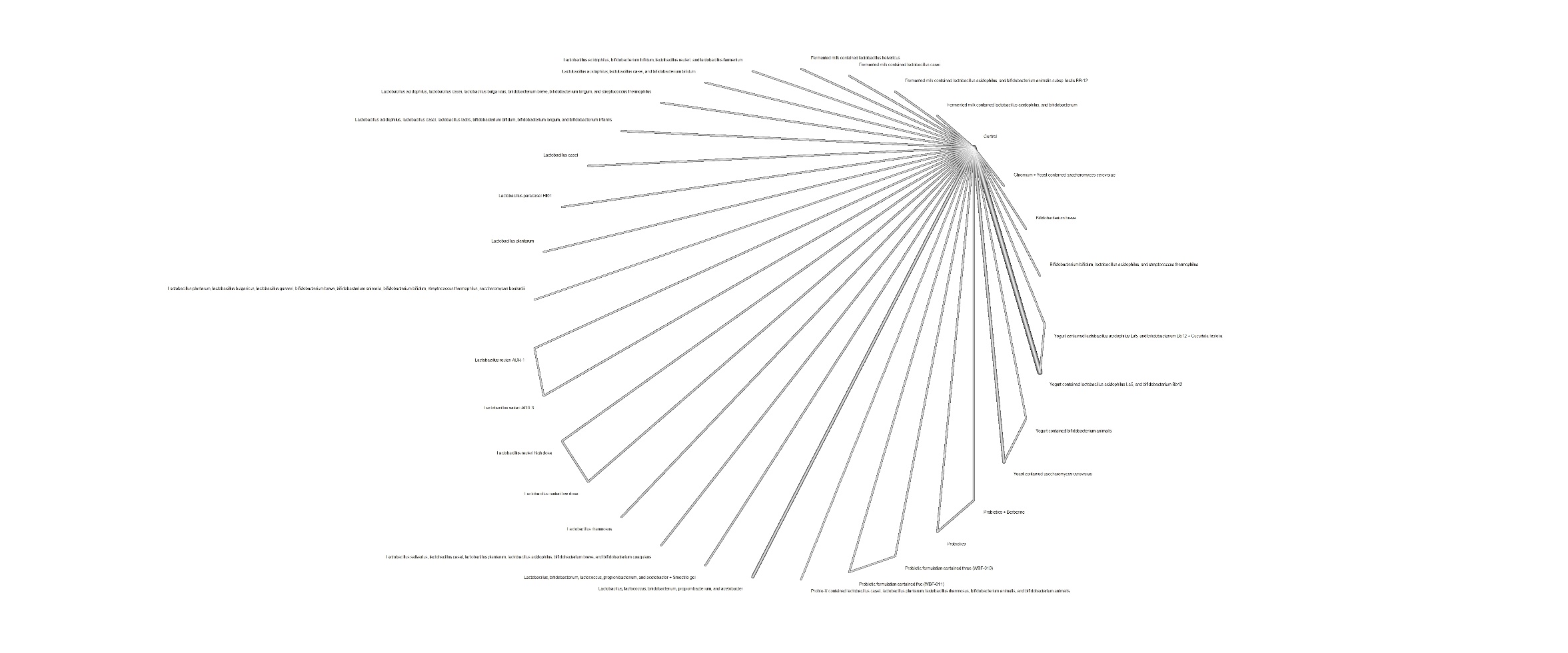

**3. Insulin resistance**

**Quantifying heterogeneity / inconsistency:**

tau^2 = 0.2976; tau = 0.5455; I^2 = 33.9% [0.0%; 76.8%]

**Tests of heterogeneity (within designs) and inconsistency (between designs):**

Q d.f. p-value

Total 4.54 3 0.2086


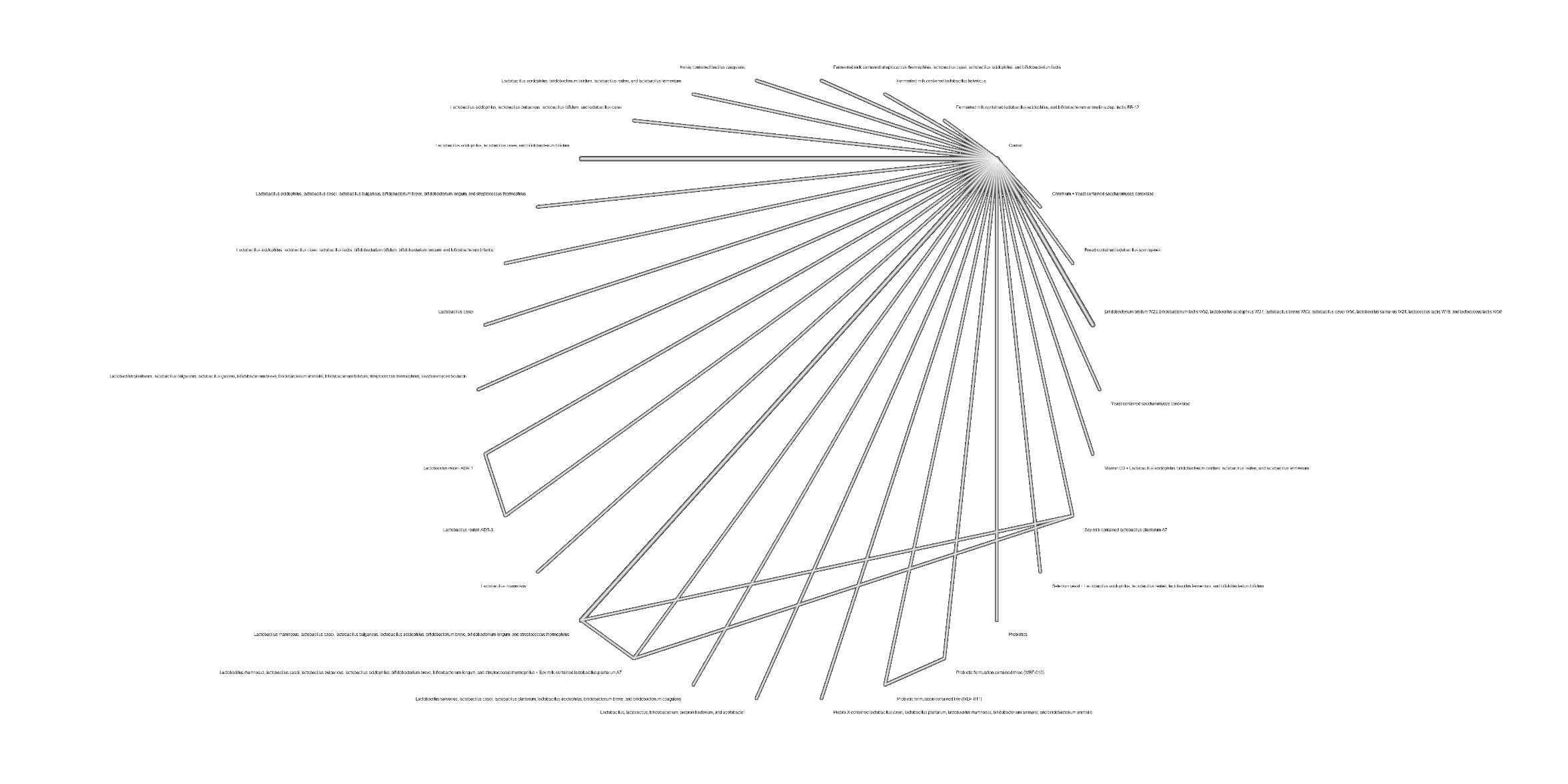

**4. Total cholesterol**

**Quantifying heterogeneity / inconsistency:**

tau^2 = 11.5179; tau = 3.3938; I^2 = 14.4% [0.0%; 54.2%]

**Tests of heterogeneity (within designs) and inconsistency (between designs):**

Q d.f. p-value

Total 12.84 11 0.3037


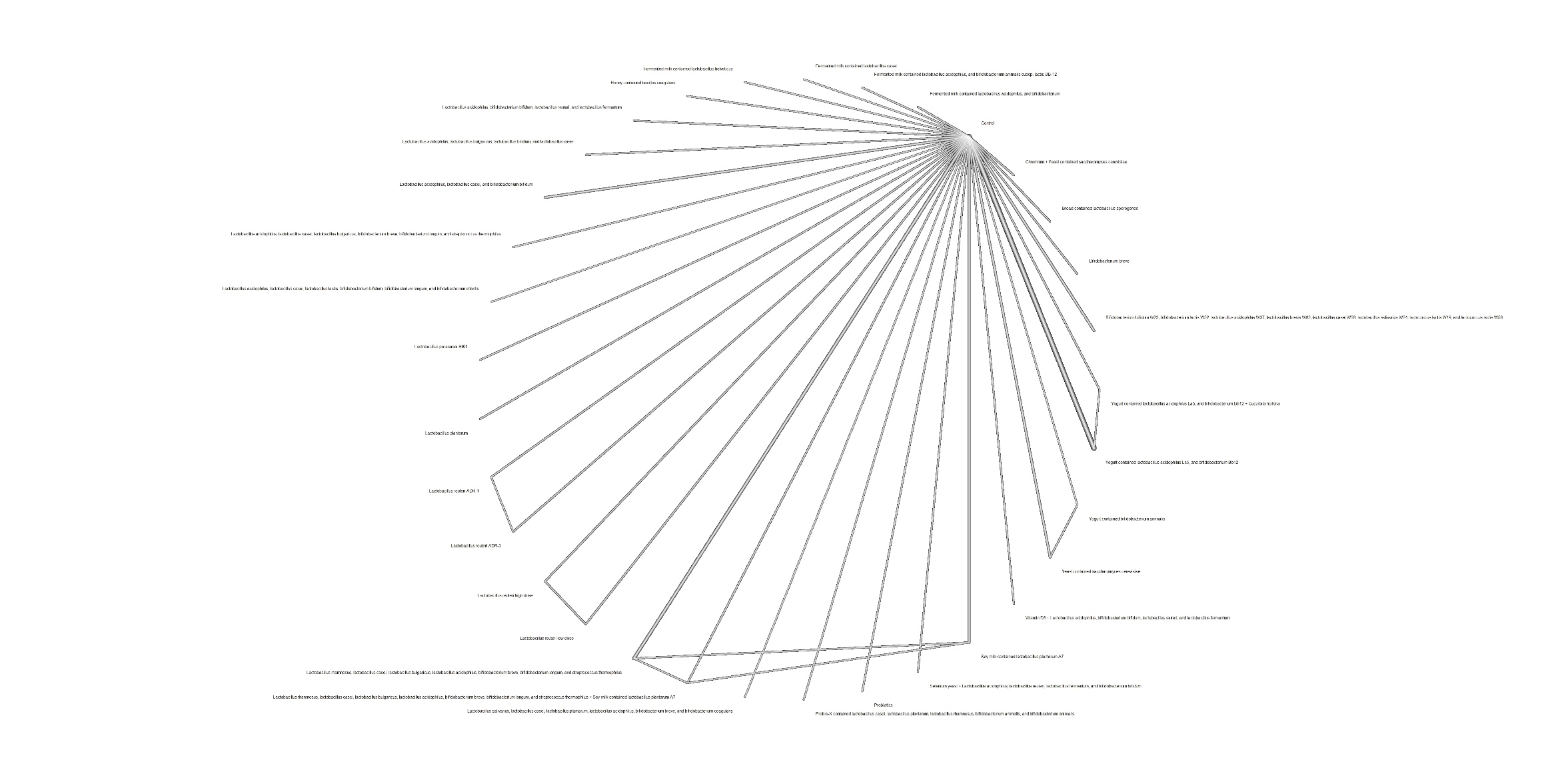

**5. TAG**

**Quantifying heterogeneity / inconsistency:**

tau^2 = 0; tau = 0; I^2 = 0% [0.0%; 56.6%]

**Tests of heterogeneity (within designs) and inconsistency (between designs):**

Q d.f. p-value

Total 9.32 12 0.6755


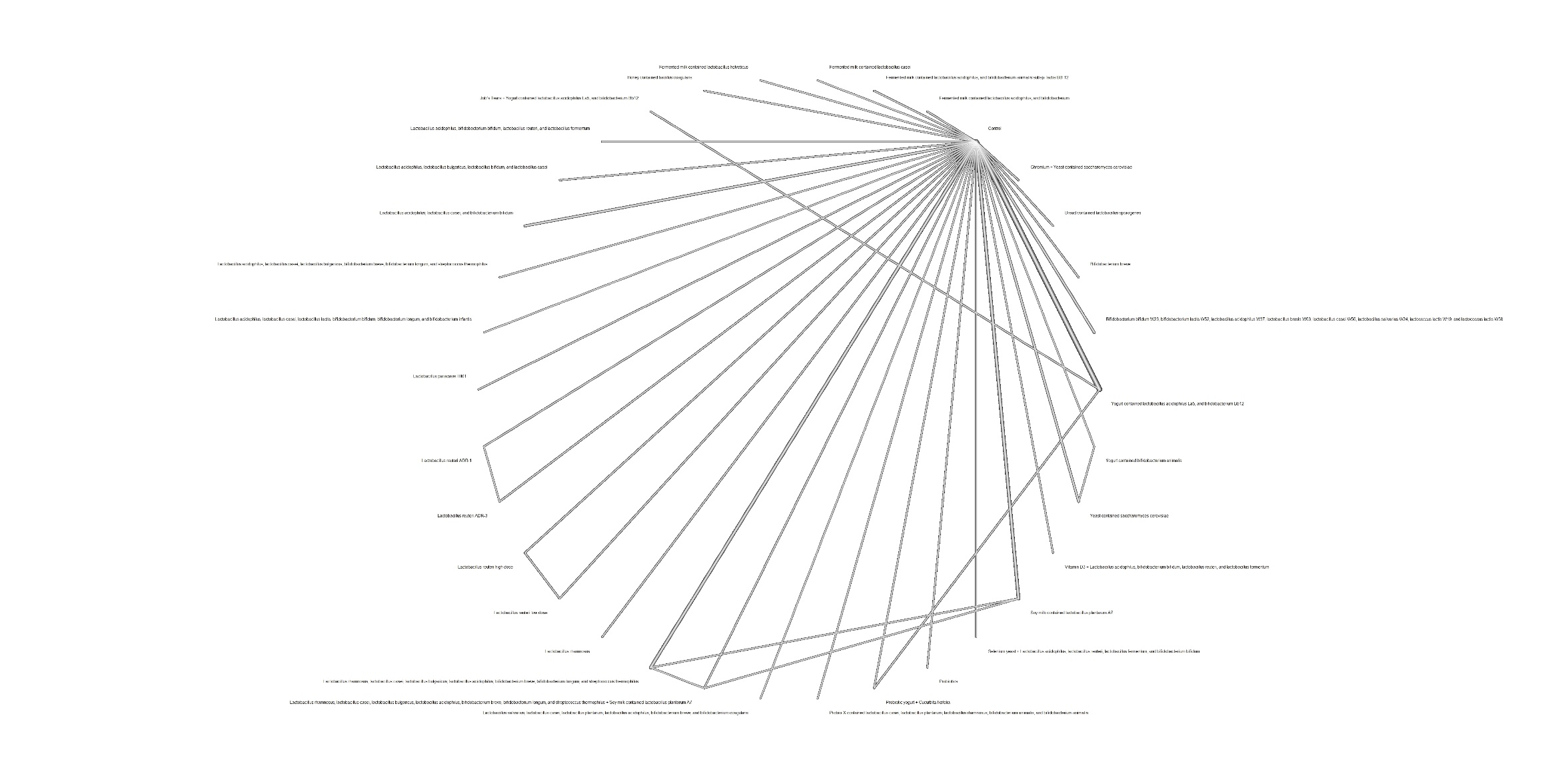

**6. HDL**

**Quantifying heterogeneity / inconsistency:**

tau^2 = 0; tau = 0; I^2 = 0% [0.0%; 56.6%]

**Tests of heterogeneity (within designs) and inconsistency (between designs):**

Q d.f. p-value

Total 5.93 12 0.9194


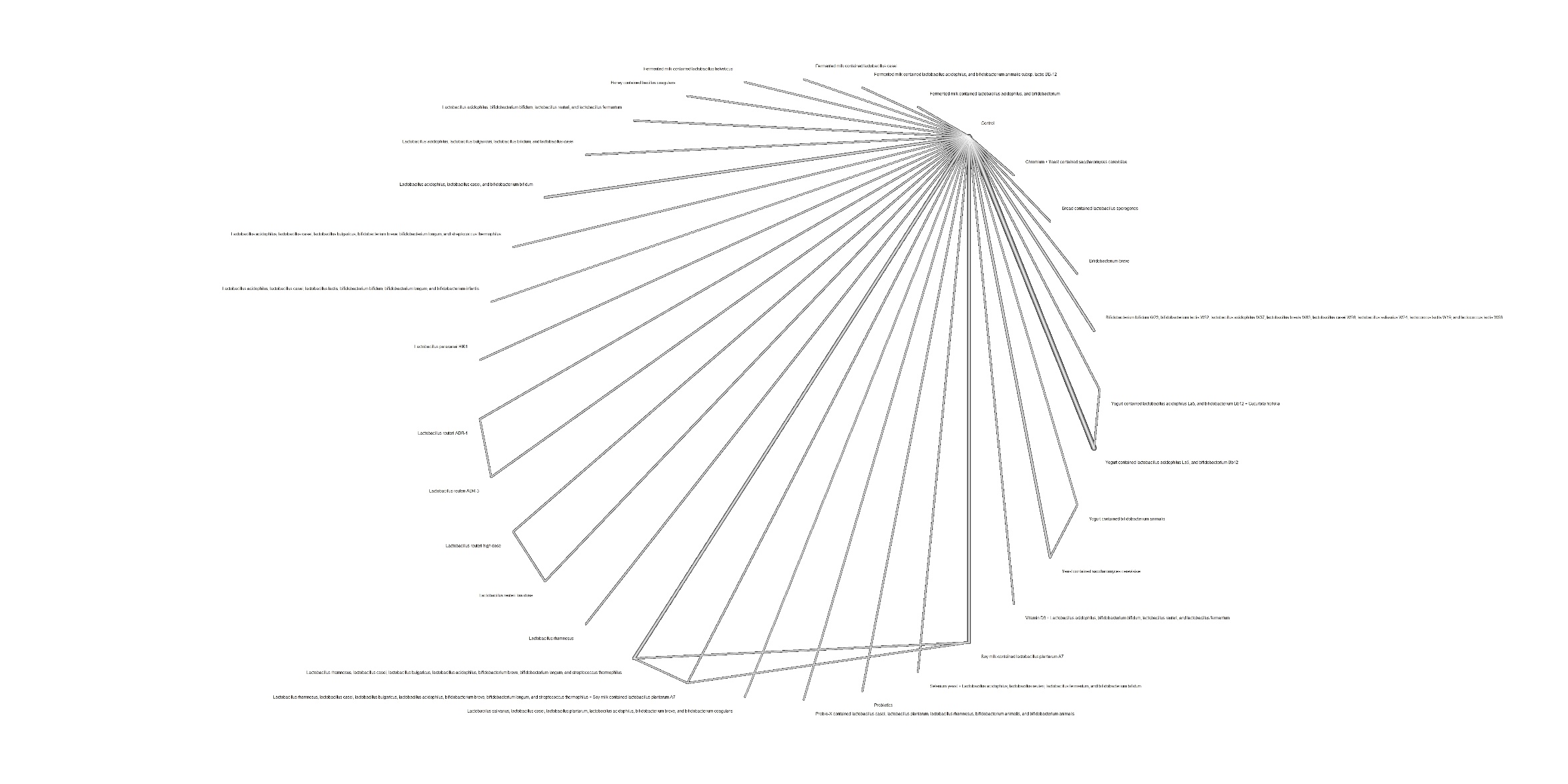

**7. LDL**

**Quantifying heterogeneity / inconsistency:**

tau^2 = 10.4175; tau = 3.2276; I^2 = 20.1% [0.0%; 57.9%]

**Tests of heterogeneity (within designs) and inconsistency (between designs):**

Q d.f. p-value

Total 15.01 12 0.2407


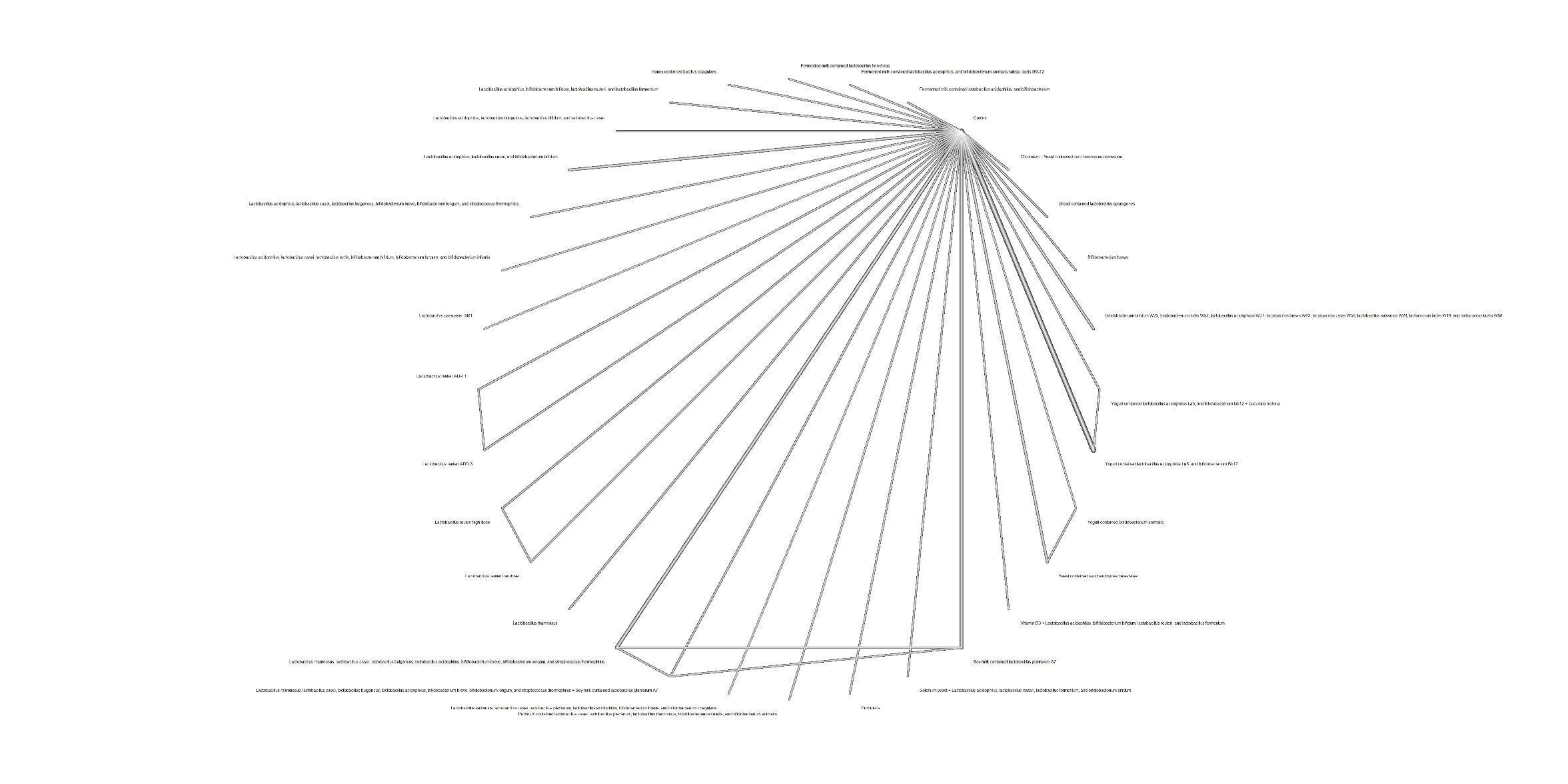

**8. Weight gain**

**Quantifying heterogeneity / inconsistency:**

tau^2 = 0; tau = 0; I^2 = 0% [0.0%; 67.6%]

**Tests of heterogeneity (within designs) and inconsistency (between designs):**

Q d.f. p-value

Total 3.41 7 0.8446


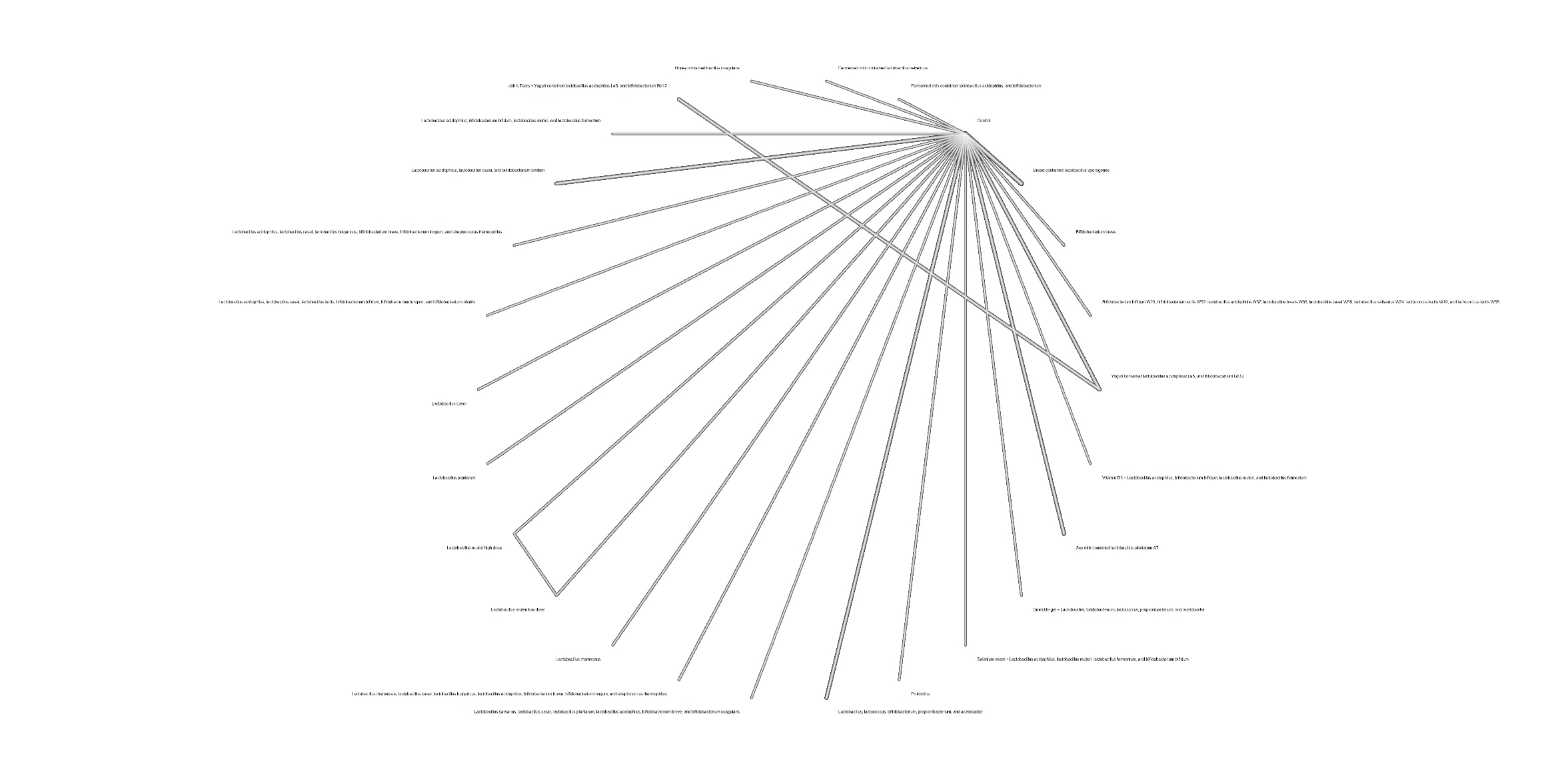

Supplement: Supplementary file 2 — Additional file 2. [file 13098_2025_1841_MOESM2_ESM.docx]
