# Supplementary material for: Network meta-analysis of randomized control trials evaluating the effectiveness of various probiotic formulations in patients with type 2 diabetes mellitus
Source: Diabetol Metab Syndr. 2025 Jul 11;17:265. doi: 10.1186/s13098-025-01841-2 (PMC12254980; doi:10.1186/s13098-025-01841-2)
Supplement: Supplementary file 3 — Additional file 3. [file 13098_2025_1841_MOESM3_ESM.docx]

**1. Insulin concentration**

**Quantifying heterogeneity / inconsistency:**

tau^2 = 0; tau = 0; I^2 = 0% [0.0%; 89.6%]

**Tests of heterogeneity (within designs) and inconsistency (between designs):**

Q d.f. p-value

Total 1.99 2 0.3696


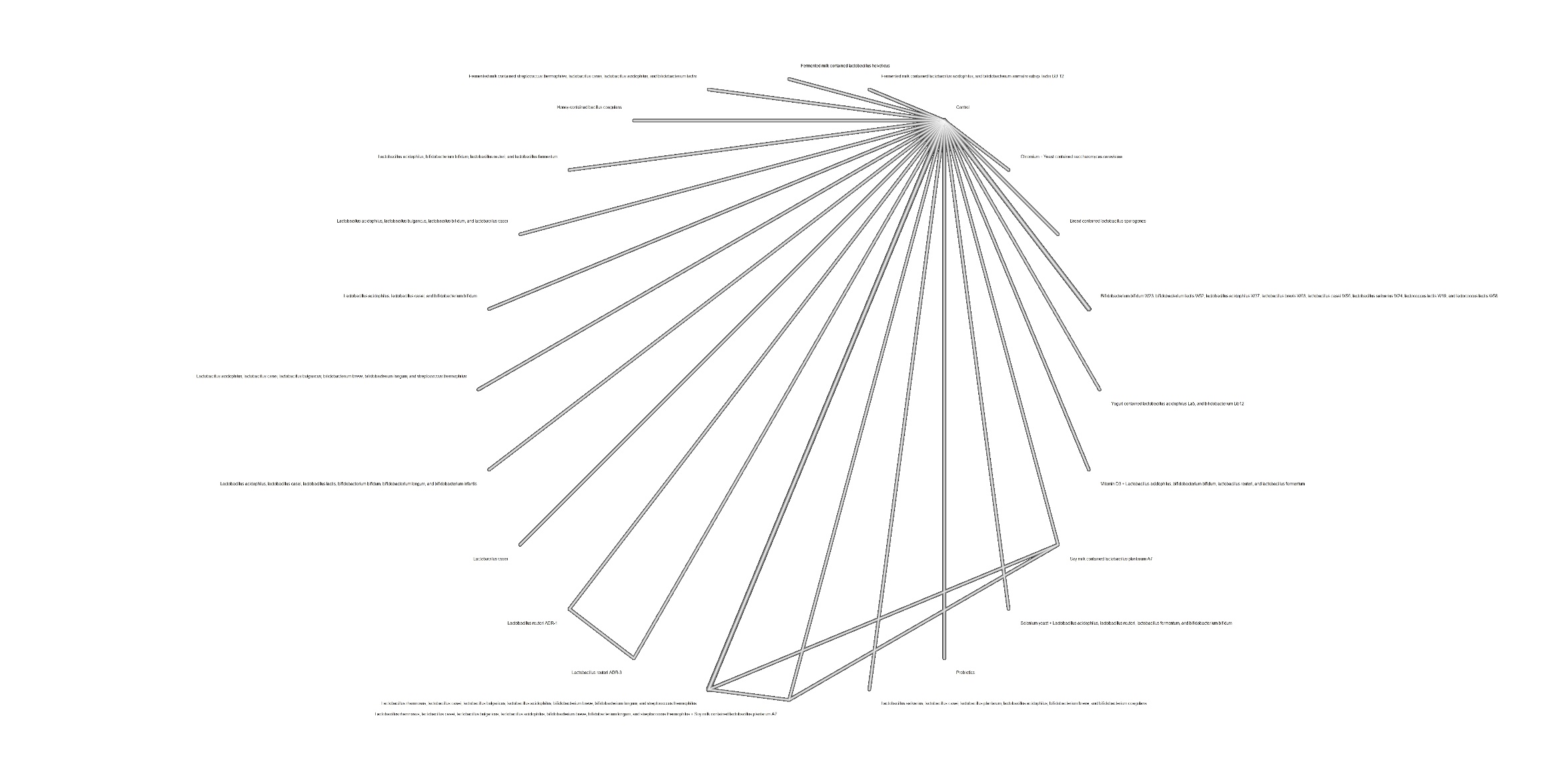


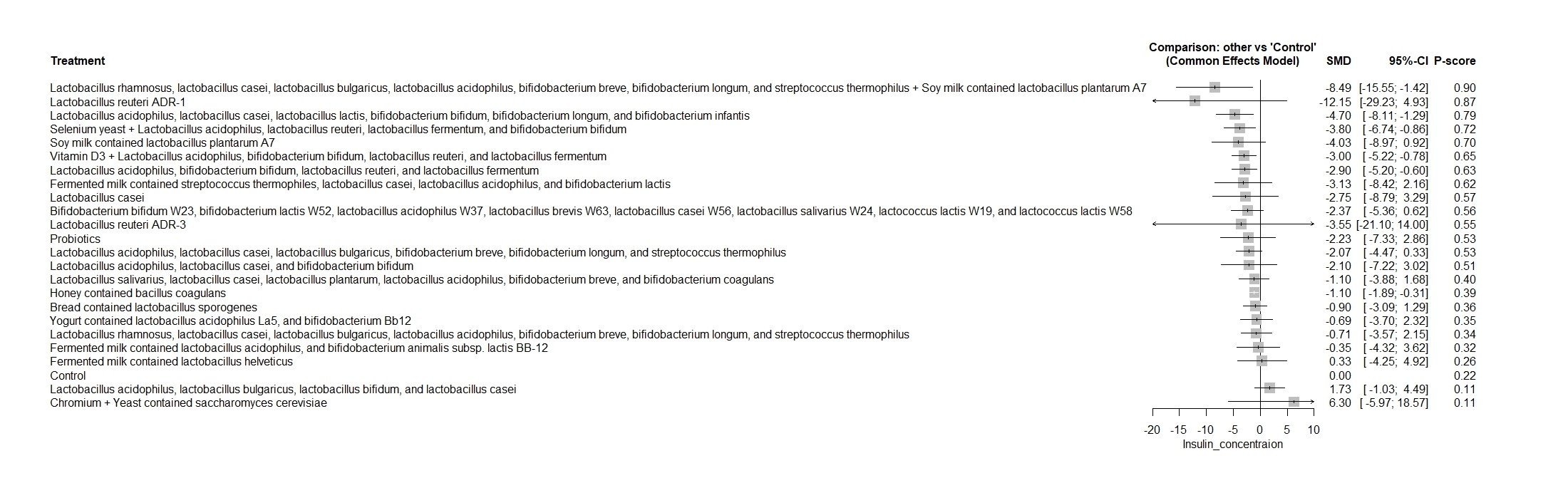

**2. Fasting insulin**

**Quantifying heterogeneity / inconsistency:**

tau^2 = NA; tau = NA

**Tests of heterogeneity (within designs) and inconsistency (between designs):**

Q d.f. p-value

Total 0 0 --

**
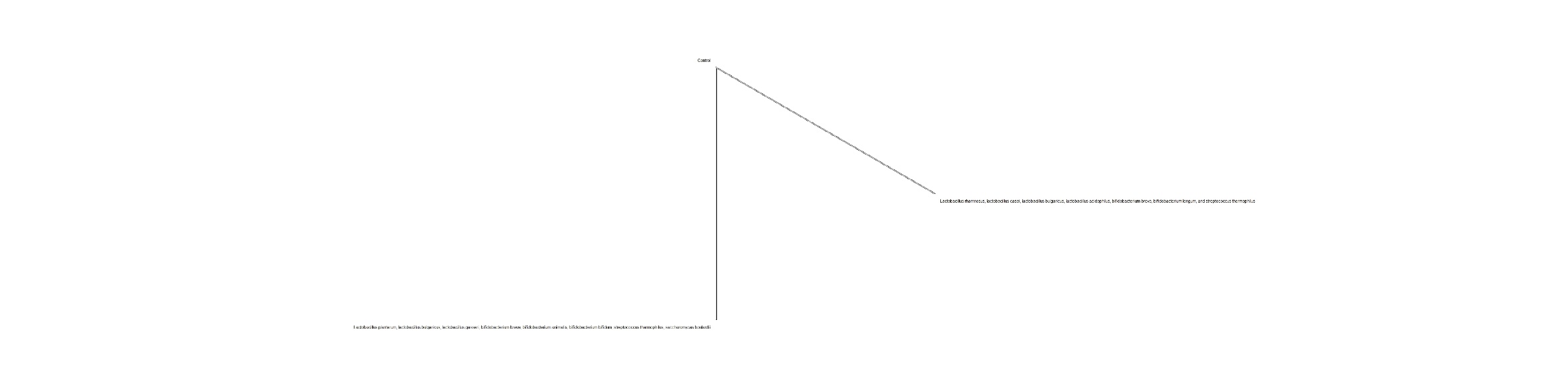
**

**
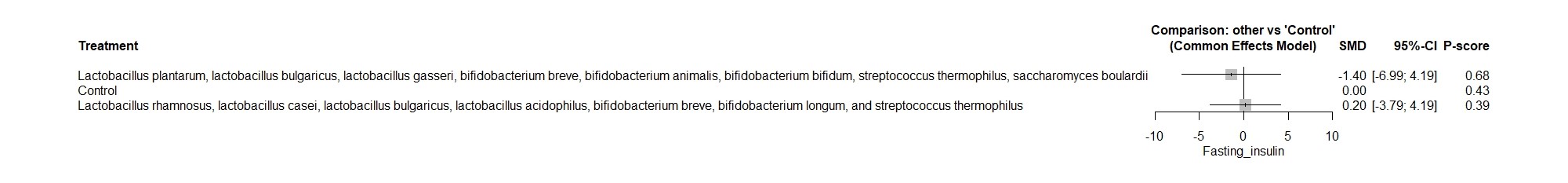
**

**3. PPG**

**Quantifying heterogeneity / inconsistency:**

tau^2 = NA; tau = NA

**Tests of heterogeneity (within designs) and inconsistency (between designs):**

Q d.f. p-value

Total 0 0 --

**
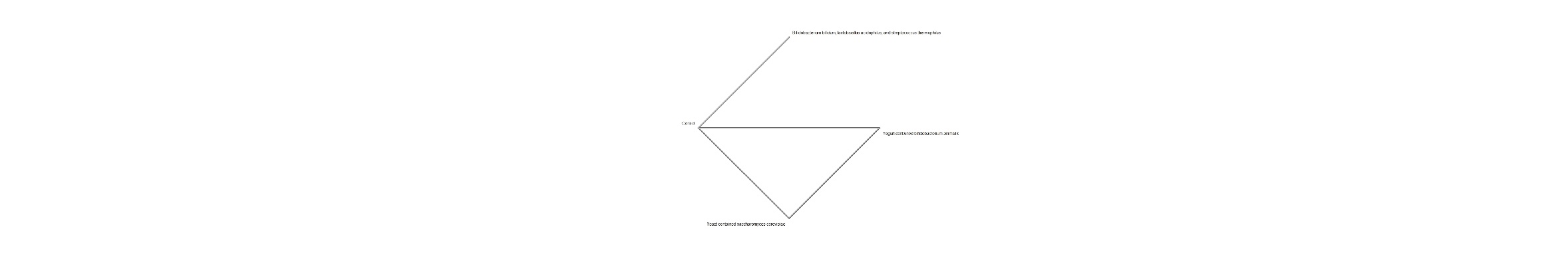
**

**
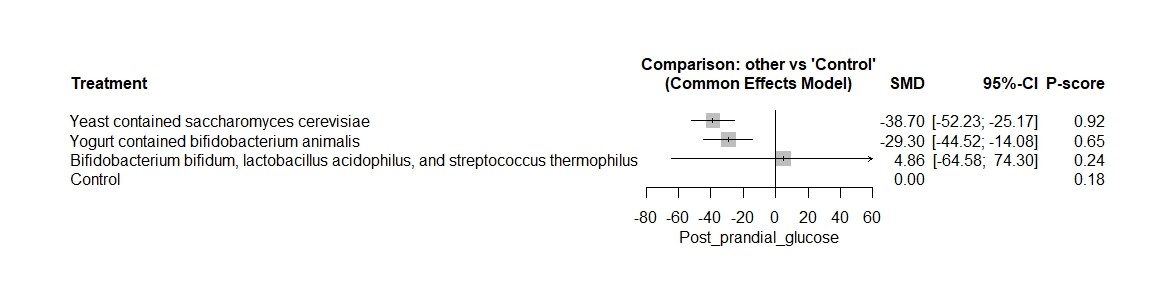
**

**4. Cholesterol \ HDL ratio**

**Quantifying heterogeneity / inconsistency:**

tau^2 = 0; tau = 0; I^2 = 0%

**Tests of heterogeneity (within designs) and inconsistency (between designs):**

Q d.f. p-value

Total 0.77 1 0.3806

**
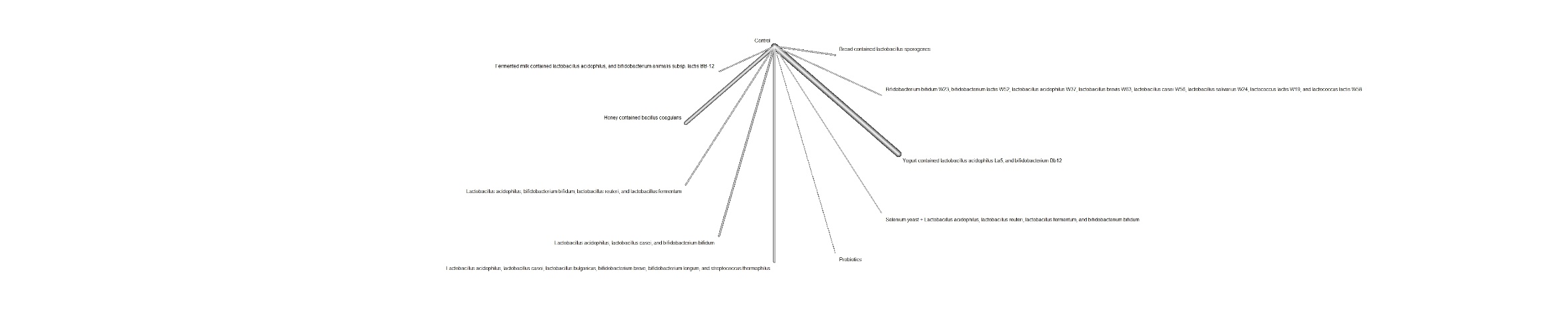
**

**
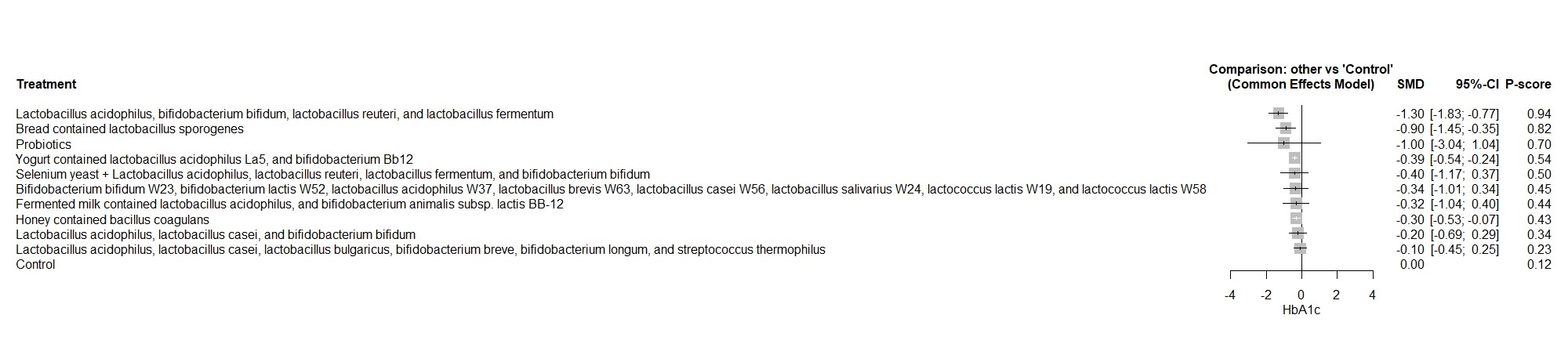
**

**5. CRP**

**Quantifying heterogeneity / inconsistency:**

tau^2 = 0; tau = 0; I^2 = 0% [0.0%; 84.7%]

**Tests of heterogeneity (within designs) and inconsistency (between designs):**

Q d.f. p-value

Total 1.28 3 0.7342


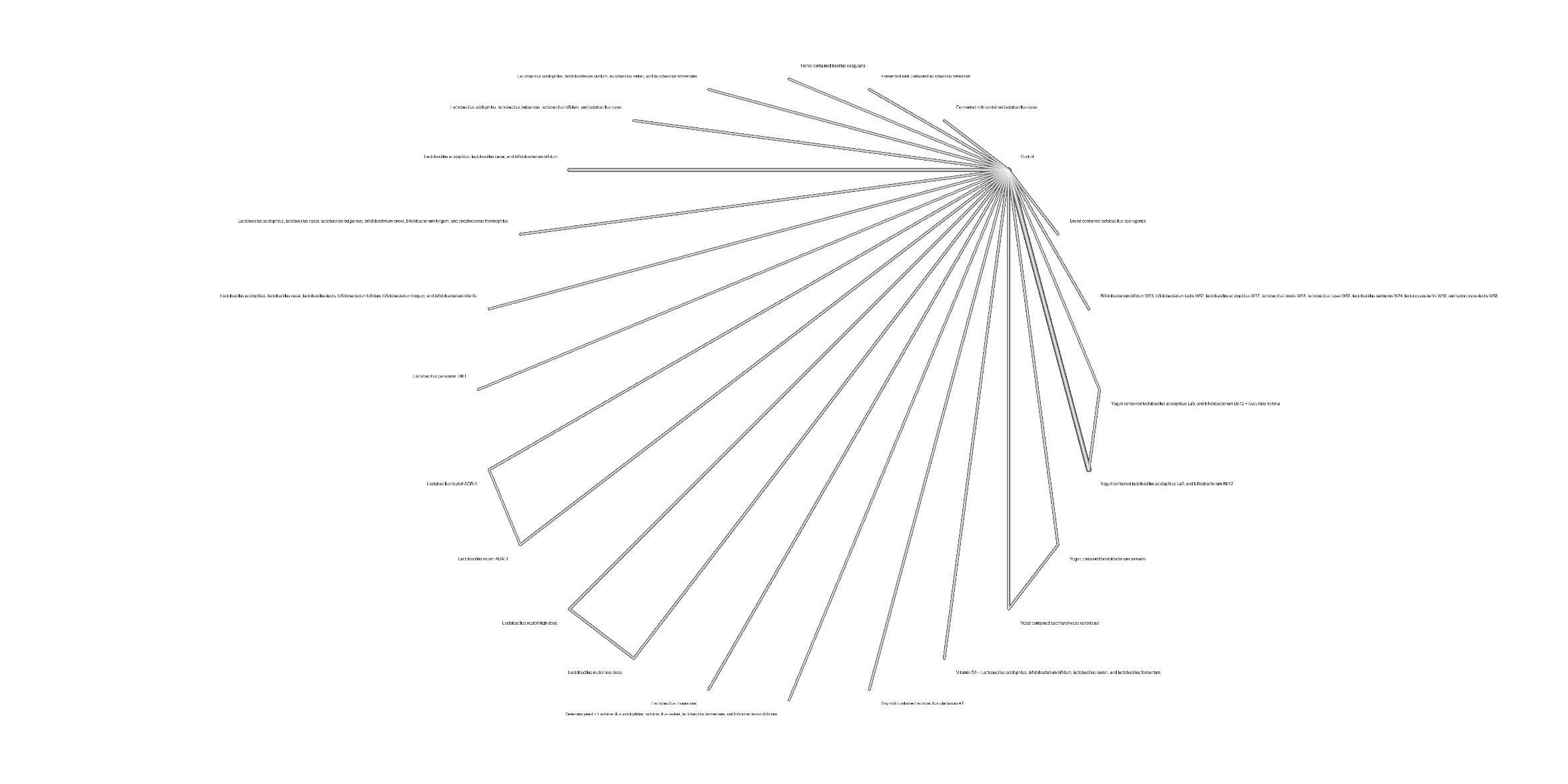


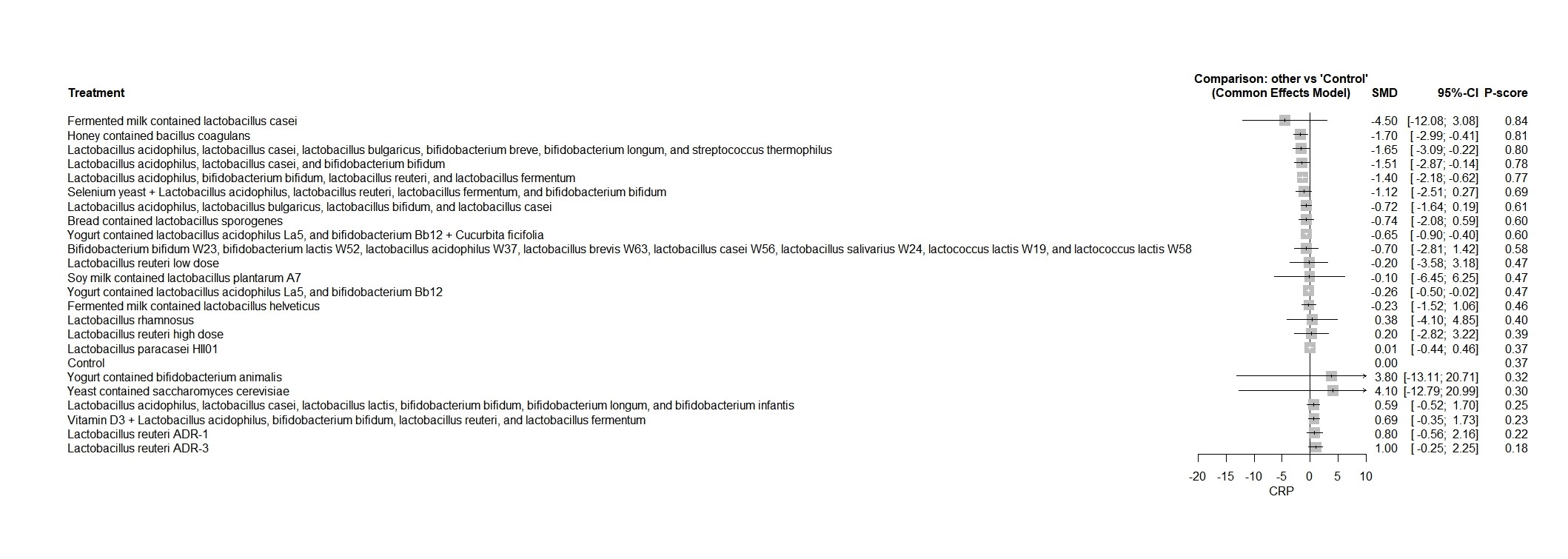

**6. IL-6**

**Quantifying heterogeneity / inconsistency:**

tau^2 = 0; tau = 0; I^2 = 0%

**Tests of heterogeneity (within designs) and inconsistency (between designs):**

Q d.f. p-value

Total 0.42 1 0.5174


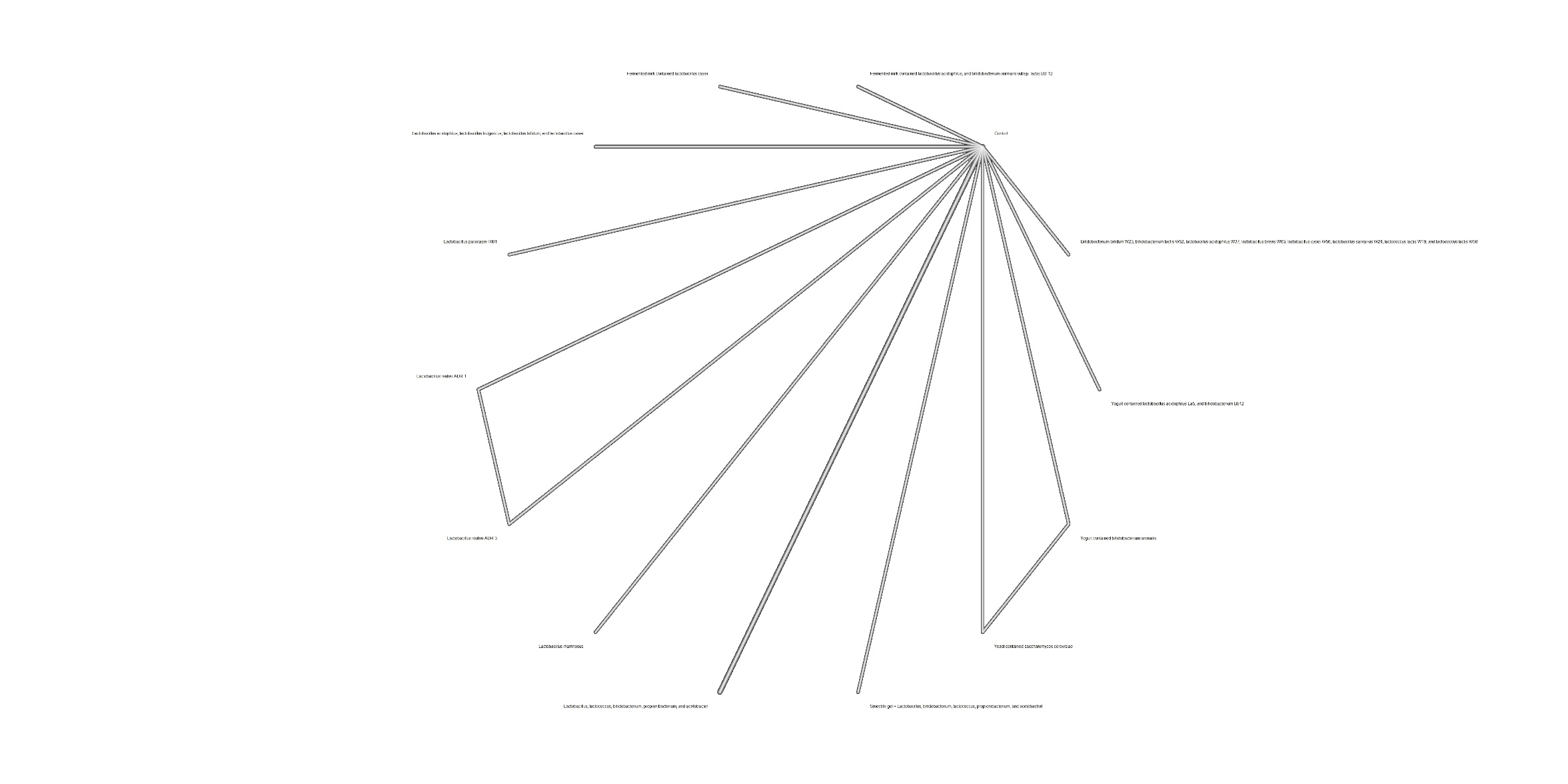


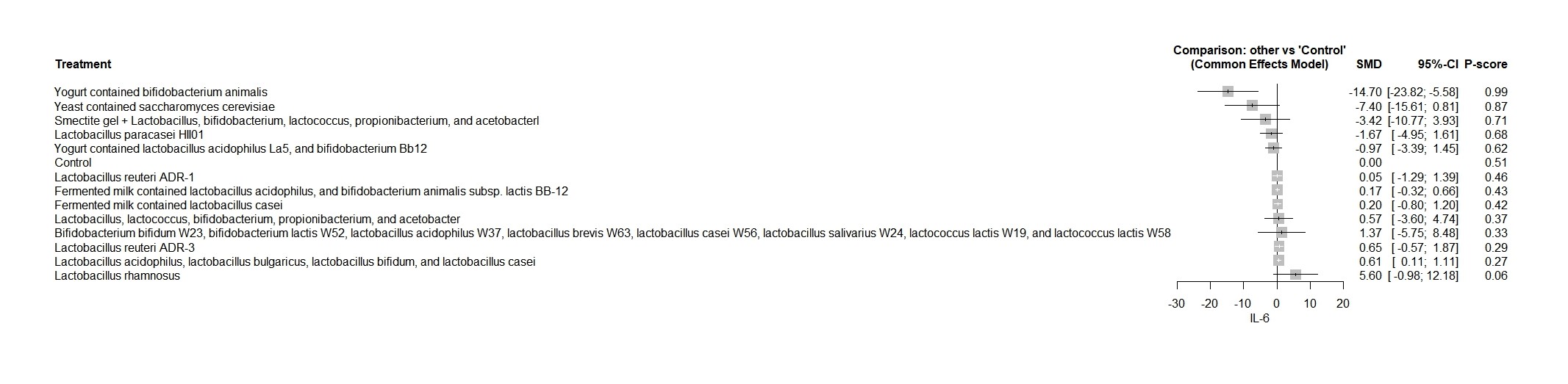

**7. TNF-α**

**Quantifying heterogeneity / inconsistency:**

tau^2 = NA; tau = NA

**Tests of heterogeneity (within designs) and inconsistency (between designs):**

Q d.f. p-value

Total 0 0 --


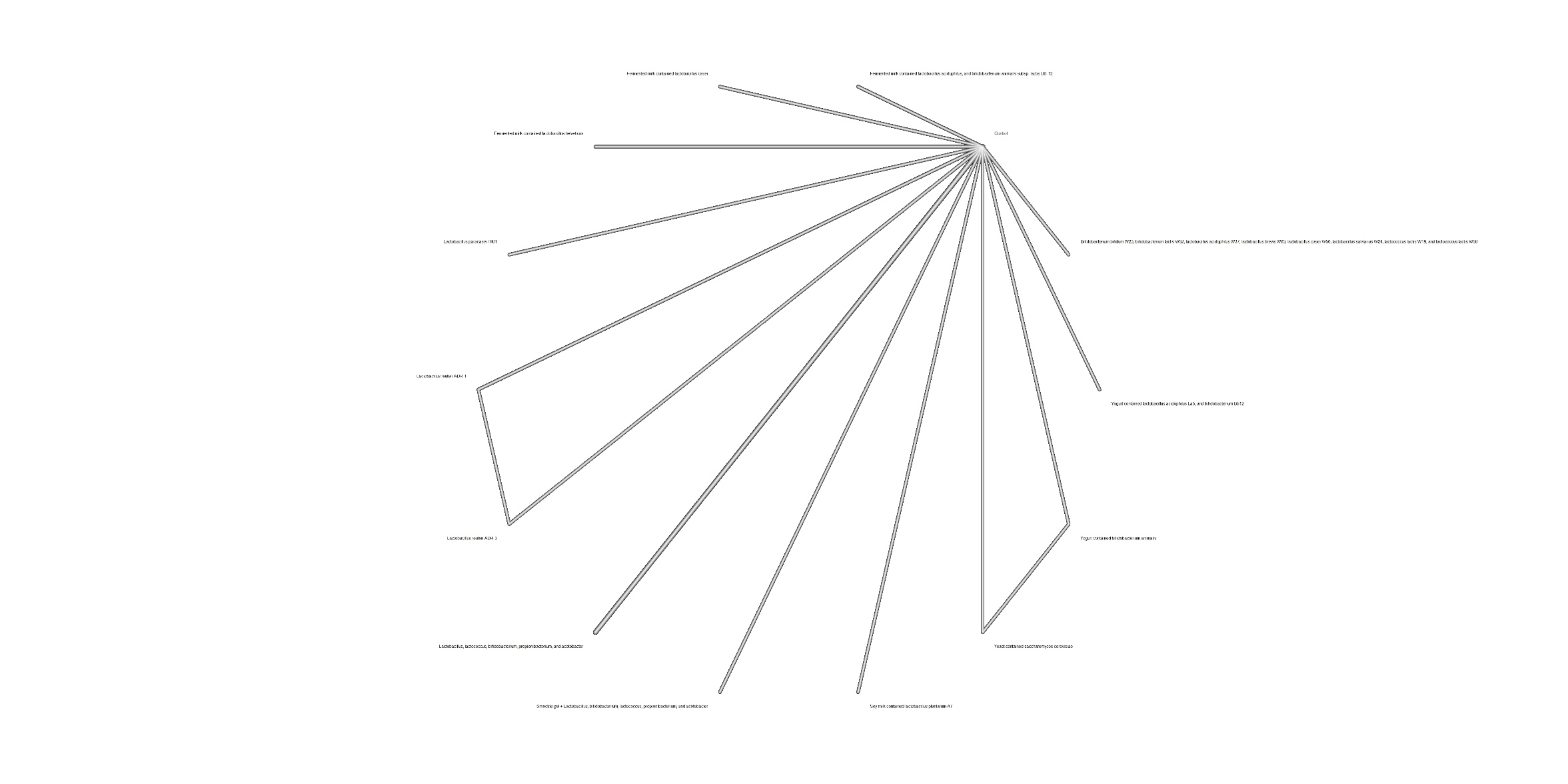


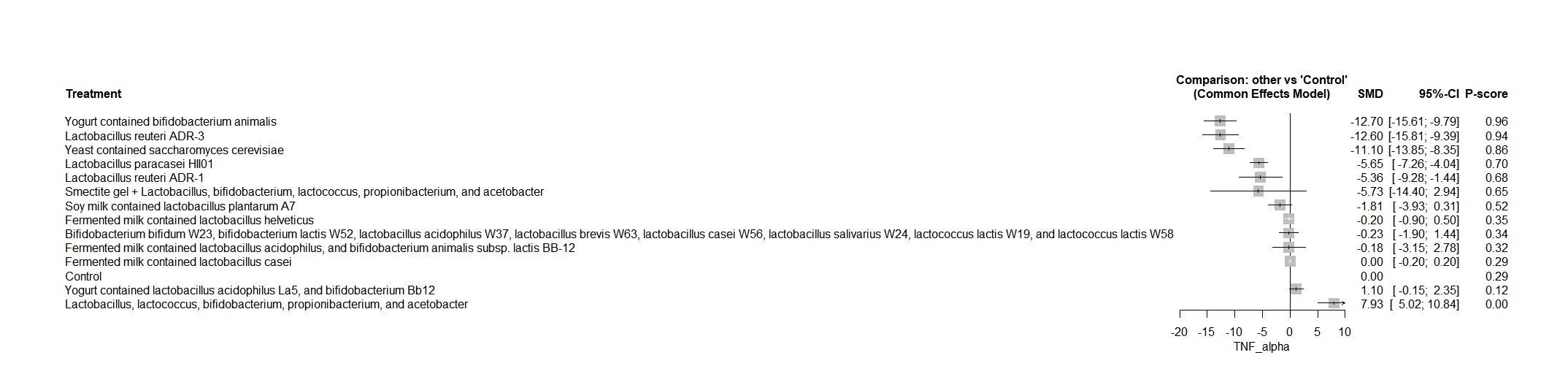

**8. Adiponectin**

**Quantifying heterogeneity / inconsistency:**

tau^2 = NA; tau = NA

**Tests of heterogeneity (within designs) and inconsistency (between designs):**

Q d.f. p-value

Total 0 0 --

**
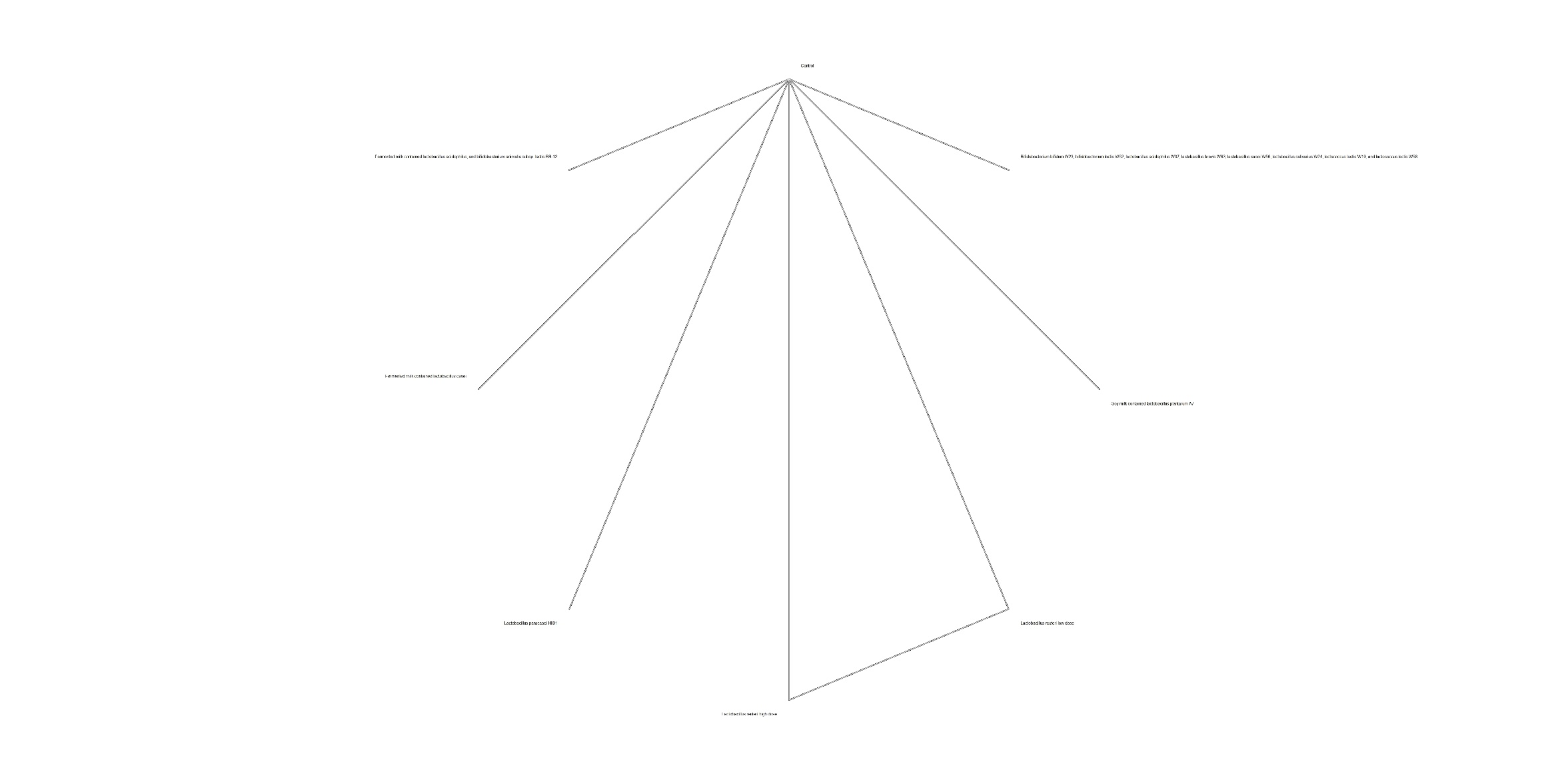
**

**
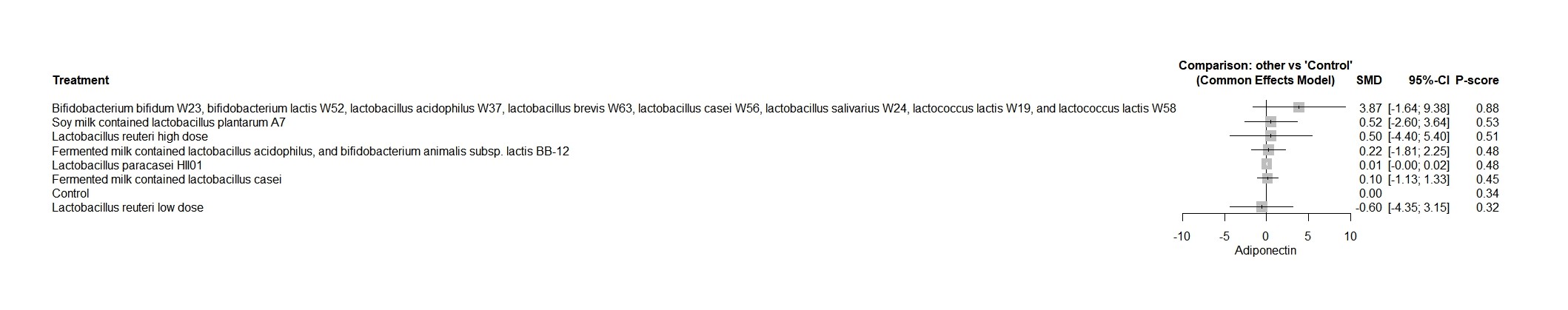
**

**9. Leptin**

**Quantifying heterogeneity / inconsistency:**

tau^2 = NA; tau = NA

**Tests of heterogeneity (within designs) and inconsistency (between designs):**

Q d.f. p-value

Total 0 0 --

**
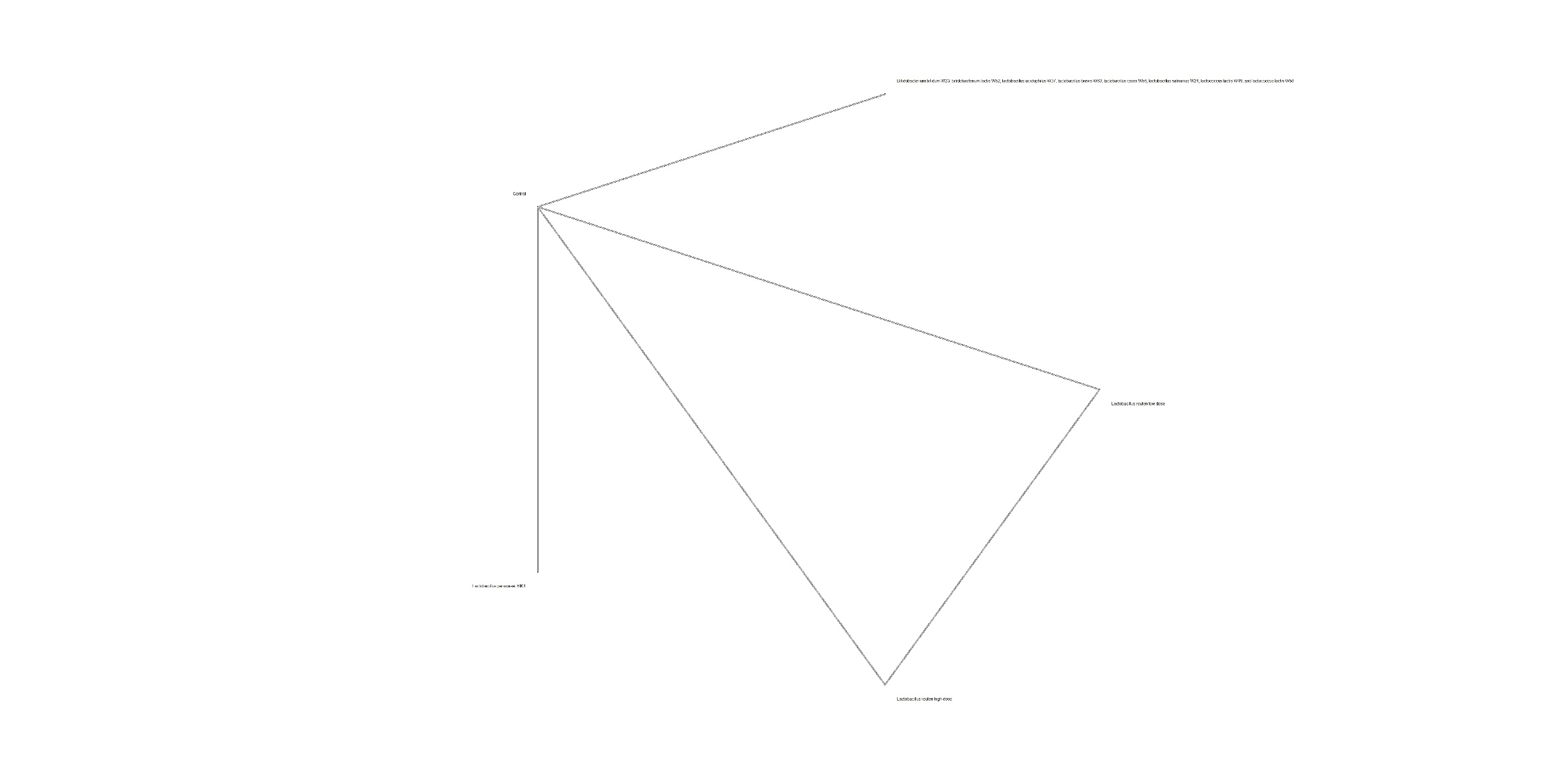
**

**
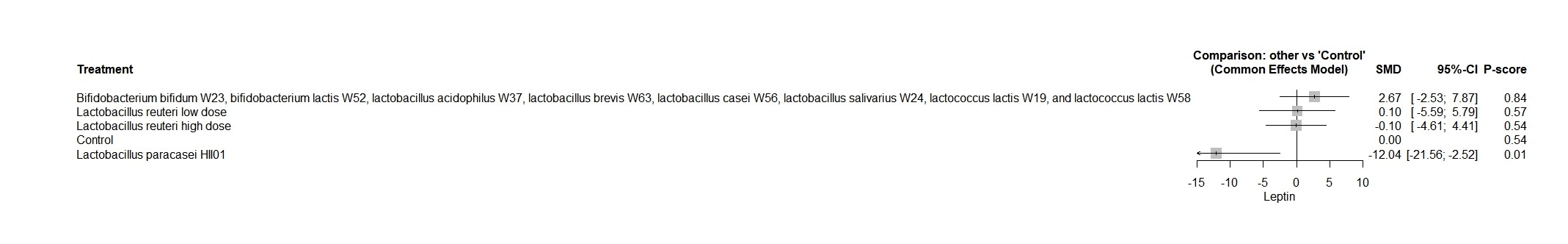
**

**10. Resistin**

**Quantifying heterogeneity / inconsistency:**

tau^2 = NA; tau = NA

**Tests of heterogeneity (within designs) and inconsistency (between designs):**

Q d.f. p-value

Total 0 0 --

**
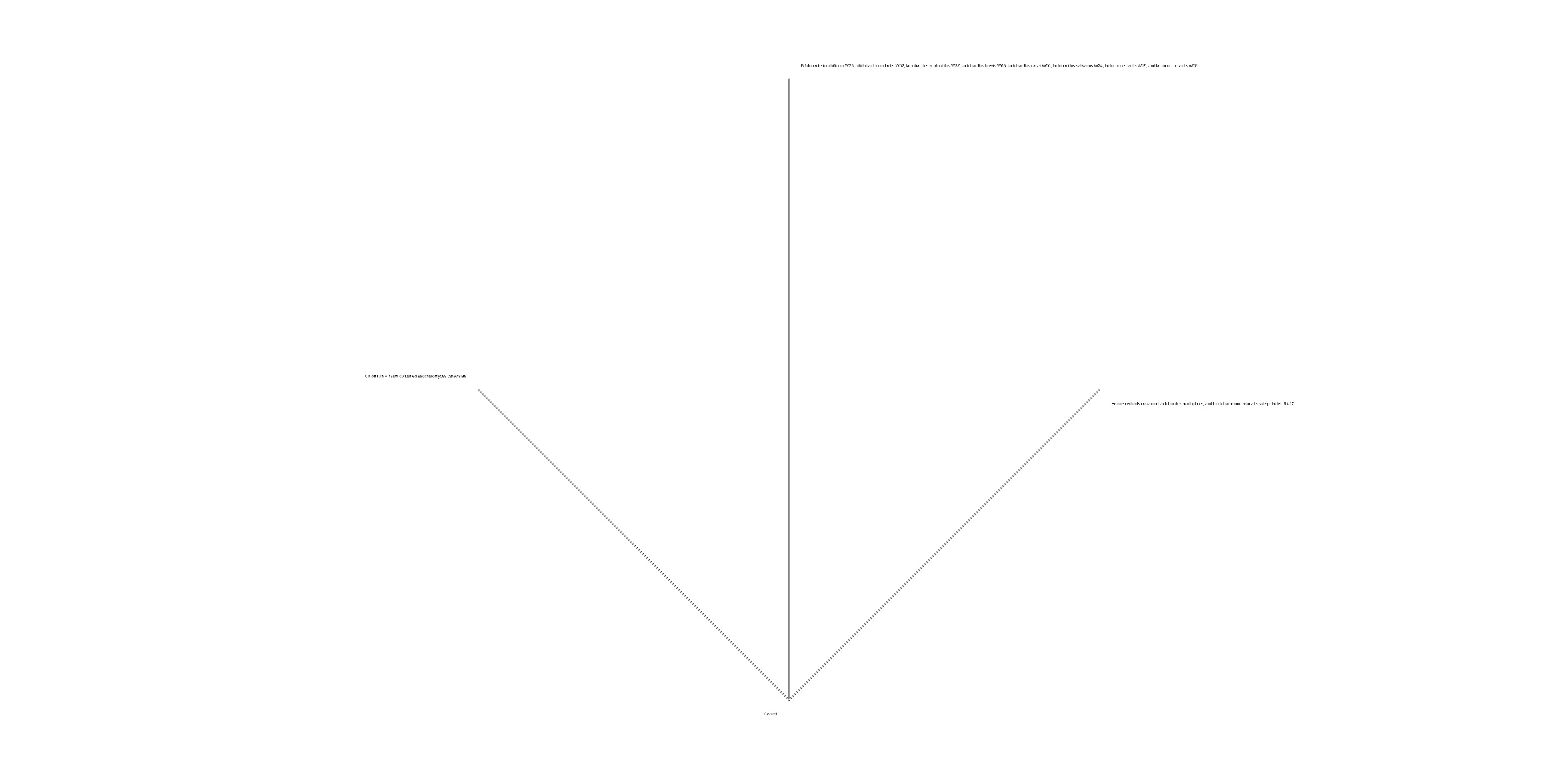
**

**
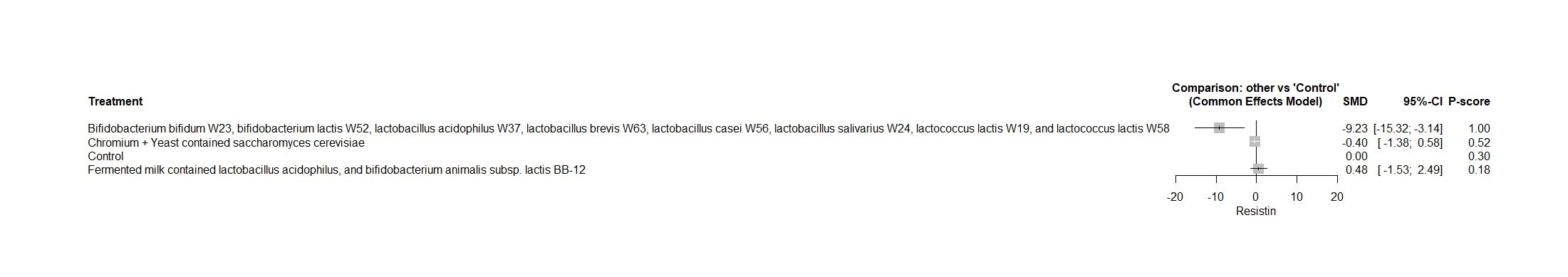
**

**11. Fat mass**

**Quantifying heterogeneity / inconsistency:**

tau^2 = 0; tau = 0; I^2 = 0%

**Tests of heterogeneity (within designs) and inconsistency (between designs):**

Q d.f. p-value

Total 0.03 1 0.8541

**
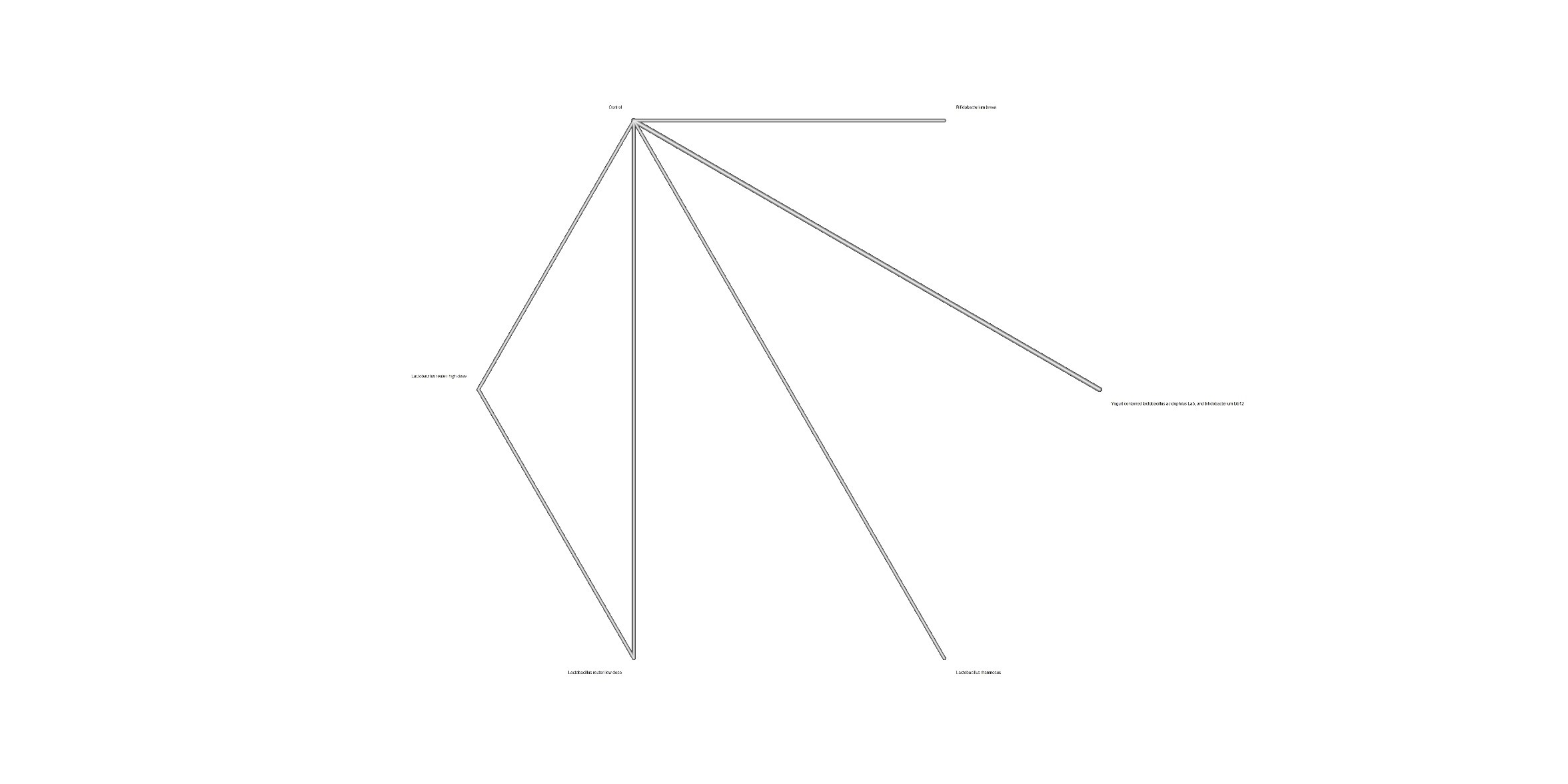
**

**
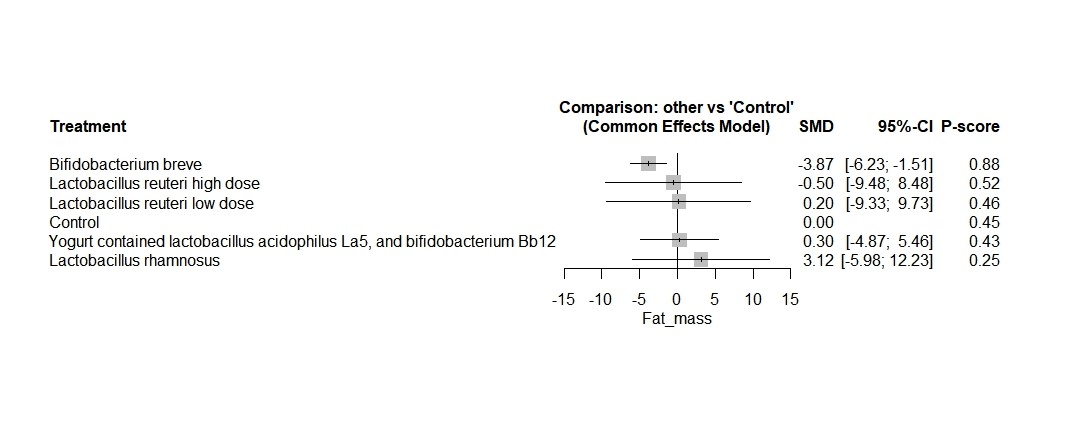
**
