# Supplementary material for: Network meta-analysis of randomized control trials evaluating the effectiveness of various probiotic formulations in patients with type 2 diabetes mellitus
Source: Diabetol Metab Syndr. 2025 Jul 11;17:265. doi: 10.1186/s13098-025-01841-2 (PMC12254980; doi:10.1186/s13098-025-01841-2)
Supplement: Supplementary file 4 — Additional file 4. [file 13098_2025_1841_MOESM4_ESM.docx]

(“Diabetes Mellitus, Noninsulin-Dependent” OR “Ketosis-Resistant Diabetes Mellitus” OR “Diabetes Mellitus, Non-Insulin Dependent” OR “Diabetes Mellitus, Non-Insulin-Dependent” OR “Non-Insulin-Dependent Diabetes Mellitus” OR “Diabetes Mellitus, Stable” OR “Stable Diabetes Mellitus” OR “Diabetes Mellitus, Type II” OR NIDDM OR “Diabetes Mellitus, Noninsulin Dependent” OR “Diabetes Mellitus, Maturity-Onset” OR “Diabetes Mellitus, Maturity Onset” OR “Maturity-Onset Diabetes Mellitus” OR “Maturity Onset Diabetes Mellitus” OR MODY OR “Diabetes Mellitus, Slow-Onset” OR “Diabetes Mellitus, Slow Onset” OR “Type 2 Diabetes Mellitus” OR “Noninsulin-Dependent Diabetes Mellitus” OR “Noninsulin Dependent Diabetes Mellitus” OR “Maturity-Onset Diabetes” OR “Diabetes, Maturity-Onset” OR “Maturity Onset Diabetes” OR “Type 2 Diabetes” OR “Diabetes, Type 2” OR “Diabetes Mellitus, Adult-Onset” OR “Adult-Onset Diabetes Mellitus” OR “Diabetes Mellitus, Adult Onset”) AND (Lactobacillus OR Bifidobacterium OR “Saccharomyces boulardii” OR “Saccharomyces boulardius” OR “boulardii, Saccharomyces” OR “boulardius, Saccharomyces” OR Probiotic*)
